# Elevated Interarm Systolic Blood Pressure Difference Is Positively Associated with Increased Likelihood of Coronary Artery Disease

**DOI:** 10.1155/2021/5577957

**Published:** 2021-07-21

**Authors:** Min Li, Fangfang Fan, Yan Zhang, Wei Ma, Yong Huo

**Affiliations:** ^1^Department of Cardiology, Peking University First Hospital, Beijing, China; ^2^Key Laboratory of Molecular Cardiovascular Sciences (Peking University), Ministry of Education, Beijing, China; ^3^Echocardiography Core Lab, Institute of Cardiovascular Disease at Peking, University First Hospital, Beijing, China

## Abstract

**Background:**

Systolic interarm differences in blood pressure have been associated with all-cause mortality and cardiovascular disease. We investigated the relationship between interarm systolic blood pressure difference and coronary artery disease.

**Methods:**

We retrospectively analyzed data for patients undergoing coronary angiography and brachial-ankle pulse wave velocity examination during hospitalization from 2013 to 2018. Patients underwent simultaneous upper arm blood pressure measurement. Interarm systolic blood pressure difference (IASBPD) was defined as the absolute value of the difference between the right and left upper limb systolic blood pressure. Patients with IASBPD ≥10 mmHg constituted the high group, and those with IASBPD <10 mmHg constituted the normal group. We also recorded data for cardiovascular risk factors. Coronary artery disease was defined as ≥50% vessel stenosis or having undergone interventional therapy according to coronary angiography results.

**Results:**

Compared with the normal group, the number of patients with coronary artery disease was higher in the high group (86.1% vs. 74.6%, *P*=0.029). Multiple logistic regression showed that IASBPD ≥10 mmHg were positively correlated with coronary artery disease (odds ratio, 2.313; 95% confidence interval, 1.086–4.509; *P*=0.029), and as the IASBPD value increased, the correlation also gradually increased.

**Conclusions:**

IASBPD ≥10 mmHg was positively related to coronary artery disease and increased IASBPD values were correlated with coronary artery disease severity.

## 1. Introduction

Interarm systolic blood pressure difference (IASBPD) is gaining research attention nowadays. The UK National Institute for Health and Care Excellence hypertension guidelines clearly state that blood pressure should be measured in both upper arms in the diagnosis of hypertension and propose a normal value for IASBPD of <15 mmHg [1]. Furthermore, some studies have shown that the risk factors for cardiovascular disease such as age, body mass index (BMI), hypertension, and carotid intima-media thickness are related to IASBPD ≥10 mmHg [2]. A Japanese study suggested that IASBPD ≥5 mmHg was significantly associated with cardiovascular events [3], and a meta-analysis showed that IASBPD ≥10 mmHg or IASBPD ≥15 mmHg increased mortality in patients with cardiovascular disease [4]. High IASBPD increases the degree of coronary atherosclerosis [5] as well as the risk of peripheral vascular and cerebrovascular diseases [6].

The reported prevalence of IASBPD ≥10 mmHg is 19.6% in the population [7]. Its high prevalence and predictive effect on cardiovascular disease indicate that measuring IASBPD requires more attention. However, although 77% of doctors realized that blood pressure in both upper limbs should be evaluated during initial hypertension assessments, only 30% agreed with the recommendation, and 13% adhered to the recommendation [8]. Few studies have evaluated the relationship between IASBPD and coronary artery disease. Therefore, we aimed to explore the relationship between these, so as to guide future clinical work.

## 2. Methods

### 2.1. Population

We retrospectively enrolled patients treated in the Department of Cardiology of the Peking University First Hospital from 2013 to 2018. Patients who underwent both coronary angiography and brachial-ankle pressure wave velocity (ba-PWV) examinations during hospitalization were included in this study. We identified 1022 patients with complete data. Patients with stable angina pectoris accounted for 17.8%, acute myocardial infarction accounted for 2.8%, unstable angina pectoris accounted for 67.2%, and the remaining patients had atypical clinical symptoms, such as chest tightness, suffocation, palpitations, and other symptoms. Patients who have undergone repeat angiography accounted for 2.3% of the 1022 patients. This study was approved by the ethics committee of Peking University First Hospital.

### 2.2. Blood Pressure Measurement

The BP-203RPEIII noninvasive disease screening instrument (Omron Healthcare Inc., Kyoto, Japan) was used to measure blood pressure simultaneously in the left and right arm [9]. IASBPD was defined as the absolute value of the systolic pressure difference between the right and left upper limbs. IASBPD ≥10 mmHg was defined as the high IASBPD group [10], and IASBPD <10 mmHg was defined as the normal group.

### 2.3. Definition of Cardiovascular Risk Factors

We collected patients' data describing cardiovascular disease related risk factors such as height, weight, BMI, and blood lipid levels [11]. Current smokers and those with a history of smoking were defined as smokers. Hypertension was defined by the patient's history and systolic blood pressure ≥140 mmHg and/or diastolic blood pressure ≥90 mmHg measured with the BP-203RPEIII instrument (when the systolic blood pressure in the upper limbs was inconsistent, the higher value was selected) [12]. Diabetes was defined according to patients' medical history. No matter taking lipid-lowering drugs or not, triglyceride ≥2.3 mmol/L was defined as hypertriglyceridemia; high-density lipoprotein cholesterol (HDL-C) <1.0 mmol/L was defined as low HDL-C; and low-density lipoprotein cholesterol (LDL-C) ≥4.1 mmol/L was defined as high LDL-C [13]. BMI 24–28 kg/m^2^ defined overweight, and BMI ≥28 kg/m^2^ defined obesity. We investigated the use of antihypertensive drugs in hypertension patients. We also recorded whether patients with hyperlipidemia were taking oral lipid-lowering drugs (statins or others).

### 2.4. Definition of Coronary Artery Disease

Coronary artery disease was defined as ≥50% coronary artery stenosis according to coronary angiography results or having a history of percutaneous coronary interventional. According to the degree of stenosis of the main vessel diameter during coronary angiography, we divided coronary artery disease into three categories: mild, moderate, and severe (≥ 50%−70% , ≥ 70%−90%, and ≥ 90% , respectively). Stenosis < 50%  was defined as coronary atherosclerosis, and absent coronary stenosis defined as normal coronary arteries. Patients who have undergone repeat coronary angiography were evaluated according to the most serious intervention results in the past.

### 2.5. Statistical Analysis

Normally distributed data are shown as mean ± standard deviation, and Student's *t* test was used for comparisons between the groups. Numerical data were expressed as percentages (%), and the chi-square test was used for comparisons between groups. We performed univariate logistic regression to analyze the association between IASBPD, age, sex, BMI, hypertension, diabetes, smoking, triglycerides, LDL-C, HDL-C, and coronary artery disease. We performed multiple logistic regression to analyze the relationship between different IASBPD values and coronary artery disease adjusting for age, sex, BMI, hypertension, diabetes, smoking, LDL-C, HDL-C, triglyceride, antihypertension drugs, and lipid-lowering drugs. We divided coronary artery disease into five groups according to the coronary angiography results as follows: normal coronary artery, coronary atherosclerosis, mild stenosis, moderate stenosis, and severe stenosis, and used ordinal logistic regression to study the relationship between IASBPD and coronary artery disease severity. Subgroup analyses and interaction tests were used to examine the IASBPD and CHD according to sex, age, BMI, prevalence of hypertension, diabetes, smoking, triglycerides, HDL-C, and LDL-C. *P* values <0.05 were considered statistically significant according to two-tailed analysis. All analyses were performed using SPSS software, version 25.0 (IBM Corp., Armonk, NY).

## 3. Results

The baseline characteristics of all participants are shown in Table 1 as overall characteristics and according to the IASBPD group. Participants' mean age was 63 ± 10 years, and men accounted for 62% of the patients. Mean body weight and BMI were higher in the high IASBPD group (*P* < 0.05). The prevalence of coronary artery disease was also higher in the high vs. normal group (86.1% vs. 74.6%, respectively; *P* < 0.05), whereas the ankle-brachial index was lower in the high IASBPD group. Other cardiovascular risk factors such as age, lipid levels, smoking, hypertension, and diabetes were not of statistical difference between the two groups.

The results of the univariate logistic regression showed that IASBPD ≥10 mmHg (OR, 2.136; 95% CI, 1.052–4.338; *P*=0.036), age (OR, 1.020; 95% CI, 1.004–1.036; *P*=0.015), male sex (OR, 2.184; 95% CI, 1.461–3.265; *P* < 0.001), overweight (OR, 1.529; 95% CI, 1.018–2.298; *P*=0.041), obesity (OR, 1.632; 95% CI, 1.144–2.328, *P*=0.007), hypertension (OR, 1.873; 95% CI, 1.347–2.604; *P* < 0.001), and diabetes (OR, 1.601; 95% CI, 1.154–2.222; *P*=0.005) were significantly correlated with coronary artery disease (the detailed information is presented in Supplementary Table 1).

Multiple regression analysis showed that IASBPD ≥10 mmHg was significantly correlated with coronary artery disease (OR, 2.313; 95% CI, 1.086–4.509; *P*=0.029) when adjusted for age, sex, overweight, obesity, hypertension, diabetes, smoking, high LDL-C, low HDL-C, high triglyceride, antihypertension drugs, and lipid-lowering drugs (the detailed information is presented in Supplementary Table 2). After adjusting the blood lipid and BMI into continuous variables, the result showed that IASBPD was still significantly related to CHD (odds ratio, 2.049; 95% confidence interval, 1.009–4.160; *P*=0.047). The collinearity diagnosis suggested that there was collinearity between the independent variables, but the goodness of fit test results indicated that the model fit well. Considering that this study mainly predicts the relationship between IASBPD and CHD, the collinearity between the independent variables may not affect the final prediction result.

We further divided IASBPD into IASBPD ≥5 mmHg, IASBPD ≥10 mmHg, and IASBPD ≥15 mmHg to investigate the relationship between different IASBPD values and coronary artery disease. The results of multiple logistic regression are displayed in Table 2. The three IASBPD categories were significantly associated with coronary artery disease, and as the IASBPD value increased, the correlation also gradually increased.

Ordinal logistic regression showed that, compared with the normal IASBPD group, the odds ratio for severe stenosis was 1.950 times greater compared with the combined odds ratios for normal coronary artery, coronary atherosclerosis, and mild and moderate stenosis categories in patients with IASBPD ≥10 mmHg (95% CI, 1.220–3.117; *P*=0.005). Male sex, hypertension, diabetes, and LDL-C ≥4.1 mmol/L were also significantly associated with coronary stenosis severity (the results are displayed in Table 3).

**Figure 1:**
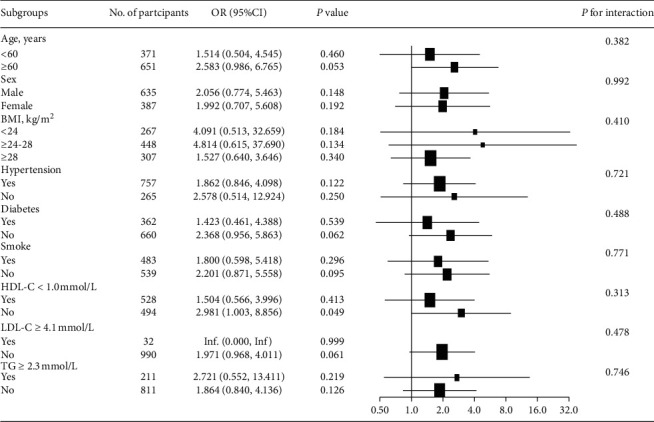
Subgroup analysis for the relationship between IASBPD and coronary artery disease. BMI, body mass index; CI, confidence interval; HDL-C, high-density lipoprotein cholesterol; IASBPD, interarm systolic blood pressure difference; LDL-C, low-density lipoprotein cholesterol; OR, odds ratio; TG, triglycerides.

Subgroup analyses results are presented in Figure 1. No significant heterogeneity was found among all analyzed subgroups according to sex, age, BMI, prevalence of hypertension, diabetes, smoking, triglycerides, HDL-C, and LDL-C.

## 4. Discussion

Hypertension is one of the common risk factors for coronary heart disease. Benefits of hypertension treatment are greatest for individuals with the highest estimated cardiovascular risk. IASBPD has the characteristics of simple measurement method and low cost. It is hoped that, through this inspection, people who benefit from early preventive measures will be screened out. Our study further investigated the relationship between IASBPD and coronary artery disease, which showed that IASBPD ≥10 mmHg was independently related to coronary artery disease. IASBPD ≥5 mmHg was significantly correlated with coronary artery disease in our study, consistent with previous research results, and increased IASBPD values were correlated with coronary artery disease severity.

The results of our study showed that IASBPD ≥10 mmHg was independently associated with coronary artery disease, but there still remains controversy about this conclusion. An American study involving 3390 patients showed that IASBPD ≥10 mmHg and cardiovascular events were independently correlated (HR 1.38; 95% CI, 1.09–1.75) [14]. INTERPRESS-IPD research including 53827 participants showed that IASBPD was associated with cardiovascular mortality (HR 1.07; 95% CI, 1.03–1.12) per 5 mmHg [15]. However, a Japanese retrospective study involving 425 patients revealed no correlation between IASBPD and coronary artery disease [16]; the authors did not explain the reason for this conclusion, stating that they considered the lower prevalence of IASBPD ≥10 mmHg (8.7%) may have contributed to this finding, while the prevalence of IASBPD ≥10 mmHg was 7.05% in our study.

The exact mechanism for the relationship between IASBPD and coronary artery disease is not yet clear. Some studies showed that high IASBPD was related to atherosclerosis and left ventricular mass index [17]. IASBPD increased the degree of vascular stiffness [18]. IASBPD ≥10 mmHg was also independently related to intima-media thickness [19], ankle-brachial index <0.9, and high PWV values [17]. Therefore, high IASBPD may lead to coronary artery disease through various mechanisms.

We divided IASBPD values into three groups: IASBPD ≥5 mmHg, IASBPD ≥10 mmHg, and IASBPD ≥15 mmHg, to evaluate the relationship between IASBPD and coronary artery disease. The results showed that as the IASBPD values increased, the relation became strong. A Japanese study involving 700 patients evaluated the best cut-off value for IASBPD to predict cardiovascular events and found that IASBPD ≥5 mmHg was the best [3]. We found similar results when we used IASBPD ≥5 mmHg. However, the definition of IASBPD remains controversial. Existing clinical data were mostly based on IASBPD values of 10 mmHg and 15 mmHg. Some studies confirmed that IASBPD ≥10 mmHg increased the incidence of stroke [20] and atherosclerosis [17]. A cross-sectional study showed that the prevalence of stroke and cardiovascular disease increased in patients with IASBPD ≥15 mmHg [21], and a meta-analysis of nine cohort studies indicated that IASBPD ≥10 mmHg and ≥15 mmHg both predicted cardiovascular mortality; therefore, IASBPD ≥15 mmHg can help predict cardiovascular mortality even in the community population [22]. Our results showed that both IASBPD ≥10 mmHg and ≥15 mmHg were significantly correlated with coronary artery disease, and as the defined values increased, the correlation also gradually increased.

It should be emphasized that the correct method of measuring IASBPD is important and that different devices or measurement techniques may lead to different results. Existing methods include sequential measurement and simultaneous measurement; we used simultaneous measurement in this study. Sequential measurement may result in twofold values compared with simultaneous blood pressure measurement (14.6% vs. 6.4%, respectively) [23], which may be caused by the white coat effect. Furthermore, blood pressure is a variable factor, which also increases the error rate by sequential measurement [24]. In summary, simultaneous measurement may be a feasible method for more accurate hypertension diagnosis compared with sequential measurement and more accurately predicts cardiovascular events.

The ordinal logistic regression results showed that, in patients with IASBPD ≥10 mmHg, coronary stenosis was 1.950 times more serious than those with IASBPD <10 mmHg. Male sex, hypertension, diabetes, and LDL-C >4.1 mmol/L were also positively correlated with coronary heart disease severity. In a retrospective study from North Korea involving 816 patients using the Gensini score as a diagnostic criterion to study the relationship between IASBPD and coronary artery disease, multiple regression analysis showed that IASBPD was significantly correlated with the Gensini score (95% CI, 0.018–0.043; *P* < 0.001). The authors also showed that male sex, hypertension, and diabetes were associated with the Gensini score [25]. Similar studies showed that IASBPD was associated with coronary artery disease severity [5, 16]. However, the exact mechanism between IASBPD and coronary artery disease severity is not fully understood. One study showed that IASBPD was a diagnostic indicator of subclinical atherosclerosis in patients with type 2 diabetes [19]. Additionally, high IASBPD may occur most often in patients with subclavian atherosclerosis [26]. A multiple regression analysis of 307 patients with subclavian artery stenosis revealed a significant positive correlation between subclavian artery stenosis and peripheral vascular disease and carotid intima-media thickness [27]. In summary, high IASBPD is associated with peripheral atherosclerosis, which may partly explain how IASBPD indirectly worsens coronary atherosclerosis.

When we divided the patients according to age, sex, BMI, lipid levels, hypertension, diabetes, and other cardiovascular disease risk factors into different subgroups to analyze the relationship between IASBPD and coronary artery disease, we found no statistically significant differences. However, previous studies found the relationship between IASBPD and coronary artery disease in specific populations. For example, a study in Shanghai, China, involving 1528 older (≥65 years old) people showed that IASBPD was a risk factor for cardiovascular disease (*β* = 0.003; *P* < 0.01) [28]. An American study found that, among older community-dwelling adults, IASBPD was associated with arterial stiffness (OR, 1.15; 95% CI, 1.03–1.29; *P*=0.01) [18]. Another study showed that IASBPD was a diagnostic indicator of subclinical atherosclerosis in patients with type 2 diabetes [19]. A British study followed for 9.8 years found that the risk of all-cause death was 3.6 (95% CI, 2.0–6.5) in people with IASBPD ≥10 mmHg in hypertension people [29]. Although our study did not find a similar correlation in older people, or in those with diabetes or hypertension, each subgroup showed a positively correlated trend (OR >1) between IASBPD and coronary artery disease. It is worth noting that, in addition to the traditional coronary artery disease risk factors, in some low-risk populations (hypolipidemia, normal BMI, and <60 years old), IASBPD and coronary artery disease were also positively correlated. To some extent, this suggested that IASBPD may be independently related to coronary artery disease in various populations.

## 5. Limitations

Our study had several limitations. First, this was a retrospective study with no follow-up data and therefore could not investigate the cause and effect of IASBPD and CHD. But it can lay the foundation for subsequent cohort studies. Second, when we collected patients' data, we selected patients whose records included coronary angiography and ba-PWV data collected simultaneously, which may have introduced selection bias. Third, we measured blood pressure in the upper limbs only once in each patient, so the data may have some measurement bias. Despite the above limitations, it does not affect the true connections between the IASBPD and CHD.

## 6. Conclusion

High IASBPD is associated with coronary artery disease and coronary artery disease severity. Few studies have evaluated the relationships between these, and related research failed to accurately explain the underlying mechanism. More studies are required to gain a deeper understanding of the effect of IASBPD on coronary arteries and peripheral blood vessels, to guide better clinical antihypertensive treatment.

## Figures and Tables

**Table 1:** Baseline characteristics of the study cohort.

	All	IASBPD ≥ 10 mmHg	IASBPD ＜ 10 mmHg	*P* value
	(*N* = 1022)	(*N* = 72)	(*N* = 950)	
Male (*n*, %)	635 (62.1%)	47 (65.3%)	588 (61.9%)	0.568
Age	63.3 ± 10.2	64.0 ± 10.1	63.3 ± 10.2	0.565
Height, (cm)	166.0 ± 8.2	165.2 ± 8.2	166.1 ± 8.2	0.374
Weight, (kg)	72.8 ± 12.6	76.1 ± 14.0	72.5 ± 12.4	0.021
BMI, (kg/m^2^)	26.3 ± 3.6	27.8 ± 4.3	26.2 ± 3.6	<0.001
Hypertension (*n*, %)	757 (74.1%)	60 (83.3%)	697 (73.4%)	0.063
Diabetes (*n*, %)	362 (35.4%)	30 (41.7%)	332 (34.9%)	0.250
Smoking (*n*, %)	483 (47.3%)	35 (48.6%)	448 (47.2%)	0.812
TG, (mmol/L)	1.8 ± 1.4	1.9 ± 1.7	1.8 ± 1.4	0.744
TC,( mmol/L)	4.1 ± 1.1	4.2 ± 1.0	4.1 ± 1.1	0.172
HDL-C, (mmol/L)	1.0 ± 0.3	1.0 ± 0.3	1.0 ± 0.3	0.093
LDL-C, (mmol/L)	2.4 ± 0.8	2.4 ± 0.8	2.3 ± 0.8	0.437
TG ＞ 2.3 mmol/L (*n*, %)	211 (20.6%)	14 (19.4%)	197 (20.7%)	0.794
HDL-C ＜ 1.0 mmol/L (*n*, %)	528 (51.7%)	38 (52.8%)	490 (51.6%)	0.844
LDL-C ＞ 4.1 mmol/L (*n*, %)	32 (3.1%)	2 (2.8%)	30 (3.2%)	0.858
ABI	1.1 ± 0.1	1.0 ± 0.1	1.1 ± 0.1	<0.001
ba-PWV, (cm/s)	1610.0 ± 329.3	1668.9 ± 327.4	1605.5 ± 329.1	0.115
Right arm SBP, (mmHg)	127.8 ± 16.2	130.5 ± 18.2	127.6 ± 16.0	0.146
Right arm DBP, (mmHg)	74.0 ± 9.8	73.6 ± 9.4	74.0 ± 9.8	0.761
Left arm SBP, (mmHg)	128.0 ± 16.4	130.8 ± 19.4	127.8 ± 16.1	0.126
Left arm DBP,( mmHg)	73.9 ± 10.1	74.0 ± 11.4	73.9 ± 10.0	0.933
Anti-hypertension (*n*, %)	654 (64.0%)	50 (69.4%)	604 (63.6%)	0.317
Lipid-lowering (*n*, %)	546 (53.4%)	37 (51.4%)	509 (53.6%)	0.719
CHD, (*n*, %)	771 (75.4%)	62 (86.1%)	709 (74.6%)	0.029

ba-PWV: brachial–ankle pulse wave velocity; ABI: ankle–brachial index; BMI: body mass index; CHD: coronary heart disease; DBP: diastolic blood pressure: HDL-C, high-density lipoprotein cholesterol: IASBPD, interarm systolic blood pressure difference; LDL-C: low-density lipoprotein cholesterol; SBP: systolic blood pressure; TC: total cholesterol; TG: triglycerides.

**Table 2:** Relationship between the different IASBPD values and coronary artery disease using a multiple logistic regression model.

	OR (95% CI)	*P* value
IASBPD ≥ 5 mmHg	1.635 (1.155–2.313)	0.006
IASBPD ≥ 10 mmHg	2.313 (1.086–4.509)	0.029
IASBPD ≥ 15 mmHg	3.563 (1.058–11.997)	0.040

Variables in the equation included age, sex, body mass index (BMI), hypertension, diabetes, smoking, low- and high-density lipoprotein cholesterol, triglyceride, antihypertension drugs, and lipid-lowering drugs. CI: confidence interval; IASBPD: interarm systolic blood pressure difference; OR: odds' ratio.

**Table 3:** Ordinal logistic regression for IASBPD ≥10 mmHg and coronary artery disease severity.

	OR (95% CI)	*P* value
Age (≥60 years old)	1.263 (0.987–1.615)	0.063
Sex (female as reference)	2.109 (1.554–2.862)	0.001
BMI, kg/m^2^	0.959 (0.928–0.991)	0.011
Hypertension	1.469 (1.131–1.909)	0.004
Diabetes	1.477 (1.160–1.882)	0.002
Smoking	1.109 (0.826–1.488)	0.492
HDL-C ＜ 1.0 mmol/L	1.267 (0.998–1.608)	0.052
LDL-C ≥ 4.1 mmol/L	2.078 (1.051–4.109)	0.035
IASBPD ≥ 10 mmHg	1.950 (1.220–3.117)	0.005

BMI: body mass index; CI: confidence interval; HDL-C: high-density lipoprotein cholesterol; IASBPD: interarm systolic blood pressure difference; LDL-C: low-density lipoprotein cholesterol; OR: odds' ratio.

## Data Availability

The Excel data used to support the findings of this study are included within the supplementary information file.

## Supplementary Materials

Supplementary Materials Supplementary Table 1: relationship between IASBPD and coronary artery disease using a univariate logistic regression model. Supplementary Table 2: relationship between IASBPD and coronary artery disease using a multiple logistic regression model. Original data: mainly recorded the original data related to this article.
