# Supplementary material for: Elevated Interarm Systolic Blood Pressure Difference Is Positively Associated with Increased Likelihood of Coronary Artery Disease
Source: Int J Hypertens. 2021 Jul 21;2021:5577957. doi: 10.1155/2021/5577957 (PMC8321749; doi:10.1155/2021/5577957)
Supplement: Supplementary Materials — Supplementary Table 1: relationship between IASBPD and coronary artery disease using a univariate logistic regression model. Supplementary Table 2: relationship between IASBPD and coronary artery disease using a multiple logistic regression model. Original data: mainly recorded the original data related to this article. [file 5577957.f1.zip › original data.pdf]

Number	Sex	Age	Height	Weight	BMI（kg/m^	Antihyperten	Lipid-lowerir	BMI（1=No	Hypertension	Diabetes	Smoking（1=Yes，2=No）
1	Male	71	173	64.5	21.55	1	1	1	1	2	1
2	Female	72	155	85	35.38	1	1	3	1	1	2
3	Female	61	162	61.5	23.43	1	1	1	1	2	2
4	Female	85	155	58	24.14	1	1	2	1	1	1
5	Female	62	157	58	23.53	2	1	1	2	2	2
6	Male	46	172	84	28.39	1	1	3	1	2	1
7	Male	60	173	78	26.06	2	1	2	2	2	1
8	Female	58	162	80	30.48	2	1	3	1	1	2
9	Female	60	161	80	30.86	1	1	3	1	2	2
10	Female	61	158	50	20.03	2	2	1	2	2	2
11	Male	53	173	90	30.07	1	1	3	1	1	1
12	Male	37	180	120	37.04	1	1	3	1	1	1
13	Male	64	168	70	24.80	1	1	2	1	2	1
14	Male	56	176	66	21.31	2	2	1	1	2	2
15	Male	50	170	62	21.45	1	1	1	1	2	1
16	Male	57	162	70	26.67	2	2	2	1	1	1
17	Male	51	171	76	25.99	2	2	2	2	1	2
18	Female	61	150	63	28.00	1	1	3	1	2	2
19	Female	52	169	59	20.66	1	2	1	1	1	2
20	Female	82	145	50	23.78	2	2	1	2	1	2
21	Female	57	157	65	26.37	2	2	2	1	1	2
22	Male	56	173	100	33.41	1	2	3	1	1	1
23	Female	42	155	64	26.64	1	2	2	1	2	2
24	Male	54	165	75	27.55	1	2	2	1	1	2
25	Female	72	162	61	23.24	2	2	1	2	2	2
26	Female	81	155	67	27.89	1	2	2	1	1	2
27	Male	41	170	73	25.26	1	2	2	1	2	2
28	Female	52	163	64	24.09	1	2	2	1	2	2
29	Male	75	163	70	26.35	2	1	2	1	2	1
30	Male	66	172	69	23.32	1	1	1	1	2	2
31	Female	25	159	70	27.69	1	1	2	1	2	2
32	Male	79	170	87	30.10	1	1	3	1	2	2
33	Male	63	176	110	35.51	1	1	3	1	1	1
34	Male	74	175	82	26.78	1	1	2	1	2	1
35	Female	56	158	60	24.03	1	1	2	1	2	2
36	Female	80	160	50	19.53	2	1	1	2	2	1
37	Female	61	160	78	30.47	2	2	3	1	2	2
38	Male	62	170	73.5	25.43	1	1	2	1	1	1
39	Male	65	174	98	32.37	1	1	3	1	1	1

福昕PDF编辑器

40	Male	79	166	75	27.22	1	1	2	1	2	1
41	Male	70	172	73	24.68	2	2	2	2	2	1
42	Male	77	170	70	24.22	1	1	2	1	1	1
43	Female	72	157	66	26.78	1	1	2	1	2	2
44	Female	61	157	71	28.80	1	1	3	1	2	2
45	Female	78	160	45	17.58	2	2	1	2	2	2
46	Male	62	165	65	23.88	1	1	1	1	1	2
47	Male	55	176	85	27.44	2	1	2	2	2	1
48	Female	53	164	84	31.23	2	2	3	2	2	2
49	Male	67	156	70	28.76	2	2	3	2	1	2
50	Female	54	153	58	24.78	1	2	2	1	2	2
51	Male	68	178	93	29.35	1	1	3	1	2	1
52	Male	45	170	67	23.18	2	1	1	2	1	1
53	Male	61	178	86	27.14	2	1	2	1	1	1
54	Male	53	165	65	23.88	2	2	1	2	2	1
55	Female	71	171	101	34.54	1	1	3	1	2	2
56	Male	54	177	99	31.60	2	1	3	2	2	1
57	Male	65	164	65	24.17	2	2	2	2	1	1
58	Female	69	147	75	34.71	1	1	3	1	1	2
59	Male	63	170	78	26.99	2	2	2	2	2	2
60	Male	67	170	74.5	25.78	1	1	2	1	1	1
61	Female	69	162	59.5	22.67	1	2	1	1	2	2
62	Female	69	156	76	31.23	1	2	3	1	2	1
63	Male	57	171	80.5	27.53	1	2	2	1	1	1
64	Female	70	158	57	22.83	1	2	1	1	2	2
65	Male	59	168	74	26.22	2	1	2	2	2	2
66	Male	54	165	65	23.88	1	2	1	1	2	1
67	Female	73	164	81	30.12	2	2	3	1	1	2
68	Male	54	175	92	30.04	2	2	3	2	1	1
69	Male	46	172	83	28.06	1	2	3	1	2	1
70	Female	71	158	62	24.84	1	2	2	1	1	2
71	Male	51	168	73	25.86	1	2	2	1	2	1
72	Female	56	157	55.5	22.52	1	1	1	1	1	2
73	Male	68	165	60	22.04	1	1	1	1	1	1
74	Male	55	176	93	30.02	2	1	3	2	2	1
75	Male	48	170	75	25.95	2	2	2	2	1	1
76	Male	62	174	75	24.77	1	1	2	1	2	1
77	Male	49	172	86	29.07	1	1	3	1	1	1
78	Male	55	170	61	21.11	1	2	1	1	2	1
79	Male	23	173	77	25.73	2	1	2	2	2	1

80	Male	80	163	64.5	24.28	1	2	2	1	2	1
81	Female	71	163	61	22.96	1	1	1	1	2	2
82	Male	75	161	80	30.86	1	1	3	1	2	1
83	Male	48	173	80	26.73	2	2	2	1	2	1
84	Male	64	162	51	19.43	1	2	1	1	2	1
85	Female	68	163	78	29.36	1	2	3	1	2	2
86	Male	51	178	90	28.41	1	1	3	1	2	2
87	Male	64	182	106	32.00	1	2	3	1	1	1
88	Male	67	175	67	21.88	2	2	1	1	2	1
89	Female	61	155	65	27.06	1	2	2	1	2	2
90	Male	67	170	64	22.15	2	2	1	2	1	1
91	Male	63	173	68	22.72	2	2	1	2	1	1
92	Male	58	170	58	20.07	2	1	1	2	1	1
93	Female	57	163	62	23.34	1	2	1	1	2	2
94	Male	63	175	70	22.86	2	2	1	1	1	2
95	Female	72	162	70	26.67	1	1	2	1	2	2
96	Male	48	176	82	26.47	1	2	2	1	1	1
97	Male	72	175	98	32.00	2	2	3	2	1	1
98	Male	69	170	67	23.18	1	2	1	1	1	1
99	Male	56	163	92	34.63	1	1	3	1	2	1
100	Male	43	177	99	31.60	2	1	3	2	2	1
101	Female	67	165	64	23.51	2	2	1	2	2	2
102	Male	60	170	75.5	26.12	1	2	2	1	2	1
103	Female	33	166	55	19.96	2	2	1	1	1	2
104	Male	52	170	80	27.68	1	1	2	1	2	2
105	Male	75	172	78	26.37	1	2	2	1	2	1
106	Male	65	180	76	23.46	2	2	1	1	2	2
107	Male	75	161	61	23.53	2	2	1	2	2	2
108	Male	69	170	69	23.88	1	1	1	1	2	1
109	Male	64	170	83.2	28.79	1	1	3	1	1	1
110	Female	74	167	71	25.46	1	1	2	1	1	2
111	Male	67	164	74	27.51	1	2	2	1	2	1
112	Male	51	178	77	24.30	1	2	2	1	2	1
113	Male	85	170	68.5	23.70	1	1	1	1	2	2
114	Male	58	183	98	29.26	2	1	3	2	2	1
115	Male	62	175	65	21.22	1	1	1	1	1	2
116	Male	76	165	86	31.59	1	1	3	1	2	2
117	Female	71	150	42	18.67	1	2	1	1	2	2
118	Female	60	162	51	19.43	2	1	1	1	2	2
119	Male	64	164	68	25.28	1	2	2	1	1	1

120	Male	55	174	83	27.41	1	1	2	1	1	1
121	Male	63	170	98	33.91	1	1	3	1	2	1
122	Male	79	170	73	25.26	2	1	2	2	1	1
123	Male	54	175	82	26.78	2	2	2	2	1	2
124	Female	72	156	55	22.60	1	2	1	1	1	2
125	Female	71	167	61	21.87	1	2	1	1	1	2
126	Male	73	172	86	29.07	1	1	3	1	1	1
127	Female	73	170	70	24.22	1	2	2	1	2	2
128	Male	41	168	82	29.05	2	1	3	2	2	1
129	Male	69	172	72	24.34	1	1	2	1	2	1
130	Male	61	175	89	29.06	1	1	3	1	1	1
131	Male	60	170	89.5	30.97	1	1	3	1	1	1
132	Male	81	165	70	25.71	1	1	2	1	1	2
133	Male	77	163	73	27.48	1	2	2	1	2	2
134	Male	72	170	70	24.22	1	1	2	1	2	1
135	Female	65	157	64	25.96	1	1	2	1	1	2
136	Male	39	173	101	33.75	1	1	3	1	2	1
137	Male	60	170	76	26.30	2	1	2	2	2	1
138	Female	84	150	52	23.11	1	2	1	1	2	2
139	Male	64	176	80	25.83	2	2	2	2	2	2
140	Female	75	151	56.5	24.78	1	1	2	1	2	2
141	Male	56	170	64	22.15	2	2	1	2	2	1
142	Male	70	170	65	22.49	1	2	1	1	1	2
143	Female	59	159	73	28.88	2	2	3	1	2	2
144	Female	75	160	58	22.66	1	2	1	1	2	2
145	Female	81	155	67	27.89	1	2	2	1	1	2
146	Male	63	170	83	28.72	1	1	3	1	2	2
147	Female	72	162	65	24.77	1	1	2	1	1	2
148	Male	64	163.5	61.5	23.01	1	1	1	1	1	1
149	Male	75	171	88	30.09	1	2	3	1	2	2
150	Female	64	162	68	25.91	1	2	2	1	1	2
151	Male	68	174	75	24.77	1	2	2	1	2	1
152	Male	57	160	65	25.39	1	1	2	1	2	2
153	Male	58	168	80	28.34	1	1	3	1	1	1
154	Male	57	162	64	24.39	2	2	2	1	2	1
155	Male	55	177	67	21.39	1	2	1	1	2	1
156	Female	69	155	58	24.14	1	1	2	1	1	2
157	Male	57	163	73	27.48	1	1	2	1	1	1
158	Female	53	167	70	25.10	2	1	2	2	2	2
159	Male	67	175	80	26.12	1	2	2	1	1	2

160	Male	51	180	90	27.78	1	1	2	1	1	1
161	Male	45	178	82	25.88	1	1	2	1	2	1
162	Male	60	168	70	24.80	1	2	2	1	1	1
163	Male	59	173	89	29.74	1	2	3	1	1	1
164	Male	63	162	92	35.06	1	2	3	1	1	1
165	Male	65	170	78	26.99	1	1	2	1	1	1
166	Male	60	175	85	27.76	1	1	2	1	2	1
167	Female	54	160	62	24.22	2	2	2	2	1	2
168	Male	73	170	71	24.57	2	1	2	1	2	1
169	Female	69	158	61	24.44	1	2	2	1	1	2
170	Male	53	165	70	25.71	2	1	2	2	2	1
171	Male	49	172	78	26.37	2	1	2	2	2	1
172	Female	66	158	47	18.83	1	2	1	1	2	2
173	Male	62	170	80	27.68	1	2	2	1	1	1
174	Male	59	175	69	22.53	1	2	1	1	2	1
175	Male	62	174	90	29.73	1	1	3	1	1	1
176	Male	56	175	95	31.02	1	2	3	1	1	1
177	Male	39	165	77	28.28	1	2	3	1	2	1
178	Female	69	162	70	26.67	1	2	2	1	2	2
179	Male	46	170	71	24.57	1	2	2	1	2	2
180	Male	73	174	65	21.47	2	2	1	1	2	1
181	Male	54	178	86	27.14	1	1	2	1	1	1
182	Male	78	170	70	24.22	1	1	2	1	1	1
183	Female	71	155	62	25.81	2	2	2	1	2	2
184	Male	42	165	81	29.75	2	2	3	2	1	1
185	Female	67	153	58	24.78	1	2	2	1	1	2
186	Male	73	174	62	20.48	2	1	1	2	1	1
187	Male	61	166	52	18.87	2	1	1	2	2	1
188	Male	65	170	80	27.68	1	2	2	1	2	1
189	Male	71	160	70	27.34	2	2	2	1	2	2
190	Male	46	170	80	27.68	1	2	2	1	2	2
191	Male	61	165	57	20.94	1	1	1	1	2	1
192	Female	72	152	59	25.54	2	1	2	2	2	2
193	Female	59	156	63	25.89	2	2	2	2	2	2
194	Male	69	174	81	26.75	1	2	2	1	1	1
195	Male	49	169	70	24.51	2	2	2	2	2	1
196	Male	67	167	62	22.23	1	2	1	1	2	2
197	Male	50	173	80	26.73	2	1	2	2	1	1
198	Male	55	173	75	25.06	2	1	2	2	1	1
199	Male	71	166	67	24.31	1	1	2	1	2	2

200	Male	57	168	65	23.03	1	1	1	1	1	1
201	Female	70	163	60	22.58	1	2	1	1	2	2
202	Female	78	156	57	23.42	2	1	1	2	2	2
203	Male	54	176	65	20.98	1	2	1	1	2	1
204	Male	72	170	68	23.53	2	1	1	2	2	1
205	Female	74	167	64.5	23.13	1	2	1	1	1	2
206	Female	76	155	65	27.06	1	1	2	1	2	2
207	Female	79	160	63	24.61	1	2	2	1	2	2
208	Male	53	171	70	23.94	1	1	1	1	1	2
209	Female	47	162	79	30.10	1	2	3	1	1	2
210	Male	48	160	80	31.25	1	2	3	1	2	1
211	Female	66	148	45	20.54	1	2	1	1	2	1
212	Female	55	153.5	59	25.04	2	1	2	2	2	2
213	Male	76	178	84	26.51	1	1	2	1	2	1
214	Male	76	170	65	22.49	2	2	1	2	1	2
215	Male	56	173	63	21.05	2	1	1	2	1	1
216	Female	62	156	68.5	28.15	1	1	3	1	1	2
217	Female	60	170	84	29.07	1	2	3	1	2	2
218	Female	74	149	67	30.18	1	2	3	1	2	2
219	Female	77	153	76	32.47	1	2	3	1	1	2
220	Female	69	157	49	19.88	2	1	1	2	2	2
221	Male	66	168	70	24.80	1	1	2	1	1	2
222	Male	46	178	79	24.93	2	2	2	2	2	1
223	Female	62	163	60	22.58	2	1	1	2	2	2
224	Female	67	155	66	27.47	1	2	2	1	2	2
225	Female	55	158	51	20.43	2	1	1	2	1	2
226	Female	73	165	76	27.92	1	2	2	1	2	2
227	Female	88	155	50	20.81	1	1	1	1	2	2
228	Male	73	177	80	25.54	2	2	2	1	2	1
229	Male	80	158	68.5	27.44	1	2	2	1	2	1
230	Female	79	157	77	31.24	1	2	3	1	2	2
231	Male	84	157	54.5	22.11	1	2	1	1	2	2
232	Male	75	171	80	27.36	2	2	2	2	2	1
233	Male	52	175	80	26.12	2	2	2	2	2	1
234	Female	83	154	50	21.08	2	1	1	2	2	2
235	Female	57	168	100	35.43	1	2	3	1	2	2
236	Male	52	165	89	32.69	2	2	3	1	2	1
237	Male	59	169	65	22.76	2	2	1	2	2	2
238	Male	51	168	67	23.74	1	1	1	1	2	1
239	Male	65	171	72.5	24.79	2	2	2	2	2	1

240	Female	73	158	53.6	21.47	1	1	1	1	1	2
241	Female	62	160	72	28.13	1	2	3	1	2	2
242	Male	69	160	62	24.22	1	1	2	1	2	2
243	Male	72	165	59	21.67	1	2	1	1	1	1
244	Male	62	160	72	28.13	1	2	3	1	2	1
245	Female	68	158	78	31.24	1	1	3	1	1	2
246	Male	55	168	78	27.64	2	2	2	1	1	1
247	Male	66	169	69	24.16	2	1	2	2	2	1
248	Female	69	168	68	24.09	1	1	2	1	2	2
249	Male	55	172	76	25.69	1	2	2	1	1	1
250	Male	52	166	60	21.77	1	2	1	1	1	2
251	Female	61	162	71	27.05	2	2	2	2	1	2
252	Male	51	170	73	25.26	2	2	2	2	1	1
253	Male	60	169	78	27.31	1	1	2	1	1	1
254	Male	55	175	101	32.98	1	2	3	1	2	1
255	Male	49	169	75	26.26	1	1	2	1	2	1
256	Female	71	168	66	23.38	1	2	1	1	1	2
257	Female	58	165	75	27.55	2	2	2	2	2	2
258	Male	78	170	73	25.26	2	2	2	2	1	2
259	Male	74	175	67	21.88	2	1	1	2	2	2
260	Male	50	170	72	24.91	2	2	2	2	2	2
261	Female	61	154	62	26.14	1	2	2	1	2	2
262	Female	49	157	73	29.62	1	1	3	1	1	2
263	Female	45	165	70	25.71	2	1	2	1	2	2
264	Male	67	158	54	21.63	2	1	1	2	2	2
265	Male	59	172	86	29.07	2	2	3	1	2	1
266	Female	77	155	68	28.30	1	2	3	1	2	1
267	Male	57	174	86	28.41	2	1	3	2	1	1
268	Male	68	168	70	24.80	1	2	2	1	2	1
269	Male	42	172	91	30.76	1	1	3	1	2	1
270	Male	48	170	95	32.87	1	1	3	1	2	2
271	Female	69	153	55	23.50	2	1	1	2	2	2
272	Female	45	155	75	31.22	1	2	3	1	2	2
273	Female	61	149.5	44	19.69	1	2	1	1	2	2
274	Male	41	180	85	26.23	2	2	2	1	1	1
275	Female	62	160	60	23.44	2	2	1	1	2	2
276	Female	74	155	50	20.81	1	2	1	1	1	2
277	Male	55	174	69	22.79	1	1	1	1	2	2
278	Male	64	179	79	24.66	1	1	2	1	2	2
279	Male	50	170	83	28.72	1	1	3	1	2	1

280	Male	67	176	78	25.18	1	1	2	1	2	2
281	Male	53	175	75	24.49	1	1	2	1	2	1
282	Male	76	165	66	24.24	1	1	2	1	2	2
283	Male	51	178	92	29.04	1	1	3	1	1	1
284	Male	60	167	73.6	26.39	2	1	2	2	2	1
285	Female	56	159	58	22.94	1	1	1	1	2	2
286	Male	60	170	87	30.10	1	1	3	1	1	1
287	Female	81	160	48	18.75	1	2	1	1	2	1
288	Male	81	170	86	29.76	2	2	3	2	2	1
289	Male	55	170	95	32.87	1	1	3	1	2	1
290	Male	66	170	83	28.72	1	1	3	1	2	1
291	Female	67	168	75	26.57	1	1	2	1	1	1
292	Male	64	160	82.5	32.23	1	1	3	1	1	1
293	Male	60	171	70	23.94	2	1	1	2	2	1
294	Female	75	158	68	27.24	1	1	2	1	2	2
295	Male	68	166	65	23.59	1	1	1	1	2	1
296	Male	43	173	82	27.40	1	1	2	1	2	1
297	Male	60	170	65	22.49	1	1	1	1	1	1
298	Male	54	168	72	25.51	1	1	2	1	2	1
299	Male	62	178	75	23.67	2	2	1	1	2	2
300	Male	69	170	75	25.95	2	2	2	2	2	1
301	Male	56	182	98	29.59	1	2	3	1	2	2
302	Male	82	172	58	19.61	1	2	1	1	2	1
303	Female	66	160	70	27.34	1	1	2	1	1	2
304	Female	63	158	56	22.43	1	1	1	1	2	2
305	Female	54	164	70	26.03	2	2	2	2	1	2
306	Male	59	158	57	22.83	1	2	1	1	2	1
307	Male	55	164	70	26.03	1	1	2	1	2	2
308	Male	71	170	54	18.69	1	1	1	1	2	1
309	Male	63	170	78	26.99	2	2	2	1	2	1
310	Female	59	160	47.5	18.55	1	2	1	1	2	2
311	Male	53	176	90	29.05	2	2	3	2	1	1
312	Female	66	162	81	30.86	1	1	3	1	2	2
313	Female	63	160	79	30.86	1	1	3	1	1	2
314	Male	63	170	80	27.68	2	1	2	1	1	1
315	Female	69	157	70	28.40	1	1	3	1	2	2
316	Female	70	150	40	17.78	2	2	1	2	1	2
317	Male	64	167	68	24.38	2	1	2	2	1	1
318	Female	60	158	60	24.03	1	1	2	1	1	2
319	Female	77	150	50.5	22.44	1	1	1	1	2	2

320	Male	53	171	86	29.41	1	2	3	1	2	1
321	Male	65	182	102	30.79	2	2	3	2	2	1
322	Female	61	162	76	28.96	2	2	3	1	2	2
323	Male	55	167	89	31.91	1	1	3	1	1	1
324	Male	54	178	81	25.56	2	2	2	2	2	2
325	Female	62	163	56	21.08	2	1	1	2	2	2
326	Female	69	162	57	21.72	2	1	1	1	2	2
327	Female	72	169	71	24.86	1	1	2	1	1	2
328	Female	67	151.5	68.1	29.67	1	1	3	1	1	2
329	Male	49	174	74.5	24.61	2	2	2	1	2	1
330	Male	40	182	114	34.42	2	2	3	2	2	1
331	Female	62	152	70	30.30	1	1	3	1	2	2
332	Male	52	172	68	22.99	1	2	1	1	2	1
333	Female	55	160	65	25.39	2	2	2	2	1	2
334	Female	72	147	52.5	24.30	1	1	2	1	2	2
335	Female	72	159	68.7	27.17	1	1	2	1	1	2
336	Female	55	158	89	35.65	1	2	3	1	2	2
337	Female	62	155	71	29.55	2	1	3	1	1	2
338	Male	80	167	70	25.10	2	2	2	2	2	1
339	Female	65	158	64	25.64	2	2	2	2	1	2
340	Male	50	168	85	30.12	2	1	3	1	1	1
341	Female	51	163	75	28.23	1	2	3	1	2	2
342	Male	66	172	72	24.34	2	1	2	2	1	1
343	Male	72	175	80	26.12	1	2	2	1	2	1
344	Male	72	167	90	32.27	2	1	3	2	2	1
345	Female	67	165	72	26.45	1	2	2	1	1	2
346	Male	62	170	79	27.34	1	2	2	1	2	1
347	Female	73	164	66	24.54	1	1	2	1	2	2
348	Male	73	167	67.5	24.20	2	2	2	1	2	1
349	Male	71	170	75	25.95	1	1	2	1	2	1
350	Male	61	170	76	26.30	1	2	2	1	2	2
351	Male	49	173	95	31.74	2	1	3	1	2	2
352	Male	57	178	86	27.14	1	1	2	1	1	1
353	Female	63	163	71	26.72	1	1	2	1	1	2
354	Female	79	160	60	23.44	1	1	1	1	1	1
355	Male	61	171.5	89.3	30.36	2	1	3	2	2	1
356	Male	51	178	103	32.51	1	1	3	1	1	1
357	Male	51	174	87	28.74	1	1	3	1	1	1
358	Male	55	174	75	24.77	2	1	2	1	1	1
359	Female	72	160	60	23.44	1	1	1	1	2	2

360	Female	75	166	76	27.58	1	1	2	1	1	2
361	Male	58	162	58	22.10	1	2	1	1	1	1
362	Male	52	168	75	26.57	2	2	2	1	2	1
363	Male	63	168	80	28.34	1	1	3	1	2	1
364	Female	60	158	72	28.84	1	1	3	1	1	2
365	Male	69	168.1	68.1	24.10	2	2	2	1	2	1
366	Male	61	167	63.9	22.91	1	1	1	1	2	1
367	Female	64	164	70	26.03	1	2	2	1	2	2
368	Male	60	162	71	27.05	1	1	2	1	1	2
369	Female	76	155	70	29.14	1	1	3	1	1	2
370	Female	61	162	66	25.15	1	2	2	1	2	2
371	Male	80	163	70	26.35	1	2	2	1	2	2
372	Female	74	153	54	23.07	2	2	1	1	1	2
373	Male	56	173	63	21.05	1	2	1	1	1	2
374	Female	64	159	56	22.15	2	1	1	1	1	2
375	Male	48	175	94	30.69	1	2	3	1	2	1
376	Male	63	163	89	33.50	1	1	3	1	1	1
377	Male	64	173	65	21.72	1	1	1	1	2	1
378	Male	72	180	94	29.01	1	1	3	1	2	1
379	Female	69	156	57	23.42	1	1	1	1	2	2
380	Male	51	170	70	24.22	1	2	2	1	2	1
381	Female	80	158	55	22.03	1	1	1	1	2	2
382	Male	56	165	86	31.59	2	1	3	2	2	2
383	Male	60	172	83	28.06	1	1	3	1	2	1
384	Male	78	169	74	25.91	1	1	2	1	1	1
385	Female	57	157	66	26.78	1	2	2	1	2	2
386	Female	61	151	54	23.68	2	1	1	2	1	2
387	Male	63	178	75	23.67	1	2	1	1	2	2
388	Male	62	169	77.4	27.10	2	1	2	1	1	1
389	Female	64	162	65	24.77	1	1	2	1	2	2
390	Male	66	177	92	29.37	2	2	3	2	1	2
391	Male	57	173	60	20.05	2	1	1	1	2	1
392	Male	56	172	73	24.68	2	1	2	2	1	1
393	Male	45	172	66	22.31	1	1	1	1	2	1
394	Male	69	180	87	26.85	2	1	2	1	2	2
395	Female	59	158	60	24.03	1	1	2	1	2	2
396	Male	70	168	69	24.45	1	2	2	1	1	1
397	Male	53	176	87	28.09	2	2	3	2	2	1
398	Male	60	173	74.5	24.89	1	1	2	1	2	2
399	Male	58	165	79	29.02	1	1	3	1	1	1

400	Male	51	166	68	24.68	1	1	2	1	2	1
401	Female	76	151	58	25.44	1	1	2	1	2	2
402	Female	60	158	51.5	20.63	2	1	1	1	2	2
403	Female	64	160	55	21.48	2	2	1	2	2	2
404	Female	80	160	59	23.05	1	1	1	1	2	2
405	Female	57	165	87	31.96	1	1	3	1	1	2
406	Male	60	175	82	26.78	1	2	2	1	1	1
407	Male	76	170	86	29.76	2	2	3	1	2	2
408	Male	61	171	51	17.44	2	2	1	2	2	1
409	Male	80	165	83	30.49	2	1	3	2	1	1
410	Male	50	175	74	24.16	2	1	2	2	2	1
411	Male	71	164	57	21.19	1	1	1	1	1	2
412	Male	57	172	90	30.42	2	1	3	2	2	1
413	Male	54	168	89	31.53	2	2	3	2	2	1
414	Female	79	150	63	28.00	1	1	3	1	1	2
415	Female	66	155	59	24.56	2	1	2	2	2	1
416	Male	50	168	80	28.34	1	1	3	1	2	1
417	Male	72	172	82	27.72	1	2	2	1	1	1
418	Male	66	179	90	28.09	1	1	3	1	2	2
419	Male	65	170	71	24.57	2	1	2	2	2	1
420	Male	75	166	71	25.77	1	2	2	1	2	2
421	Female	51	160	75	29.30	1	1	3	1	1	2
422	Female	62	161	89	34.34	1	2	3	1	1	2
423	Male	66	184	87	25.70	1	1	2	1	2	1
424	Female	60	158	89	35.65	1	1	3	1	2	2
425	Male	63	170	67	23.18	1	2	1	1	2	1
426	Female	53	162	72	27.43	1	1	2	1	1	1
427	Male	49	168	78	27.64	1	1	2	1	1	1
428	Male	74	167	78	27.97	1	1	2	1	2	2
429	Male	59	170	75	25.95	2	2	2	2	2	1
430	Male	68	170	84	29.07	1	1	3	1	1	1
431	Female	57	153	62	26.49	1	1	2	1	1	2
432	Male	71	170	63	21.80	1	1	1	1	1	1
433	Male	85	168	75	26.57	2	2	2	2	2	1
434	Male	61	175	84.5	27.59	1	1	2	1	1	1
435	Male	46	169	68	23.81	2	2	1	2	2	1
436	Male	74	174	85	28.08	1	2	3	1	1	1
437	Male	55	178	74	23.36	1	2	1	1	2	1
438	Male	52	174	85	28.08	1	1	3	1	1	1
439	Male	63	179	86	26.84	1	1	2	1	1	1

440	Male	78	172	68	22.99	1	1	1	1	1	1
441	Male	63	165	70	25.71	2	2	2	2	1	1
442	Male	71	170	85	29.41	1	1	3	1	2	1
443	Female	70	160	69	26.95	1	1	2	1	2	2
444	Female	85	150	51.5	22.89	1	1	1	1	2	2
445	Male	77	160	67	26.17	1	1	2	1	1	1
446	Female	74	160	58	22.66	1	1	1	1	1	2
447	Female	48	155	72	29.97	2	2	3	2	2	2
448	Female	74	154	63	26.56	2	2	2	2	1	2
449	Male	71	170	62	21.45	1	1	1	1	1	2
450	Male	50	165	80	29.38	1	1	3	1	1	1
451	Female	65	166	55	19.96	1	1	1	1	2	2
452	Male	48	166	70	25.40	2	1	2	1	2	1
453	Male	58	174	95	31.38	1	1	3	1	2	1
454	Male	79	180	80	24.69	1	1	2	1	2	1
455	Male	62	184	91.5	27.03	1	1	2	1	2	1
456	Female	60	158	59	23.63	1	2	1	1	1	2
457	Male	64	170	85.5	29.58	1	1	3	1	2	1
458	Female	48	150	65	28.89	1	1	3	1	2	2
459	Male	56	171	80	27.36	1	1	2	1	2	1
460	Male	61	176	83	26.79	2	1	2	2	2	1
461	Male	76	170	80	27.68	2	2	2	2	2	1
462	Female	67	156	75	30.82	1	1	3	1	2	2
463	Male	59	170	80	27.68	2	1	2	2	2	2
464	Male	50	170	75	25.95	2	2	2	2	2	1
465	Male	75	160	75	29.30	1	1	3	1	1	2
466	Male	63	168	68	24.09	1	2	2	1	2	1
467	Male	58	173	84	28.07	1	2	3	1	1	1
468	Female	63	153	56	23.92	1	1	1	1	1	2
469	Male	70	167	55	19.72	1	2	1	1	2	2
470	Male	76	165	72	26.45	1	2	2	1	2	1
471	Female	61	160	55	21.48	2	1	1	2	1	2
472	Male	53	176	98	31.64	2	1	3	1	1	1
473	Male	63	183	98	29.26	1	1	3	1	1	1
474	Male	59	180	84	25.93	2	2	2	1	2	1
475	Female	62	164	63	23.42	2	1	1	2	2	2
476	Male	58	175	74	24.16	2	2	2	2	2	1
477	Female	53	164	75	27.89	1	2	2	1	2	2
478	Female	73	150	70	31.11	1	2	3	1	2	2
479	Male	39	165	91	33.43	2	1	3	1	1	1

480	Male	47	176	95	30.67	1	1	3	1	1	1
481	Female	61	163	75	28.23	1	2	3	1	1	2
482	Female	55	155	70	29.14	1	1	3	1	2	2
483	Female	61	163	77	28.98	1	1	3	1	1	2
484	Male	66	170	61.5	21.28	2	1	1	2	2	1
485	Female	64	150	52	23.11	2	1	1	1	1	2
486	Male	45	180	72	22.22	2	1	1	1	2	1
487	Female	60	158	52	20.83	2	2	1	2	2	2
488	Female	56	163	54	20.32	2	2	1	2	2	2
489	Male	45	173	126	42.10	1	2	3	1	2	2
490	Female	52	162	75	28.58	1	2	3	1	2	2
491	Male	55	174	78	25.76	1	2	2	1	2	1
492	Male	55	178	95	29.98	2	2	3	2	2	1
493	Male	61	166	73	26.49	1	1	2	1	1	1
494	Female	70	152	62	26.84	2	2	2	2	2	2
495	Female	64	155	74	30.80	1	2	3	1	1	2
496	Male	60	172	100	33.80	2	2	3	2	1	1
497	Female	56	165	65	23.88	1	1	1	1	2	2
498	Male	62	173	65	21.72	1	1	1	1	2	1
499	Female	51	158	86	34.45	1	2	3	1	2	2
500	Male	58	179	98	30.59	1	2	3	1	1	2
501	Female	73	169	67.5	23.63	1	1	1	1	1	2
502	Male	48	176	78	25.18	2	2	2	2	2	1
503	Female	56	155	58.5	24.35	2	2	2	2	2	2
504	Male	59	165	63	23.14	2	2	1	2	2	2
505	Male	55	174	74	24.44	2	2	2	2	1	1
506	Male	66	174	80	26.42	2	2	2	2	2	1
507	Female	65	162	56.5	21.53	1	2	1	1	1	1
508	Female	49	164	91	33.83	2	2	3	2	2	2
509	Female	73	153	76	32.47	1	2	3	1	2	2
510	Male	43	172	76	25.69	2	2	2	2	2	1
511	Female	72	156	56	23.01	1	2	1	1	1	2
512	Male	50	172	77	26.03	2	2	2	2	2	2
513	Female	59	156	67	27.53	1	2	2	1	1	2
514	Female	67	156	68	27.94	1	2	2	1	2	2
515	Female	60	159	70	27.69	2	2	2	2	2	2
516	Male	54	181	85	25.95	1	1	2	1	2	2
517	Male	43	179	100	31.21	2	2	3	1	2	1
518	Female	67	160	67	26.17	1	2	2	1	2	2
519	Female	76	150	56	24.89	1	2	2	1	2	2

520	Female	69	165	79.5	29.20	1	2	3	1	1	2
521	Female	62	160	61.5	24.02	1	2	2	1	2	1
522	Male	51	176	91.5	29.54	1	1	3	1	1	2
523	Male	52	178	78	24.62	1	2	2	1	2	1
524	Male	76	168	80	28.34	2	2	3	1	2	1
525	Female	63	164	70	26.03	2	1	2	2	2	2
526	Female	64	162	64	24.39	2	2	2	2	2	2
527	Female	64	160	62	24.22	1	2	2	1	2	2
528	Male	79	180	76	23.46	1	2	1	1	2	2
529	Male	62	170	87	30.10	2	2	3	1	2	1
530	Male	74	175	80	26.12	2	1	2	2	2	2
531	Male	55	180	96	29.63	2	2	3	2	2	1
532	Male	68	169	78.5	27.49	1	1	2	1	2	1
533	Female	75	150	63	28.00	1	2	3	1	1	2
534	Male	84	175	70	22.86	1	2	1	1	2	2
535	Female	60	166	70	25.40	1	1	2	1	2	2
536	Male	76	176	91.5	29.54	1	1	3	1	1	2
537	Male	66	170	81	28.03	1	1	3	1	2	1
538	Female	65	166	74.5	27.04	1	1	2	1	1	2
539	Female	56	169	69	24.16	1	1	2	1	2	2
540	Male	59	170	74	25.61	1	1	2	1	2	1
541	Female	78	156	59	24.24	1	2	2	1	2	2
542	Female	69	164	75	27.89	1	2	2	1	2	2
543	Female	81	152	82.5	35.71	1	2	3	1	1	2
544	Male	70	172	89.5	30.25	1	2	3	1	2	2
545	Male	64	171	75	25.65	1	1	2	1	1	2
546	Female	78	150	52	23.11	1	1	1	1	2	2
547	Male	66	168	72	25.51	2	2	2	2	2	2
548	Male	72	158	72	28.84	1	1	3	1	1	2
549	Female	66	155	60	24.97	1	2	2	1	2	2
550	Male	75	168	77	27.28	1	1	2	1	2	2
551	Male	58	170	66	22.84	1	2	1	1	2	2
552	Male	76	160	65	25.39	1	2	2	1	2	2
553	Male	67	173	105	35.08	1	1	3	1	1	2
554	Female	86	150	46.5	20.67	2	1	1	1	2	2
555	Male	64	174	73	24.11	1	1	2	1	2	1
556	Male	76	167	77	27.61	1	2	2	1	2	2
557	Male	76	178	70	22.09	2	1	1	2	1	2
558	Male	64	178	95	29.98	1	1	3	1	2	1
559	Male	54	171	69	23.60	2	2	1	1	2	1

560	Male	49	170	75	25.95	1	1	2	1	2	1
561	Male	49	175	84	27.43	1	2	2	1	1	1
562	Male	50	173	130	43.44	1	1	3	1	1	1
563	Male	80	170	62	21.45	1	2	1	1	2	1
564	Male	66	155	47	19.56	2	2	1	2	2	1
565	Male	78	167	70	25.10	1	1	2	1	1	1
566	Male	79	174	74.5	24.61	1	1	2	1	2	2
567	Male	65	164	80	29.74	1	2	3	1	2	1
568	Male	55	175	76	24.82	1	2	2	1	2	1
569	Male	79	163	68	25.59	2	1	2	1	2	2
570	Male	67	174	79	26.09	1	1	2	1	2	1
571	Male	65	172	72.5	24.51	2	2	2	1	2	2
572	Female	61	163	92	34.63	1	1	3	1	1	2
573	Male	80	170	80	27.68	1	1	2	1	2	1
574	Female	69	168	76	26.93	2	2	2	2	2	2
575	Female	63	159	69	27.29	2	1	2	2	2	2
576	Male	63	176	84	27.12	1	1	2	1	2	1
577	Female	77	157	60	24.34	1	2	2	1	1	2
578	Male	74	172	66	22.31	2	2	1	2	1	1
579	Female	69	158	78	31.24	2	2	3	2	2	2
580	Male	50	170	70	24.22	1	1	2	1	2	2
581	Female	57	163	69	25.97	1	1	2	1	2	2
582	Male	81	150	90	40.00	1	1	3	1	1	1
583	Male	73	172	74	25.01	1	2	2	1	2	2
584	Male	55	176	102	32.93	2	1	3	1	2	1
585	Male	78	172	83	28.06	1	1	3	1	1	2
586	Male	59	172	97	32.79	1	2	3	1	2	1
587	Female	63	160	58	22.66	1	1	1	1	2	2
588	Female	57	160	50	19.53	2	1	1	2	2	2
589	Male	52	165	71	26.08	2	1	2	2	2	1
590	Female	46	155	59	24.56	2	2	2	2	2	2
591	Female	68	152	62	26.84	2	2	2	2	2	2
592	Female	55	167	80	28.69	2	2	3	1	2	2
593	Male	37	177	103	32.88	2	1	3	2	2	1
594	Male	76	167	80	28.69	1	1	3	1	2	1
595	Female	78	160	60	23.44	1	1	1	1	2	2
596	Male	70	175	85	27.76	2	2	2	1	2	1
597	Male	53	166	70	25.40	1	1	2	1	1	1
598	Female	73	158	70	28.04	1	1	3	1	1	2
599	Male	69	175	75	24.49	1	1	2	1	2	2

600	Female	81	146	53	24.86	2	1	2	2	2	2
601	Male	68	170	81	28.03	1	1	3	1	2	2
602	Female	73	157	62	25.15	1	2	2	1	2	2
603	Male	58	178	70	22.09	2	1	1	2	2	1
604	Male	68	167	68	24.38	2	1	2	2	1	1
605	Female	60	165	75	27.55	1	2	2	1	1	1
606	Male	55	174	73	24.11	2	2	2	1	2	1
607	Male	56	170	81	28.03	2	2	3	1	1	1
608	Female	65	161	69	26.62	1	2	2	1	1	2
609	Female	69	160	56	21.88	2	2	1	2	1	2
610	Male	61	164	64	23.80	2	1	1	2	1	1
611	Male	72	171	83.5	28.56	2	2	3	1	2	2
612	Male	57	180	75	23.15	2	2	1	2	2	2
613	Female	66	150	54	24.00	2	1	2	2	2	1
614	Male	69	175	76	24.82	1	1	2	1	2	1
615	Female	71	154	50	21.08	1	1	1	1	2	2
616	Male	76	169	55	19.26	2	2	1	2	2	1
617	Male	82	170	65	22.49	1	1	1	1	2	1
618	Male	52	160	60	23.44	2	2	1	2	2	1
619	Female	75	158	60	24.03	1	1	2	1	1	2
620	Male	59	169	73	25.56	2	2	2	2	2	2
621	Male	56	170	65	22.49	2	2	1	2	2	1
622	Male	74	171	69	23.60	1	1	1	1	1	1
623	Female	55	152	56	24.24	1	1	2	1	1	2
624	Female	76	156	54	22.19	2	2	1	2	2	2
625	Male	60	175	75	24.49	1	2	2	1	2	1
626	Female	67	158	62	24.84	1	2	2	1	2	2
627	Male	71	170	73	25.26	2	2	2	2	2	1
628	Female	76	160	55	21.48	1	1	1	1	1	2
629	Male	78	178	71	22.41	1	2	1	1	2	2
630	Male	71	167	67	24.02	1	1	2	1	2	2
631	Male	48	173	85	28.40	2	1	3	2	1	1
632	Female	67	156	77	31.64	2	2	3	1	1	2
633	Male	41	160	63	24.61	2	2	2	2	2	1
634	Female	81	150	48	21.33	1	2	1	1	1	2
635	Male	63	174	80	26.42	1	1	2	1	2	2
636	Male	55	165	77	28.28	2	1	3	2	2	1
637	Male	54	165	52	19.10	1	2	1	1	2	1
638	Male	56	173	85	28.40	2	2	3	2	2	1
639	Female	68	160	70	27.34	1	2	2	1	2	2

640	Female	74	158	52	20.83	1	2	1	1	2	2
641	Female	63	160	57	22.27	1	2	1	1	1	2
642	Male	78	170	87	30.10	1	1	3	1	2	1
643	Male	47	170	78	26.99	2	2	2	2	2	1
644	Female	59	160	53	20.70	2	2	1	2	2	2
645	Male	57	178	86.5	27.30	1	2	2	1	2	1
646	Female	78	157	53.5	21.70	1	1	1	1	1	2
647	Male	65	174	83	27.41	2	2	2	1	2	1
648	Male	52	180	81	25.00	1	1	2	1	2	2
649	Female	54	152	61.5	26.62	1	1	2	1	2	2
650	Female	75	148	59	26.94	2	1	2	2	2	2
651	Male	63	169	57	19.96	1	1	1	1	2	1
652	Female	55	166	61	22.14	2	1	1	2	2	2
653	Male	62	178	83	26.20	1	2	2	1	2	1
654	Female	68	150	60	26.67	1	1	2	1	2	2
655	Female	80	156	78.5	32.26	1	1	3	1	2	2
656	Male	53	170	83	28.72	1	1	3	1	1	1
657	Female	64	164	65	24.17	1	1	2	1	1	2
658	Female	69	160	67	26.17	1	1	2	1	1	1
659	Female	63	160	71	27.73	1	1	2	1	1	2
660	Male	79	170	84	29.07	1	2	3	1	2	2
661	Male	75	173	69	23.05	2	2	1	1	2	1
662	Male	79	168	76	26.93	2	2	2	2	2	2
663	Male	70	165	76	27.92	1	2	2	1	2	1
664	Male	56	176	81	26.15	1	1	2	1	1	1
665	Male	70	168	80	28.34	1	1	3	1	2	1
666	Male	71	156	70	28.76	1	2	3	1	2	2
667	Male	72	166	70	25.40	2	2	2	2	1	2
668	Male	65	180	91	28.09	2	1	3	1	1	1
669	Male	80	170	73	25.26	1	1	2	1	1	2
670	Male	49	167	84.5	30.30	1	1	3	1	1	1
671	Male	75	170	63	21.80	2	1	1	2	2	2
672	Female	53	158	70	28.04	1	1	3	1	2	1
673	Female	57	150	61	27.11	1	2	2	1	2	2
674	Male	61	183	99	29.56	2	1	3	2	1	1
675	Male	68	169	80	28.01	2	1	3	2	1	1
676	Male	54	175	70	22.86	2	2	1	2	2	1
677	Male	50	168	83	29.41	2	2	3	1	2	2
678	Male	52	170	79	27.34	1	1	2	1	1	1
679	Female	78	167	76	27.25	2	1	2	2	2	2

680	Male	56	168	72	25.51	1	1	2	1	1	1
681	Female	64	150	50	22.22	2	2	1	2	2	2
682	Male	80	172	83	28.06	1	2	3	1	2	1
683	Male	57	170	70	24.22	2	1	2	2	1	1
684	Male	78	169	52	18.21	1	2	1	1	1	2
685	Female	65	157	75	30.43	1	1	3	1	2	2
686	Male	59	178	80	25.25	2	2	2	2	2	1
687	Male	85	174	62	20.48	2	2	1	2	2	1
688	Male	65	173	68	22.72	1	1	1	1	2	1
689	Male	61	165	80	29.38	2	2	3	2	2	1
690	Female	60	162	65	24.77	1	1	2	1	1	2
691	Female	68	168	104	36.85	1	2	3	1	2	2
692	Male	59	172	69	23.32	1	2	1	1	1	1
693	Male	75	170	59	20.42	2	2	1	2	2	2
694	Male	60	172	84	28.39	1	2	3	1	1	1
695	Male	50	179	88	27.46	1	1	2	1	2	1
696	Female	64	167	75	26.89	1	1	2	1	2	2
697	Male	70	166	80	29.03	1	2	3	1	2	2
698	Male	55	165	82	30.12	1	1	3	1	2	2
699	Male	65	170	84	29.07	1	2	3	1	2	1
700	Male	52	170	96	33.22	1	2	3	1	2	2
701	Male	59	170	62	21.45	1	2	1	1	2	1
702	Male	67	169	65	22.76	1	2	1	1	2	1
703	Female	62	158	69.5	27.84	1	1	2	1	1	2
704	Male	55	165	60	22.04	2	2	1	2	2	2
705	Female	62	160	65	25.39	1	1	2	1	2	2
706	Male	61	173	85	28.40	1	1	3	1	1	1
707	Male	41	162	84	32.01	2	1	3	1	2	1
708	Male	61	170	69	23.88	1	1	1	1	2	1
709	Male	51	160	68	26.56	1	2	2	1	2	1
710	Female	84	160	54	21.09	1	2	1	1	1	2
711	Female	46	160	79	30.86	1	2	3	1	2	2
712	Female	59	160	70	27.34	1	2	2	1	1	2
713	Male	62	165	58	21.30	1	1	1	1	1	1
714	Male	53	170	78	26.99	1	1	2	1	1	1
715	Male	47	170	75	25.95	1	2	2	1	2	1
716	Male	67	172	69	23.32	2	2	1	2	2	2
717	Female	62	160	54	21.09	1	1	1	1	2	2
718	Male	62	174	80	26.42	1	1	2	1	2	2
719	Female	71	157	69.5	28.20	2	2	3	1	2	2

720	Male	56	173	102	34.08	1	1	3	1	1	1
721	Male	73	165	63	23.14	2	1	1	1	2	2
722	Male	46	170	75	25.95	1	2	2	1	2	2
723	Male	78	170	75	25.95	2	2	2	2	1	2
724	Male	59	170	85	29.41	1	2	3	1	2	1
725	Male	61	173	71	23.72	2	1	1	2	2	1
726	Male	39	177	77	24.58	1	2	2	1	2	1
727	Female	73	160	65	25.39	1	1	2	1	2	2
728	Male	56	172	84	28.39	1	2	3	1	2	1
729	Male	74	158	69	27.64	2	2	2	1	2	1
730	Female	67	155	60	24.97	1	2	2	1	2	2
731	Male	57	178	96	30.30	1	2	3	1	2	2
732	Female	73	158	60	24.03	1	2	2	1	2	2
733	Male	85	167	77	27.61	1	2	2	1	2	2
734	Female	71	152	69	29.86	1	1	3	1	1	2
735	Female	60	148	54	24.65	1	2	2	1	2	2
736	Male	69	180	75	23.15	1	1	1	1	2	1
737	Male	58	160	64	25.00	2	2	2	2	2	1
738	Male	61	169	75	26.26	2	2	2	1	2	2
739	Female	60	160	77	30.08	1	1	3	1	1	2
740	Female	74	153	56	23.92	1	2	1	1	2	2
741	Female	62	163	65	24.46	2	2	2	2	2	2
742	Male	61	188	101	28.58	1	2	3	1	2	1
743	Male	77	170	59	20.42	2	2	1	1	2	1
744	Male	52	170	100	34.60	1	1	3	1	1	1
745	Female	67	153	65	27.77	2	1	2	2	1	2
746	Male	71	177	86	27.45	1	1	2	1	1	1
747	Male	55	172	70	23.66	1	1	1	1	2	1
748	Male	74	170	73	25.26	2	2	2	2	2	1
749	Male	44	170	66	22.84	2	1	1	1	2	2
750	Male	54	170	84	29.07	2	2	3	1	2	1
751	Female	64	154	70	29.52	1	1	3	1	1	2
752	Female	54	156	66	27.12	2	2	2	2	2	2
753	Male	69	176	76.5	24.70	2	1	2	2	2	2
754	Female	73	160	67	26.17	1	1	2	1	1	2
755	Female	61	157	65	26.37	2	2	2	2	2	2
756	Female	74	160	91	35.55	1	1	3	1	2	2
757	Female	54	155	78	32.47	2	1	3	2	2	2
758	Male	58	165.5	75	27.38	1	2	2	1	2	1
759	Male	67	175	59	19.27	1	1	1	1	2	2

760	Male	56	166	79.5	28.85	1	1	3	1	2	1
761	Male	62	177.5	87.4	27.74	2	1	2	2	2	1
762	Male	50	170	87	30.10	1	1	3	1	2	1
763	Male	54	169	58	20.31	1	1	1	1	1	1
764	Male	60	175	121	39.51	1	2	3	1	2	2
765	Male	56	168	66.2	23.46	1	1	1	1	2	2
766	Female	68	152.5	72.5	31.17	1	2	3	1	1	2
767	Female	69	157.5	70.9	28.58	2	2	3	2	2	2
768	Female	75	159	57	22.55	2	1	1	1	2	2
769	Female	75	163	80	30.11	1	1	3	1	2	1
770	Female	62	156	59	24.24	2	2	2	1	1	2
771	Male	62	169	84	29.41	1	1	3	1	1	1
772	Female	82	150	56	24.89	1	1	2	1	2	2
773	Male	72	167	64	22.95	1	2	1	1	2	2
774	Male	55	164.5	68.5	25.31	1	1	2	1	1	1
775	Male	59	157	69.3	28.11	1	2	3	1	2	2
776	Female	77	157	71	28.80	1	1	3	1	2	2
777	Male	66	173	102	34.08	1	1	3	1	1	1
778	Male	59	177	97	30.96	1	1	3	1	1	1
779	Female	47	160	83.2	32.50	1	1	3	1	1	2
780	Male	72	166.5	74.6	26.91	1	1	2	1	2	2
781	Male	57	163	67.9	25.56	1	1	2	1	2	1
782	Male	64	170	56	19.38	2	2	1	1	1	1
783	Female	80	149	54.5	24.55	1	2	2	1	1	1
784	Female	71	166	49	17.78	2	2	1	2	2	2
785	Female	51	167	77	27.61	1	1	2	1	1	2
786	Female	78	158.5	55.6	22.13	1	1	1	1	2	2
787	Male	50	173	80	26.73	1	2	2	1	2	1
788	Female	62	158.5	60.1	23.92	2	2	1	2	1	2
789	Female	75	152	70	30.30	1	1	3	1	1	2
790	Male	81	173	71	23.72	1	1	1	1	1	1
791	Male	60	159	59	23.34	2	1	1	2	2	1
792	Male	61	167.5	73	26.02	1	1	2	1	2	1
793	Female	76	151.5	64.1	27.93	1	2	2	1	2	2
794	Female	80	152	71.6	30.99	1	1	3	1	2	2
795	Male	66	171.5	80.5	27.37	1	1	2	1	1	2
796	Female	69	162	60	22.86	2	2	1	2	2	2
797	Female	64	154.5	78.6	32.93	2	2	3	1	2	2
798	Male	57	170.5	85	29.24	2	2	3	1	2	1
799	Male	54	171.5	82	27.88	1	2	2	1	1	1

800	Male	76	166	76.9	27.91	1	1	2	1	2	1
801	Male	56	173	84.8	28.33	2	1	3	2	1	2
802	Female	59	149.5	54	24.16	2	2	2	2	2	2
803	Female	70	155.5	69.2	28.62	1	2	3	1	2	2
804	Female	72	151.5	51.3	22.35	1	2	1	1	2	2
805	Female	72	155.5	72.5	29.98	1	1	3	1	1	1
806	Male	53	177.5	81	25.71	2	2	2	2	2	1
807	Male	58	166	67.3	24.42	2	1	2	1	2	2
808	Male	51	178	82.8	26.13	1	1	2	1	2	1
809	Male	42	174	91.1	30.09	2	2	3	2	2	1
810	Male	84	157.5	71.4	28.78	2	1	3	2	1	2
811	Male	58	172	72.5	24.51	2	2	2	2	2	1
812	Male	58	172	70	23.66	1	1	1	1	2	1
813	Male	44	165	80.1	29.42	2	2	3	1	1	1
814	Female	78	152.5	62.5	26.87	1	1	2	1	2	2
815	Male	57	180	76	23.46	2	1	1	2	2	1
816	Female	59	166	69.4	25.19	2	1	2	2	2	2
817	Male	74	166.5	79.7	28.75	1	1	3	1	1	1
818	Male	57	161	62.6	24.15	2	1	2	2	2	1
819	Male	56	169.5	73	25.41	2	2	2	2	2	1
820	Male	66	169.5	72.9	25.37	1	1	2	1	2	2
821	Male	46	173	94	31.41	1	1	3	1	2	1
822	Female	71	148	55.5	25.34	1	2	2	1	1	2
823	Male	55	166	79	28.67	1	1	3	1	2	1
824	Female	42	158	61.8	24.76	2	1	2	2	2	2
825	Male	59	162.5	74	28.02	2	1	3	1	1	1
826	Female	80	160	69.8	27.27	1	1	2	1	2	2
827	Male	71	166	67.8	24.60	2	1	2	2	2	2
828	Male	72	163	56.3	21.19	2	2	1	1	2	1
829	Male	79	170	82.7	28.62	2	1	3	2	1	2
830	Male	77	170	66.3	22.94	1	1	1	1	1	2
831	Male	77	171	77	26.33	1	2	2	1	2	1
832	Male	76	160	77	30.08	1	2	3	1	2	1
833	Male	78	174	76.5	25.27	2	2	2	2	2	2
834	Male	56	168	72	25.51	2	2	2	2	2	1
835	Male	76	174	79	26.09	1	2	2	1	1	2
836	Male	71	170	71.5	24.74	1	1	2	1	1	2
837	Female	69	160	65	25.39	1	2	2	1	1	2
838	Male	44	178	84.9	26.80	2	1	2	2	2	1
839	Male	60	181.5	84.68	25.71	1	2	2	1	2	1

840	Female	80	160	65	25.39	1	1	2	1	2	2
841	Female	80	155	61	25.39	1	2	2	1	2	2
842	Male	72	158	63.5	25.44	1	2	2	1	2	1
843	Male	60	172	62	20.96	1	2	1	1	1	1
844	Male	85	168	70	24.80	2	2	2	2	2	2
845	Male	61	165	70.6	25.93	1	1	2	1	2	2
846	Female	62	160	73	28.52	1	2	3	1	2	2
847	Male	69	164.5	79.5	29.38	1	1	3	1	1	1
848	Female	75	157	53.6	21.75	1	1	1	1	2	2
849	Male	79	166	65.4	23.73	1	1	1	1	2	2
850	Female	72	155	61.2	25.47	1	1	2	1	2	2
851	Female	45	164.5	71.5	26.42	1	2	2	1	2	2
852	Female	65	164	61.8	22.98	2	1	1	2	1	2
853	Male	45	176	87.1	28.12	1	1	3	1	2	1
854	Male	47	174	64.1	21.17	1	2	1	1	1	1
855	Male	69	165	70	25.71	2	1	2	2	2	1
856	Female	63	151	58.7	25.74	1	2	2	1	2	2
857	Female	77	159	69	27.29	2	1	2	2	2	2
858	Female	58	167	73.4	26.32	2	2	2	2	2	2
859	Male	69	165	75	27.55	2	1	2	2	2	1
860	Male	55	174	77.2	25.50	1	2	2	1	1	1
861	Male	77	176.5	60.7	19.48	2	1	1	2	2	1
862	Female	58	160.5	73.7	28.61	1	1	3	1	2	2
863	Female	55	164	69	25.65	2	2	2	2	1	2
864	Female	74	161.5	61	23.39	1	2	1	1	1	2
865	Male	53	174.5	103.9	34.12	2	2	3	2	2	1
866	Female	81	156	55	22.60	1	1	1	1	1	2
867	Female	54	164	76	28.26	1	2	3	1	2	2
868	Female	60	161	61	23.53	1	2	1	1	2	2
869	Male	60	165	59.2	21.74	2	1	1	2	2	2
870	Female	66	149	58.1	26.17	2	2	2	2	2	2
871	Female	57	162.5	68.4	25.90	2	2	2	2	2	1
872	Female	74	161	56.2	21.68	1	1	1	1	2	2
873	Female	77	162	80	30.48	2	2	3	1	2	1
874	Female	80	156.5	54.5	22.25	2	2	1	2	2	2
875	Male	65	165	69.9	25.67	1	1	2	1	2	1
876	Male	53	176	106	34.22	1	1	3	1	2	2
877	Male	51	165	65	23.88	2	1	1	2	2	2
878	Male	49	185	82	23.96	2	2	1	2	2	2
879	Female	74	158	55	22.03	1	1	1	1	1	2

880	Female	65	159.5	77	30.27	2	2	3	1	1	2
881	Female	66	154	67.8	28.59	1	1	3	1	2	2
882	Female	56	164	70	26.03	1	1	2	1	1	1
883	Male	62	172	74.5	25.18	2	1	2	2	2	1
884	Female	57	160	58	22.66	2	1	1	1	2	2
885	Male	63	176	90	29.05	1	1	3	1	1	1
886	Male	52	174	79.1	26.13	1	1	2	1	2	2
887	Male	71	157	58.1	23.57	1	2	1	1	2	2
888	Male	46	173.5	84	27.90	1	2	2	1	2	2
889	Male	60	172.5	78.5	26.38	2	2	2	1	1	1
890	Female	78	154	74	31.20	2	1	3	2	2	2
891	Male	67	173	100	33.41	1	2	3	1	2	2
892	Male	80	168	84.7	30.01	1	1	3	1	1	1
893	Male	63	160.5	63	24.46	2	1	2	2	1	1
894	Female	59	161.5	65.1	24.96	1	2	2	1	1	2
895	Female	74	161	54	20.83	1	2	1	1	2	2
896	Female	59	150.5	60.9	26.89	1	2	2	1	1	2
897	Male	57	167	73.4	26.32	1	1	2	2	2	1
898	Female	53	163.5	76	28.43	1	1	3	1	2	1
899	Male	62	171	71	24.28	1	1	2	1	1	1
900	Female	77	153	63.5	27.13	1	2	2	1	2	2
901	Female	78	157	76.3	30.95	1	1	3	1	1	1
902	Male	69	163	78.4	29.51	1	1	3	1	2	2
903	Male	68	170	57.1	19.76	2	1	1	2	2	2
904	Female	57	157	64	25.96	2	1	2	2	2	1
905	Female	56	153	63	26.91	1	2	2	1	1	2
906	Male	71	168.5	82.3	28.99	1	1	3	1	2	1
907	Male	56	169	85.6	29.97	1	1	3	1	2	1
908	Female	70	153.5	56.5	23.98	1	1	1	1	1	2
909	Female	79	159	79.1	31.29	1	1	3	1	1	2
910	Male	60	171	85	29.07	1	1	3	1	1	1
911	Male	58	180	75	23.15	2	2	1	2	2	1
912	Male	72	168	70	24.80	1	1	2	1	2	2
913	Female	69	159	60	23.73	2	1	1	2	2	2
914	Male	53	163	61.7	23.22	1	1	1	1	1	1
915	Male	60	154	88	37.11	1	1	3	1	2	2
916	Male	64	165.5	65	23.73	2	2	1	1	1	1
917	Female	76	161	67.2	25.92	2	1	2	1	2	2
918	Male	60	167.5	68	24.24	1	1	2	1	1	1
919	Male	62	174	78	25.76	1	1	2	1	1	2

920	Male	81	168	67	23.74	2	1	1	1	2	2
921	Male	69	174	75	24.77	2	1	2	2	1	1
922	Female	69	159	78	30.85	1	1	3	1	1	2
923	Female	44	161	91.7	35.38	2	2	3	1	2	2
924	Male	82	170	71	24.57	1	2	2	1	1	1
925	Male	57	183	81.9	24.46	1	1	2	1	2	1
926	Male	53	166	66	23.95	1	1	1	1	1	1
927	Female	55	160	60.5	23.63	1	2	1	1	2	2
928	Male	57	177	97	30.96	2	2	3	1	1	1
929	Female	52	169.5	80.5	28.02	1	1	3	1	2	2
930	Female	75	165	80.4	29.53	1	1	3	1	1	2
931	Female	70	156	70.5	28.97	1	2	3	1	1	2
932	Male	52	180.5	78	23.94	2	1	1	2	2	1
933	Female	78	152.5	64	27.52	2	1	2	2	1	1
934	Male	75	166	74.1	26.89	2	1	2	2	2	2
935	Male	50	171	91	31.12	1	1	3	1	2	1
936	Male	58	174	76	25.10	2	2	2	2	1	2
937	Female	68	165	63.4	23.29	2	1	1	1	1	2
938	Male	68	170	64.4	22.28	1	2	1	2	1	1
939	Male	55	178.5	85.5	26.83	1	1	2	1	2	1
940	Male	62	185.5	95.2	27.67	1	1	2	1	1	1
941	Male	59	178	87.7	27.68	1	1	2	1	1	1
942	Male	52	183	85	25.38	1	2	2	1	2	2
943	Male	53	176	69.5	22.44	2	2	1	1	1	1
944	Female	71	160	58.3	22.77	2	2	1	1	2	2
945	Male	64	172.5	84.5	28.40	1	1	3	1	2	1
946	Female	72	151.5	56.7	24.70	1	2	2	1	2	2
947	Female	51	166	65	23.59	1	1	1	1	2	2
948	Female	68	158	73	29.24	2	2	3	2	2	2
949	Female	63	162.5	66	24.99	1	1	2	1	2	2
950	Female	67	160	60	23.44	2	2	1	2	2	2
951	Female	65	157	55.5	22.52	1	1	1	1	2	2
952	Female	66	164	73.3	27.25	1	2	2	1	2	2
953	Female	65	166.5	55	19.84	1	1	1	1	2	2
954	Female	65	155.5	57.4	23.74	1	1	1	1	1	2
955	Male	69	167	94.1	33.74	1	2	3	1	1	1
956	Female	65	156.5	78	31.85	1	1	3	1	1	1
957	Male	55	174.1	50.8	16.76	1	2	1	1	1	1
958	Male	54	171	86.5	29.58	1	1	3	1	2	1
959	Female	68	160	64.8	25.31	2	2	2	2	2	2

960	Female	68	146.5	72.3	33.69	2	1	3	1	2	2
961	Female	76	148	52.3	23.88	1	1	1	1	1	2
962	Male	54	174	88.1	29.10	1	2	3	1	2	1
963	Male	61	173.5	72	23.92	1	1	1	1	1	1
964	Female	62	163	83	31.24	1	1	3	1	1	2
965	Male	66	182	87	26.26	1	2	2	1	1	2
966	Male	52	171.5	74.3	25.26	2	2	2	2	1	2
967	Male	54	175.5	104.8	34.03	1	1	3	1	2	2
968	Female	56	165	66	24.24	1	2	2	1	2	2
969	Male	57	167.5	74.5	26.55	1	2	2	1	2	2
970	Female	75	155	57.5	23.93	2	1	1	2	2	2
971	Female	72	152	75	32.46	1	1	3	1	1	2
972	Male	73	168	63	22.32	2	1	1	1	2	1
973	Female	59	155	77	32.05	2	1	3	2	2	2
974	Female	75	155	78	32.47	1	2	3	1	2	2
975	Male	80	174	82	27.08	1	2	2	1	2	1
976	Male	57	170	90	31.14	2	2	3	2	2	1
977	Female	72	158.5	62	24.68	2	1	2	2	2	2
978	Male	56	172.5	75	25.20	1	1	2	1	2	1
979	Male	61	177	81	25.85	2	2	2	2	1	2
980	Male	66	174	82	27.08	2	2	2	2	2	1
981	Male	70	175	76.4	24.95	2	1	2	2	1	2
982	Male	59	172	82.6	27.92	1	2	2	1	2	1
983	Male	52	168.5	66.3	23.35	2	2	1	2	2	1
984	Female	81	154.5	48.6	20.36	1	2	1	1	2	2
985	Male	52	180	78	24.07	1	1	2	1	1	2
986	Female	62	152.5	72.3	31.09	1	1	3	1	2	2
987	Male	61	172	91.6	30.96	2	1	3	2	2	1
988	Male	83	161	58.3	22.49	1	1	1	1	2	2
989	Female	54	158.5	57.6	22.93	1	2	1	1	2	2
990	Male	74	169	85.7	30.01	1	1	3	1	1	1
991	Male	71	162.2	92.2	35.05	1	1	3	1	1	1
992	Male	45	171	75	25.65	2	1	2	1	2	1
993	Male	74	175.5	80.2	26.04	2	1	2	2	1	1
994	Female	72	157	61.5	24.95	2	2	2	2	1	2
995	Male	63	167.5	77.6	27.66	1	2	2	1	2	2
996	Male	52	182	97.4	29.40	2	2	3	2	2	2
997	Female	62	157	71.5	29.01	1	2	3	1	1	2
998	Male	57	174	96.2	31.77	1	1	3	1	2	2
999	Female	47	161.5	93	35.66	1	1	3	1	2	2

1000	Female	78	148.5	53.8	24.40	1	1	2	1	2	2
1001	Male	63	173.5	81.9	27.21	1	1	2	1	1	1
1002	Male	55	180	96.2	29.69	1	2	3	1	1	1
1003	Male	59	166	64.1	23.26	2	2	1	2	2	1
1004	Male	50	190.5	89.8	24.74	2	2	2	1	1	1
1005	Female	64	150	80.4	35.73	1	1	3	1	1	2
1006	Male	64	168.5	82.8	29.16	2	2	3	2	2	2
1007	Male	67	177	80.4	25.66	1	1	2	1	1	1
1008	Male	64	168	77	27.28	1	1	2	1	1	2
1009	Male	64	166	82	29.76	1	2	3	1	2	2
1010	Male	59	175	74	24.16	1	1	2	1	1	1
1011	Female	67	157	62.4	25.32	1	1	2	1	2	2
1012	Female	67	150	69	30.67	2	1	3	2	2	1
1013	Male	79	166	55.2	20.03	2	1	1	1	2	1
1014	Male	72	163	70.6	26.57	1	1	2	1	1	1
1015	Male	49	175.5	73	23.70	1	2	1	1	2	1
1016	Female	67	157	72.9	29.58	1	2	3	1	2	2
1017	Female	49	169.5	78.1	27.18	1	1	2	1	2	2
1018	Male	64	162	73.1	27.85	1	1	2	1	2	1
1019	Female	68	150	71.6	31.82	1	2	3	1	1	2
1020	Male	51	179	92	28.71	1	1	3	1	2	1
1021	Male	48	167	85	30.48	2	1	3	2	2	1
1022	Male	64	170	78	26.99	2	1	2	2	1	1

Number	TG	TC	HDL-C	LDL-C	ABI R	ABI L	PWV R	PWV L	R systolic	R diatolic	L systolic	L diatolic Bp
1	1.02	4.74	1.44	2.86	1.26	1.28	1338	1440	120	64	119	64
2	1.55	3.86	0.78	2.54	1.45	1.27	2278	2301	155	80	150	70
3	1.32	3.78	1.31	1.75	1.05	1.08	1293	1263	127	65	126	67
4	6.29	3.81	0.45	1.61	1.1	1.15	1913	1971	132	67	133	66
5	1.85	4.89	1.03	3.2	1.17	1.21	1694	2071	118	64	118	63
6	3.97	3.89	0.88	2.12	1.04	1.16	1264	1181	109	72	115	75
7	2.82	4.02	0.97	2.19	1.14	1.22	1822	1784	121	78	129	79
8	1.24	3.26	0.78	2.03	1.05	1.15	1489	1492	140	81	144	81
9	2.49	5.4	1.14	2.95	1.05	1.02	1527	1395	120	71	132	76
10	1.33	5.04	3.28	3.28	1.14	1.07	1416	1446	95	61	91	50
11	1.59	3.15	0.77	1.86	1.22	1.11	1636	1723	148	88	154	85
12	2.41	3.46	0.8	2.02	1.2	1.23	1376	1434	133	83	125	84
13	3.06	3.86	1.1	3.02	1.27	1.24	1893	2083	129	79	129	80
14	0.89	5.32	1.8	3.14	1.17	1.14	1344	1329	113	70	113	66
15	2.36	4.46	1.48	2.47	1.11	1.1	1146	1104	142	83	145	81
16	1.91	3.69	0.79	1.97	1.18	1.16	1553	1505	154	76	152	76
17	1.17	4.78	1.15	3.52	1.23	1.19	1290	1281	109	65	109	68
18	0.55	3.36	1.31	1.65	1.13	1.07	1382	1536	114	61	110	59
19	1.06	3.98	1.39	1.86	1.13	1.14	1214	1247	113	83	116	81
20	1.04	4.56	1.16	2.99	1.28	1.21	1641	1747	115	59	115	59
21	2.19	4.5	0.8	3.07	1.11	1.08	1477	1496	133	81	142	79
22	0.99	4.52	3.32	3.32	1.28	1.23	1308	1327	137	71	136	74
23	3.81	3.65	0.96	1.81	1.05	1.03	1496	1475	124	84	128	86
24	1.06	3.14	1.86	1.25	1.18	1.21	1516	1602	136	83	130	80
25	1.89	5.58	1.32	2.78	1.18	1.17	1432	1468	128	70	129	67
26	7.6	6.35	0.83	3.96	1.15	1.09	1696	1805	143	71	133	70
27	4.05	3.54	0.87	1.97	1.28	1.22	1254	1307	116	70	118	74
28	1.96	5.77	1.06	4.27	1.24	1.24	1499	1467	122	80	122	81
29	1.22	3	0.82	1.73	1.14	1.16	1146	1151	144	82	141	80
30	1.1	3.61	1.21	1.96	1.12	1.13	1558	1615	139	84	136	84
31	7.47	4.84	0.71	1.59	1.06	1.05	896	925	125	72	117	63
32	0.69	2.56	0.96	1.4	1.24	1.22	1558	1823	126	62	121	59
33	1.5	5.15	1.36	4.16	1.12	1.08	1279	1307	132	81	125	73
34	0.95	3.2	0.78	2.13	1.08	1.17	1768	1753	133	76	125	78
35	1.49	4.52	1.17	2.75	1.04	1.06	1731	1814	157	75	151	74
36	1.48	4.15	0.95	2.74	1.17	1.17	2208	2257	138	66	137	65
37	2.76	6.23	0.77	1.46	0.99	1.01	1569	1591	137	83	143	85

38	3.06	5.33	1.02	2.61	1.04	1.16	1509	1499	129	84	135	89
39	2.7	6.63	1.18	4.23	1.17	1.09	2129	2480	129	63	125	65
40	1.26	4.55	1.05	3.06	1.18	1.08	1269	1378	131	69	127	65
41	1.59	3.92	0.7	2.53	1.26	1.26	1759	1685	133	72	135	74
42	0.8	3.03	0.75	1.97	1.05	1.01	1735	1785	144	78	152	79
43	2.24	3.44	0.94	1.75	1.08	1.23	1591	1735	143	82	141	81
44	3.49	4.19	1.04	2.17	1.22	1.15	1583	1593	154	90	158	91
45	0.55	3.99	1.23	2.51	1.13	1.2	1657	1564	122	88	116	84
46	1.69	4.57	0.89	3.06	1.15	1.17	1646	1607	110	88	110	76
47	1.86	3.24	0.65	1.82	1.19	1.12	1094	1189	113	69	113	73
48	1.11	4.07	1.25	3.32	1.16	1.26	1305	1403	127	58	122	58
49	2.16	3.7	1.16	1.66	1.22	1.1	1801	1744	102	63	106	62
50	1.53	4.54	1	2.92	1.04	1.1	2438	2465	189	111	184	114
51	1.89	2.38	0.62	1.19	1.23	1.13	1327	1412	154	82	153	85
52	3.39	3.39	0.83	1.78	1.22	1.22	1238	1247	106	59	108	65
53	2.1	3.77	0.76	2.4	1.16	1.21	1425	1354	112	65	112	69
54	0.57	2.85	1.01	1.57	1.15	1.1	1332	1367	124	80	122	78
55	1.04	2.74	0.82	1.46	1.2	1.28	1453	1526	160	85	153	82
56	3.16	5.02	0.86	3.45	1.16	1.11	1139	1108	113	71	117	74
57	11.19	4.49	0.63	1.23	1.14	1.07	1890	1868	122	81	119	82
58	2.63	4.06	0.88	2.18	1.16	1.16	1753	1850	144	80	141	80
59	1.55	3.56	0.81	2.02	1.11	1.17	1530	1384	120	80	122	70
60	4.7	4.09	0.86	1.91	0.64	0.64	1175	970	175	101	180	96
61	0.91	3.89	1.33	2.05	0.98	0.98	1403	1388	115	70	115	88
62	2.32	5.85	0.81	2.93	1.13	1.05	1386	1408	128	70	130	71
63	0.95	2.98	0.89	1.83	1.18	1.1	1683	1545	143	88	146	88
64	2.52	4.21	0.95	2.01	1.16	1.19	2038	1944	126	77	139	88
65	1.72	5.62	0.91	4.02	1.1	1.14	1685	1672	108	74	115	81
66	1.79	4.5	1.02	2.79	1.27	1.2	1492	1522	127	76	128	75
67	1.37	4.26	1	2.55	1.05	1.1	1761	1744	147	74	137	83
68	2.99	3.3	0.67	1.47	1.18	1.18	1320	1264	103	55	106	68
69	1.28	4.79	0.77	3.49	1.2	1.28	1693	1715	128	87	125	87
70	2.01	2.77	0.62	1.25	0.92	1.02	1852	1814	119	59	111	60
71	1.14	3.36	0.97	1.96	1.2	1.21	1402	1396	121	80	121	81
72	0.89	5.72	1.59	3.73	1.07	1.15	1235	1281	122	69	122	68
73	1.93	3.32	0.78	2.12	0.96	0.62	1972	1978	124	97	130	78
74	1.92	3.77	1.02	2.1	1.17	1.14	1196	1250	106	75	108	77
75	3.06	4.15	1.06	1.37	1.19	1.34	1355	1375	118	72	122	71

76	1.02	4.04	0.96	2.63	0.72	0.94	1154	1167	108	68	100	68
77	2.12	2.57	0.73	1.02	1.2	1.2	1276	1277	116	80	112	79
78	2.25	4.25	0.77	2.5	1.15	1.03	1246	1233	119	78	118	79
79	2.52	2.62	1.42	1.42	1.07	1.18	993	943	100	52	108	55
80	1.09	4.97	0.98	3.64	1.12	1.16	1717	1675	151	87	151	88
81	1.11	3.78	1.35	1.56	1.19	1.17	1431	1477	116	54	111	54
82	1.95	4.15	2.12	2.12	0.86	0.77	1470	1458	131	79	132	83
83	0.81	4.27	0.84	3.05	1.12	1.08	1661	1564	138	88	145	95
84	0.77	4.08	0.88	2.69	1.21	1.2	1384	1443	110	66	110	70
85	1.2	3.82	0.98	1.78	1.16	1.23	2194	2249	116	57	120	61
86	2.3	4.31	0.86	2.61	1.33	1.23	1329	1320	124	79	119	75
87	0.62	3.42	0.91	1.54	1.13	1.01	1423	1409	123	86	141	86
88	3.2	4.98	0.93	2.18	1.21	1.18	1468	1514	132	76	136	78
89	3.82	6.1	1.04	3.2	1.19	1.15	1664	1680	145	79	153	82
90	1.3	4.64	1.14	3.34	1.19	1.12	1400	1362	99	55	107	63
91	2.01	4.7	1.03	2.97	1.16	1.15	2370	2314	119	65	121	67
92	0.82	3.17	0.8	1.91	1.15	1.13	1289	1353	106	67	110	57
93	0.85	3.57	1.03	2.87	1.05	1.05	1421	1322	111	69	116	75
94	1.29	4.05	0.71	2.85	1.17	1.21	1813	1869	154	83	147	83
95	1.42	3.4	0.99	1.96	1.18	1.19	1436	1450	122	65	119	93
96	1.03	4.42	0.88	2.24	1.16	1.17	1345	1342	114	71	110	72
97	1.46	4.27	0.8	2.67	1.28	1.3	1407	1577	131	75	135	78
98	1.12	3.51	1	2.02	0.91	0.9	2005	1809	148	82	142	78
99	1.39	3.47	0.8	2.04	1	1.01	1603	1729	128	74	123	76
100	5.41	5.41	0.69	2.35	1.14	1.13	1248	1234	134	75	139	80
101	1.04	4.75	1.01	1.3	1.03	1.04	1409	1444	109	56	115	61
102	3.2	2.91	0.5	1.39	1.23	1.22	1472	1490	113	72	113	71
103	1.92	2.9	0.59	2.12	1.01	0.99	1184	1161	98	61	99	61
104	2.21	4.94	1.09	2.7	1.09	1.2	2002	1974	159	92	160	95
105	1.7	3.93	0.52	2.61	1.07	1.13	1652	1557	113	61	126	65
106	1.06	4.35	0.96	2.67	1.23	1.29	1692	1701	145	89	148	91
107	0.7	3.5	1.24	1.83	1.19	1.27	1544	1571	138	76	136	74
108	1.86	3.38	0.91	1.86	1.08	1.08	1561	1552	113	76	119	78
109	1.98	3.21	0.79	1.71	1.23	1.29	1771	1801	132	76	130	78
110	1.56	3.08	1.04	1.43	1.07	1.13	1703	1896	118	84	127	57
111	0.83	3.05	1.01	1.77	1.18	1.21	1969	2084	145	78	143	77
112	2.48	4.36	1.3	2.12	1.24	1.2	1718	1679	156	95	158	99
113	2.31	3.35	1.11	1.73	1.19	1.15	2246	2156	144	72	141	72

114	2.27	3.57	0.8	1.78	1.14	1.2	1247	1282	129	84	131	78
115	0.69	3.87	1.28	2.02	1.19	1.17	1470	1516	123	65	122	67
116	0.87	3.9	1.48	2.06	1.15	1.18	1753	1795	122	64	123	58
117	6.82	5.82	1.46	2.61	1.19	1.23	1880	1881	124	71	122	73
118	1.88	5.55	0.84	3.75	1.13	1.18	1421	1489	118	74	118	75
119	1.22	3.92	0.67	2.67	1.14	1.21	2301	2405	144	82	142	85
120	1.76	4.83	0.95	3.31	0.98	0.71	1945	1151	155	89	158	84
121	1.04	2.71	0.75	1.55	1.09	1.19	1520	1745	122	72	137	80
122	1.52	4.4	0.95	2.78	1.03	1.15	1757	1669	110	63	106	62
123	1.17	4.48	0.88	3.52	1.14	1.24	967	938	103	62	104	61
124	1.22	5.35	1.29	3.59	1.16	1.15	1669	1575	127	84	130	84
125	4.82	5.53	1.04	2.3	1.06	1.02	1708	1876	160	84	163	87
126	1.71	3.88	0.66	2.57	1.08	1.1	1807	1797	152	76	153	77
127	1.61	3.94	0.76	2.38	1.17	1.25	1924	1944	152	84	150	90
128	1.51	4.28	0.83	2.82	1.02	1.08	1399	1395	120	77	115	76
129	0.97	2.53	0.77	1.75	1.24	1.23	1656	1615	123	72	123	72
130	1.74	2.81	0.91	1.48	1.03	1.08	1332	1328	98	67	101	70
131	1.92	2.45	0.87	0.99	1.14	1.1	1640	1433	135	77	133	77
132	1.1	2.95	0.93	1.55	1.1	1.13	1954	2017	155	75	158	70
133	2.55	3.83	0.76	2.03	1.16	0.96	1482	1645	110	67	113	63
134	2.29	3.83	1.02	1.78	1.2	1.19	2052	2054	155	81	148	82
135	2.01	3.71	0.98	2.05	1.08	1.18	1818	1951	121	80	122	91
136	1.96	7.18	0.66	5.41	1.15	1.19	1617	1635	145	87	142	86
137	1.03	4.26	1.16	2.74	1.1	1.09	1268	1317	113	74	117	74
138	1.82	5.07	0.96	2.21	0.95	0.88	2254	2284	147	63	153	66
139	1.13	2.69	0.91	1.28	1.22	1.17	1388	1356	118	71	117	71
140	1.85	3.97	1.16	2.19	1.15	1.17	1634	1639	149	80	144	79
141	1.55	3.2	0.74	2.09	1.14	1.15	1618	1637	120	73	123	71
142	2.31	6.33	0.88	3.25	1.01	0.99	1863	1811	146	73	145	74
143	1.91	3.44	0.75	1.93	1.13	1.16	1623	1592	135	78	131	77
144	0.64	4.7	1.34	1.91	0.99	1.04	1778	1992	127	63	134	62
145	1.34	5.04	1.08	3.5	0.61	1.08	575	1548	118	55	124	63
146	1.37	3.89	1.07	2.13	1.14	1.2	1580	1655	133	82	138	87
147	0.83	2.91	1.1	1.46	1.15	1.23	1790	1774	140	77	142	76
148	0.84	2.88	0.96	1.03	1.06	1.13	2261	2433	179	98	180	97
149	1.41	4.83	1.15	3.39	1.28	1.3	1849	2064	151	88	150	86
150	1.33	3.59	1.48	1.75	0.97	1.13	1359	1299	127	66	120	59
151	0.86	3.88	1	2.36	1.25	1.29	1098	1092	105	66	106	66

152	0.65	3.66	1.22	1.86	0.95	1.16	1778	1940	150	82	151	84
153	2.22	4.85	0.77	1.72	1.11	1.09	2193	2377	135	81	130	78
154	4.11	6.49	0.9	3.53	1.13	1.12	1831	1882	150	92	147	87
155	3.47	5.1	0.99	2.83	1.27	1.2	1180	1177	114	64	121	66
156	1.37	3.4	1.06	1.83	1.09	1.1	1739	1920	143	72	143	74
157	1.13	2.77	0.86	1.47	1.23	1.21	1663	1695	155	71	159	77
158	3.93	3.93	1.12	2.35	1.12	1.09	1268	1234	100	61	99	71
159	1.58	4.07	0.68	2.96	1.09	1.01	1674	1586	138	72	144	71
160	0.76	2.31	0.75	1.27	1.22	1.17	1267	1173	123	73	127	68
161	1.46	3.2	0.87	1.9	1.17	1.19	1630	1684	138	89	138	90
162	2.23	5.44	1.12	3.59	0.91	1.11	1437	1368	118	74	129	81
163	1.18	4	0.77	2.73	1.13	1.2	1290	1372	127	72	126	74
164	2.13	5.79	1.19	3.66	0.83	1.08	1500	1626	168	87	168	88
165	3.27	3.27	0.91	2.06	0.93	0.99	1521	1554	134	76	131	75
166	0.53	2.97	0.88	1.79	1.06	1.14	1305	1201	114	68	119	73
167	1.1	3.46	1.14	2.03	1.23	1.2	1630	1642	132	71	129	70
168	0.74	2.91	1	1.43	1.21	1.2	1881	1676	107	65	106	65
169	0.91	3.58	0.98	2.33	1.19	1.11	1952	1794	122	73	118	67
170	1	3.21	1.11	1.58	1.14	1.06	1415	1359	125	68	119	66
171	1.82	3.67	0.79	2.4	1.21	1.23	1064	1054	101	61	103	61
172	0.8	2.79	1.1	1.8	1.08	1.22	2088	2119	132	79	137	81
173	2.11	4.49	1.02	2.96	1.07	1.13	1516	1532	136	81	125	74
174	1.78	3.69	0.06	2.24	1.13	1.03	1566	1780	124	68	135	73
175	1.87	3.68	1.06	2.02	1.09	1.18	1480	1466	143	76	150	75
176	2.39	3.81	0.83	2.27	1.12	1.25	1372	1395	172	82	193	86
177	4.86	6.48	0.98	3.89	1.16	1.14	1921	1881	153	92	151	96
178	1.69	4.57	1	2.68	1.17	1.23	1513	1639	130	68	139	71
179	1.48	4.5	1.28	2.59	1.15	1.11	1403	1522	114	72	110	77
180	1.15	5.43	1.46	3.55	1.1	1.12	1952	1891	160	79	162	80
181	2.91	3.5	0.71	1.56	1.23	1.19	1676	1675	151	86	151	87
182	5.86	4.4	0.6	1.13	1.13	1.2	1842	1791	135	80	136	79
183	1.58	3.85	0.93	2.05	1.12	1.1	2263	2177	147	75	141	73
184	4.62	5.64	0.7	3.25	0.88	0.96	1211	1235	128	71	117	69
185	2.42	3.08	0.69	1.81	1.13	1.12	1745	1866	159	84	159	81
186	0.8	2.47	0.85	1.16	1.16	1.21	1936	1898	134	75	131	72
187	1.24	2.92	0.77	1.86	0.94	1.03	1561	1661	116	70	119	68
188	1.91	4.64	0.88	2.69	1.08	1.23	1632	1636	130	80	129	86
189	1.96	4.32	0.99	2.55	1.22	1.19	1816	1812	141	93	143	88

190	1.37	4.31	0.71	2.96	1.22	1.22	1661	1641	144	84	140	85
191	1.27	4.71	1.15	3.02	0.85	1.13	1345	1785	130	77	136	80
192	0.88	6.49	1.29	5.01	0.78	0.85	1135	1159	116	65	113	66
193	1.48	5.74	0.97	4.26	1.08	1.05	1133	1166	92	48	95	63
194	0.87	3.63	0.85	2.52	1.02	1.04	1528	1558	124	75	123	72
195	2.98	2.98	0.89	1.8	1.13	1.2	1374	1368	112	68	112	66
196	2.75	2.75	0.79	1.47	1.15	1.13	2804	2362	198	111	202	111
197	1.11	4.08	0.91	2.79	1.15	1.13	1470	1486	137	87	134	82
198	2.87	2.68	0.47	1.36	0.71	1.13	818	1434	115	73	115	76
199	0.64	3.77	1.22	2.37	1.12	1.19	1522	1538	144	81	143	77
200	1.74	4.14	0.96	2.62	1.21	1.28	1465	1589	117	71	113	67
201	1.35	4.25	0.89	2.57	1.21	1.25	1763	1802	153	90	152	90
202	1.32	3.13	0.99	1.59	1.16	1.23	1737	1850	129	62	120	58
203	1.17	3.38	0.79	2.14	1.21	1.19	1124	1108	116	81	114	82
204	0.49	4.04	1.43	2.23	1.2	1.22	1541	1550	130	73	130	74
205	4.44	4.44	1.04	2.78	1.16	1.17	1345	1336	118	67	120	65
206	0.75	2.77	0.98	1.56	1.04	1.17	1294	1372	130	69	131	68
207	0.85	3.89	1.05	2.45	1.01	1.07	2038	1990	142	60	141	60
208	1.14	2.97	0.8	1.91	1.15	1.15	1617	1670	138	86	145	87
209	1.48	3.98	0.73	2.71	1.08	1.17	1492	1540	145	91	143	92
210	1.89	4.58	1.08	3.02	1.18	1.14	1045	981	105	71	107	68
211	0.59	3.12	0.94	1.82	1.22	1.24	1881	1946	143	91	143	79
212	1.03	4.55	1.2	2.98	1.2	1.12	1304	1346	102	68	103	65
213	0.67	3.13	1.13	1.65	0.97	0.97	1366	1523	128	69	126	68
214	0.72	3.4	0.85	2.31	1.28	1.11	1993	1829	111	60	112	63
215	0.73	3.4	1.02	2	1.23	1.21	1617	1652	128	71	126	71
216	2.89	4.44	1.27	2.29	1.16	1.16	1504	1479	135	75	135	75
217	1.06	4.9	1.12	3.2	1.25	1.24	1494	1606	133	71	127	66
218	1.24	4.1	1.22	2.3	1.2	1.23	1493	1438	132	54	129	58
219	0.92	3.48	1.3	1.71	0.99	0.91	2120	2002	138	63	122	61
220	0.98	2.94	0.97	1.51	1.11	1.15	1412	1458	130	70	122	63
221	2.03	3.84	1.1	2.06	0.75	1.04	1157	1175	119	67	121	68
222	1.44	4.21	0.57	3.16	1.13	1.1	1481	1568	127	75	132	76
223	1.41	3.4	0.96	1.93	1.06	1.02	1970	1794	132	70	131	74
224	1.6	3.61	1.04	2.02	1.13	1.32	1427	1335	126	70	122	69
225	1.03	4	1.12	2.16	1.14	1.13	1297	1308	98	59	105	60
226	1.38	5.83	1.81	3.37	1.15	1.19	1532	1420	118	61	117	61
227	2.46	4.61	1.44	2.27	1.06	1.07	1938	1764	131	63	96	58

228	1.76	4.4	0.88	2.98	1.08	1.06	1541	1537	157	87	156	89
229	1.35	4.28	0.93	3.07	1.03	1.07	1716	1769	144	70	137	68
230	1.26	2.95	0.75	2.28	1.17	1.1	1692	1860	132	67	131	66
231	0.64	2.96	0.96	1.58	1.12	1.15	1926	1927	130	87	128	86
232	1.78	3.1	0.79	1.87	1.18	1.26	1457	1477	109	67	107	63
233	1.64	5.24	1.15	2.97	1.12	1.11	1371	1406	123	79	119	78
234	2.85	3.79	1.3	1.98	1.16	1.13	1551	1428	111	63	113	63
235	2.04	4.87	1.18	2.88	1.18	1.13	1105	1049	120	65	118	62
236	3.72	5.53	0.9	3.38	1.13	1.15	1373	1351	120	75	115	70
237	1.14	3.56	0.95	2.02	1.11	1.08	1306	1291	99	61	101	59
238	1.13	3.57	1.05	1.83	1.15	1.1	1423	1442	134	89	133	91
239	0.73	4.93	1.17	3.38	1.23	1.22	1487	1496	115	78	113	79
240	1	2.56	0.72	1.35	1.08	1.03	2525	2433	144	80	145	81
241	2.28	4.76	0.94	2.92	1.04	1.04	1852	1831	140	71	148	73
242	2.22	4.27	0.9	2.5	1.02	1.04	1205	1261	118	86	121	66
243	1.33	4.4	0.9	2.81	0.64	0.7	1481	1457	115	59	109	54
244	2.67	5.85	0.91	3.31	1.2	1.13	1496	1456	119	78	123	74
245	0.63	3.57	1.23	1.92	1.19	1.22	1478	1414	116	65	125	71
246	3.81	3.4	1.01	1.03	1.09	1.1	1847	1936	149	95	146	92
247	0.74	3.09	0.97	1.86	1.28	1.27	1416	1334	116	61	122	65
248	1.23	4.1	1.38	1.64	1.22	1.15	1533	1702	113	66	111	63
249	1.57	3.17	0.79	1.54	1.08	1.07	1462	1424	121	84	122	79
250	0.72	2.9	0.94	1.5	1.12	1.1	1426	1425	129	74	129	80
251	2.15	4.11	1.2	2.03	1.08	1.1	1532	1746	122	85	131	74
252	3.26	4.74	0.81	2.86	1	0.8	1499	1320	122	73	124	75
253	1.77	3.05	0.82	1.97	1.13	1.05	1573	1626	126	75	125	77
254	3.95	5.56	0.72	3.2	1.15	1.25	1541	1518	125	84	133	88
255	2.1	5.67	0.88	3.73	1.08	1.1	1467	1418	128	84	129	87
256	1.1	4.84	0.9	3.23	1.02	1.11	1434	1519	130	71	132	72
257	5.49	5.3	0.82	2.55	1.26	1.24	1285	1260	123	78	118	75
258	1.13	3.45	0.78	2.29	1.1	1.04	1582	1516	117	70	119	70
259	1.29	3.61	0.79	2.21	1.17	1.22	2206	2166	138	76	138	78
260	1.82	2.95	0.87	1.07	1.05	1.09	1174	1210	104	67	101	61
261	1.09	4.45	0.08	3.18	1.28	1.27	1991	1994	146	77	141	72
262	1.17	4.12	1.08	2.49	1.14	1.21	1319	1303	119	74	125	75
263	2.27	4.42	1.02	2.69	1.09	1.11	1559	1484	140	84	138	77
264	0.67	2.85	0.78	3.8	1.11	0.91	1217	1365	91	55	92	61
265	1	4.06	0.79	2.73	1.17	1.07	1476	1514	142	86	144	86

266	2.83	6.3	0.74	4.57	0.92	0.85	1660	1494	110	67	110	65
267	2.46	3.17	0.53	1.86	1.33	1.12	1195	947	108	62	106	62
268	1.27	4.56	1.35	2.81	1.17	1.25	1752	1640	135	87	136	85
269	1.36	3.02	0.94	1.64	1.16	1.21	1081	1077	116	64	115	66
270	1.99	3.81	0.93	2.15	1.08	1.1	1214	1191	114	72	125	73
271	0.72	4.35	1.04	2.94	1	0.97	2322	2422	120	89	119	83
272	2.39	3.74	0.67	2.41	0.99	1.08	1006	1028	93	60	91	61
273	4.48	4.48	1.49	2.72	1.1	1.12	2188	2402	130	83	127	83
274	2.44	6.86	1.08	4.42	0.98	1.01	1436	1453	127	82	137	83
275	2.11	5.39	0.98	3.64	1.14	1.19	1832	2019	153	82	155	83
276	0.81	5.92	1.6	3.76	1.01	0.98	1316	1318	116	65	115	67
277	1.31	3.82	0.9	2.51	1.23	1.2	1761	1758	135	86	131	88
278	2.28	3.68	1.02	1.94	1.19	1.19	1776	1931	124	83	124	85
279	2.22	3.33	0.78	1.91	1.18	1.17	1554	1592	132	90	131	87
280	0.72	2.63	0.79	1.46	1.14	1.13	1290	1347	118	81	117	76
281	1.21	2	0.82	1.38	1.35	1.36	1301	1283	125	75	125	70
282	2.22	4.41	0.65	2.87	1.08	1.07	1904	1645	100	70	125	73
283	1.39	2.57	0.71	1.28	1.22	1.2	1312	1374	118	73	117	73
284	0.88	2.81	0.92	1.49	1.08	1.07	1324	1281	105	67	110	67
285	2.12	7.16	1.26	5.12	1.07	1.1	1664	1677	118	65	114	62
286	1.03	3.5	1.2	1.84	1.06	0.99	1462	1411	138	77	130	71
287	1	3.93	1.06	2.4	1.08	1.03	1881	2018	113	66	110	63
288	1.83	4.6	0.95	2.93	1.12	1.14	1652	1724	129	76	137	77
289	1.17	3.5	0.79	2.35	1.22	1.15	1446	1397	126	80	131	84
290	0.94	3.35	1.05	1.85	1.3	1.24	1376	1488	131	83	130	80
291	2.04	4.93	1.09	2.79	1.09	1.16	1346	1389	141	97	141	92
292	1.77	4.6	1.03	3.06	1.13	1.27	1772	1825	123	70	121	69
293	2.74	3.51	1.08	1.67	1.03	1.03	1569	1528	111	64	116	66
294	1.31	2.97	0.94	1.65	1.08	1.14	2020	2072	137	57	145	56
295	0.51	3.86	1.14	2.38	1.14	1.2	1641	1611	138	71	136	66
296	0.99	2.25	0.6	1.17	1.07	1.11	1202	1162	88	57	87	56
297	2.34	3.75	0.96	1.96	1.01	1.09	1390	1467	124	67	100	60
298	0.68	2.6	0.96	1.45	1.21	1.18	1086	1165	119	79	117	70
299	2.14	4.2	0.54	2.65	1.27	1.22	1488	1510	140	86	142	87
300	1.08	4.3	1	2.75	1.21	1.06	1315	1310	127	72	112	66
301	1.96	4.13	1.06	2.64	0.98	1	1466	1491	124	80	129	82
302	0.97	3.4	1.01	1.94	1.22	1.15	1847	1772	139	72	144	71
303	3.46	5.09	1.03	2.15	1.17	1.18	1650	1720	135	79	133	83

304	4.83	4.83	0.85	3.1	1.17	1.15	1178	1238	108	57	102	55
305	2.02	5.26	0.87	3.21	1.13	1.08	1101	1088	105	57	107	58
306	0.46	4.93	1.35	1.79	1.13	1.12	1414	1379	147	89	149	87
307	2.44	4.65	0.98	2.8	1.14	1.15	2051	1943	145	88	144	87
308	0.7	3.59	1.21	2.03	1.13	1.23	1400	1434	120	64	117	63
309	1.08	5.63	1.07	3.88	1.09	1.16	1281	1248	150	85	147	85
310	1.08	5.8	1.45	3.76	1.08	1.08	1662	1677	132	79	131	81
311	4	3.1	0.59	2.39	1.38	1.37	1321	1311	112	73	112	65
312	1.44	5.88	0.87	1.15	0.98	1.09	1563	1616	152	83	171	97
313	1.33	5.05	1	3.46	1.18	1.17	1930	1887	149	76	155	79
314	2.42	4.36	0.81	2.66	1.13	1.09	1825	1851	130	78	130	83
315	0.84	3.94	1.66	1.67	1.2	1.19	1969	2044	151	82	156	85
316	0.46	4.44	1.69	2.29	1.03	1.12	1740	1987	139	75	138	75
317	1.33	3.98	1.01	2.36	1.05	1.04	1508	1495	105	70	103	63
318	2.4	3.67	0.9	2.09	1.16	1.16	1664	1680	120	69	122	74
319	0.79	3.06	1.39	1.36	1.1	1.16	1839	2001	135	60	130	59
320	3.93	3.93	0.86	2.63	1.15	1.17	1403	1422	131	81	132	86
321	0.69	4.21	1.02	2.75	1.13	1.23	1239	1264	115	68	114	67
322	3.71	5.01	1.32	3.13	1.1	1.15	1274	1320	123	71	121	73
323	1.59	3.74	0.79	2.24	1.15	1.12	1479	1503	116	78	117	81
324	1.01	4.21	0.94	2.93	1.23	1.16	1394	1416	124	79	123	76
325	3.65	4.83	0.98	2.41	1.21	1.17	1400	1421	107	64	104	58
326	2.59	5	1.25	2.41	1.26	1.2	2162	2297	133	75	132	75
327	0.73	5.61	2.18	2.37	1.2	1.06	1783	1908	122	66	114	56
328	1.9	3.65	1.17	1.5	1.14	1.15	1628	1597	116	69	123	69
329	3.29	4.54	1.04	2.25	1.16	1.21	1525	1492	141	87	145	87
330	5	5.62	1.27	2.17	1.03	1.09	1242	1233	127	83	126	81
331	0.73	2.9	0.74	1.91	1.17	1.18	1478	1606	121	67	100	57
332	1.96	3.1	0.91	1.88	1.16	1.13	1485	1496	123	85	125	83
333	1.63	4.25	1.42	2.25	1.21	1.17	1277	1276	104	60	102	64
334	0.76	3.91	1.35	1.69	1.07	1.21	2825	2007	131	60	126	69
335	1.47	3.7	1.54	1.5	1.21	1.15	1465	1511	125	76	125	76
336	1.55	4.9	1.25	2.9	1.03	1.06	1669	1745	142	78	144	80
337	2.08	4.91	0.85	3.75	1.15	1.08	1708	1677	147	86	147	87
338	1.26	4.41	1.09	4.28	1.13	1.06	4398	2553	126	77	127	77
339	1.38	3	1.02	1.41	1.05	1.06	1538	1440	116	62	119	66
340	8.7	6.97	0.9	2.12	1.12	1.34	1556	1507	129	88	130	90
341	2.35	2.88	0.99	1.27	1.11	1.2	1717	1740	117	71	123	74

342	1.54	4.31	2.94	2.94	1.13	1.04	1681	1610	130	79	133	80
343	5.15	4.32	0.8	1.57	0.71	0.75	1050	1170	109	69	117	70
344	1.38	3.33	0.82	1.96	1.09	1.12	1778	1819	137	79	135	78
345	1.97	3.67	1.03	1.92	1.07	1.09	1961	2002	171	92	173	92
346	2.2	2.97	0.87	1.87	1.13	1.06	1584	1657	143	79	141	79
347	2.31	7.48	1.11	5.02	1.05	0.96	2031	2029	143	68	138	67
348	0.92	4.82	1.38	2.97	1.03	1.03	1511	1600	144	76	138	76
349	1.24	3.02	0.69	1.75	1.07	1.11	1631	1666	109	68	107	73
350	1.87	4.37	0.75	2.8	1.11	1.13	1530	1575	120	78	118	80
351	5.31	4.89	0.98	2.03	1.38	1.4	1368	1351	129	75	133	78
352	0.9	3.28	0.81	2.24	1.23	1.23	1341	1242	119	71	120	69
353	5.89	3.33	0.65	0.9	1.12	1.1	1333	1259	130	68	125	66
354	3.17	4.64	0.89	2	0.99	1.16	1544	1735	132	63	138	64
355	1.48	4.48	1.14	2.72	1.2	1.2	1942	1962	138	74	139	75
356	3.17	3.69	0.84	1.8	0.88	0.93	1061	1040	115	70	125	78
357	2.96	3.21	0.63	1.6	1.09	1.13	1177	1123	121	71	120	73
358	0.83	3.67	0.9	2.22	1.14	1.14	1254	1235	116	65	119	66
359	2.67	3.93	1.09	2.73	1.09	1.12	1784	1786	118	79	123	79
360	1.11	3.19	1.04	1.64	1	1.04	1472	1533	126	70	131	71
361	1.15	3.58	0.9	1.91	0.7	0.81	1174	1682	115	68	107	64
362	2.11	5.32	1.02	3.15	1.24	1.25	1535	1542	125	71	126	72
363	1.05	3.94	1.23	2.22	1.09	1.08	1265	1092	113	66	117	69
364	1.31	5.77	1.08	4.01	1.21	1.23	1482	1559	145	86	150	97
365	0.76	3.53	1.47	1.54	1.17	1.25	1972	1833	141	85	142	83
366	0.81	2.82	1.06	1.63	1.02	1.14	1177	1269	111	69	106	64
367	1.51	4.65	0.94	2.91	1.1	1.14	1311	1287	104	67	105	62
368	1.32	3.27	0.8	1.94	0.94	0.96	1818	1742	132	76	139	79
369	1.39	3.04	0.84	1.55	1.16	1.09	1474	1399	121	61	122	61
370	2.08	4.07	0.85	2.43	1.12	1.13	1679	1636	134	78	135	76
371	1.8	3.84	1.21	1.98	1.15	1.24	1805	1729	145	87	148	84
372	1.63	2.45	0.55	1.23	1.08	1.05	1700	1727	165	76	169	87
373	1.6	5.28	1.6	4.05	1.15	1.08	1440	1548	149	78	169	88
374	0.93	4.96	1.28	3.11	1.16	1.15	2299	2373	156	88	145	82
375	2.54	4.9	0.72	3.02	1.03	1.04	1358	1374	117	79	118	78
376	1.17	2.49	0.13	1.32	1.15	1.07	1406	1435	134	87	142	87
377	1.55	6.6	0.96	4.68	1.17	1.09	1826	1813	140	79	140	74
378	0.88	3.05	1.06	1.58	1.07	1.21	1340	1378	134	77	132	76
379	0.67	3.6	0.96	2.17	1.17	1.1	2034	2021	118	71	120	68

380	2.51	4	1	2.02	0.96	1.04	1913	1964	158	89	162	91
381	2.55	4.93	1.19	2.82	1.11	1.16	2015	1906	140	69	139	70
382	2.25	3.07	0.74	1.53	0.88	1.04	1359	1473	125	78	131	82
383	2.77	2.77	0.86	1.48	1.24	1.23	1557	1626	120	72	117	75
384	0.84	3.22	1	1.74	1.17	1.18	1571	1405	119	67	119	67
385	1.76	4.59	0.97	2.98	1.16	1.12	1614	1602	128	73	130	74
386	0.86	3.33	1.51	1.4	1.2	1.21	1287	1179	123	74	117	68
387	1.21	3.54	0.92	2.19	1.18	1.16	1028	1029	105	55	105	55
388	0.87	2.81	1.07	1.43	1.02	1.11	2054	2120	153	86	156	86
389	0.86	4.08	1.34	2.22	1.06	1.02	2086	2038	140	94	143	95
390	0.95	4.43	1.03	2.97	1.2	1.25	1431	1414	106	68	106	70
391	0.89	3.26	1.22	1.43	1.14	1.14	1490	1451	118	72	114	71
392	2.58	4.47	0.8	2.55	1.11	1.13	1596	1463	120	73	98	64
393	1.09	2.3	0.84	1.03	1.14	1.16	1225	1294	119	77	125	79
394	0.69	3.47	0.84	2.13	1.18	1.2	1947	1838	138	89	146	90
395	0.75	3.13	0.93	1.87	1.22	1.19	1580	1510	138	72	129	65
396	0.47	3.04	1.62	1.79	1.22	1.25	1635	1668	123	72	124	71
397	1.19	5.23	1.18	3.27	1.13	1.09	1287	1267	116	79	118	80
398	3.15	5.33	0.89	3.5	1.2	1.17	1760	1824	138	86	141	87
399	4.67	5.27	0.82	2.55	1.12	1.08	1658	1727	118	73	115	74
400	1.03	5.15	1.24	3.35	1.15	1.18	1360	1318	115	60	114	64
401	1.31	3.82	1.89	1.89	0.78	1.08	1271	1477	118	58	113	57
402	0.66	2.97	1.03	1.48	1.23	1.19	1483	1500	137	75	131	70
403	1.12	5.02	1.6	2.86	1.16	1.17	1194	1231	126	68	124	67
404	0.89	3.09	1.19	1.39	1.03	1.03	1693	1513	121	63	116	62
405	1.44	3.31	0.8	1.5	1.14	1.1	1560	1661	153	91	154	92
406	3.92	5.07	0.93	2.72	1.26	1.28	1631	1624	137	84	140	85
407	1.88	3.57	0.65	2.15	1.2	1.14	1651	1587	140	72	138	71
408	0.81	2.81	0.95	1.46	1.17	1.12	1022	1019	89	52	89	56
409	0.66	2.47	1.05	1.08	1.07	1.19	2197	2192	132	78	134	78
410	1.83	2.62	0.59	1.52	1.15	1.18	1301	1231	114	72	112	71
411	0.89	1.58	0.82	2.41	1.27	1.17	1441	1515	113	73	114	69
412	0.7	2.83	0.75	1.76	0.98	0.87	1241	1283	131	71	127	72
413	1.25	3.99	0.88	2.68	1.06	1.18	1317	1283	120	77	118	75
414	1.14	2.47	0.98	1.05	1.11	1.07	1605	1560	125	60	123	61
415	1.51	4.11	0.88	2.6	1.21	1.23	1417	1389	124	66	123	63
416	1.22	4.08	0.88	0.88	0.91	1.07	1375	1334	135	81	136	77
417	1.51	4.83	1.11	3.16	1.22	1.23	2015	2010	136	77	132	74

418	1	3.55	0.62	1.8	1.23	1.22	1927	1857	147	87	148	89
419	1.09	2.9	0.91	1.58	1.2	1.15	1214	1126	105	63	105	63
420	0.71	2.89	0.94	1.59	1.23	1.3	2128	2136	148	85	140	82
421	2.33	4.95	1.3	2.05	1.28	1.29	1605	1745	139	73	134	71
422	4.65	10.95	0.73	2.65	0.9	0.98	1842	1819	199	109	192	106
423	0.73	2.61	0.79	1.39	1.3	1.27	1510	1556	143	85	142	80
424	1.4	3.56	0.98	2.09	0.89	1.02	1188	1402	143	73	152	72
425	1.04	2.61	0.91	1.39	1.13	1.07	2334	2386	139	97	135	95
426	2.53	6.43	1.29	3.64	1.06	1.05	1573	1574	139	85	135	81
427	8.09	3.92	0.64	1.67	1.27	1.33	1362	1411	107	71	109	72
428	1.48	3.96	0.95	2.42	1.22	1.21	1488	1493	122	64	125	64
429	1.59	4.54	0.91	2.85	1.2	1.14	1234	1219	92	54	90	52
430	2.04	2.47	0.5	1.16	0.85	0.93	1758	1715	147	77	138	75
431	0.88	3.07	1.01	1.67	1.11	1.06	1448	1457	130	73	131	74
432	0.48	4.37	1.64	2.4	1.21	1.17	1850	1914	136	74	131	71
433	0.97	4.33	0.98	2.96	1.26	1.06	1859	1892	131	79	129	78
434	2.31	4.17	1.2	2.18	1.18	1.19	1443	1462	105	67	106	70
435	0.89	3.07	0.96	1.72	1.16	1.11	1210	1179	114	80	114	77
436	4.31	3.53	0.62	1.5	0.91	1.06	1520	1866	150	79	154	82
437	2.72	4.9	0.91	2.28	1.13	1.1	1196	1161	122	69	118	69
438	1.22	3.57	0.83	2.39	1.04	0.97	1210	1234	114	67	113	67
439	0.94	3.4	1.33	1.63	1.22	1.27	1672	1609	150	97	150	98
440	0.81	3.17	1.18	1.41	0.95	1	2260	2157	183	89	173	83
441	1.72	3.99	0.6	2.53	1.01	0.9	1205	1126	126	72	128	74
442	1.1	4.59	1.02	3.22	1.18	1.18	1684	1650	129	77	131	74
443	0.66	4.76	1.35	3.02	1.09	1.3	2187	2038	116	66	114	60
444	1.58	6.1	1.29	3.59	1.14	1.11	2645	2864	154	73	159	74
445	1.17	2.57	0.88	1.15	0.89	0.9	2104	1808	134	72	134	72
446	4.22	3.76	0.61	1.33	0.73	0.98	1371	1403	133	73	132	74
447	1.54	2.42	0.93	1.02	1.21	1.15	1323	1293	107	60	113	69
448	1.5	4.93	1.31	3.05	1.11	1.11	1605	1580	128	66	129	61
449	3.23	4.62	0.74	2.25	1.01	1.15	1829	1713	111	65	115	65
450	3.04	3.03	0.66	1.4	1.12	1.12	1426	1435	129	74	130	75
451	0.9	5.36	1.93	2.99	1.1	1.06	1321	1387	108	61	115	66
452	1.93	6.63	1.43	4.42	1.15	1	1583	1523	137	85	143	86
453	0.97	5.41	1.94	3.87	1.23	1.19	1286	1307	124	69	125	70
454	0.99	5.3	1.13	1.75	1.09	1.13	1473	1562	123	67	127	66
455	1.1	3.32	1.33	1.55	1.01	1.12	1590	1593	127	82	129	78

456	1.45	4.22	0.87	2.74	1.02	1.04	1583	1638	131	77	135	79
457	2.54	3.83	0.82	1.82	1.27	1.19	1356	1344	132	80	131	80
458	1.33	2.64	0.82	1.35	1.1	1.14	1658	1649	149	86	145	81
459	1.23	4.27	0.74	3.01	1.18	1.14	1589	1555	138	72	136	74
460	2.36	4.24	0.69	2.49	1.14	1.18	1697	1642	112	68	110	72
461	0.85	4.96	1.33	3.23	1.05	1.1	1403	1298	122	69	124	76
462	1.82	5.87	1.26	3.75	1.07	1.11	1512	1614	134	78	131	77
463	1.2	3.2	0.96	2.01	1.21	1.21	1367	1371	127	79	131	82
464	2.9	2.9	0.94	1.36	1.2	1.19	1514	1587	123	80	121	77
465	0.89	2.58	0.83	1.35	1.21	1.2	1815	1791	127	72	128	73
466	0.77	4.51	1.35	2.75	1.16	1.17	1686	1653	120	71	120	73
467	1.01	3.42	0.9	2.14	1.19	1.21	1278	1259	126	72	124	72
468	0.72	4.03	1.22	2.32	1.21	1.21	1252	1262	117	64	121	66
469	0.6	3.96	1.31	2.31	1.18	1.15	1778	1803	130	81	130	81
470	1.81	3.81	0.91	2.35	0.83	0.93	1620	1756	123	68	116	68
471	0.54	4.1	1.83	1.84	1.11	1.11	1530	1570	120	67	113	60
472	3.19	5.66	0.91	3.21	1.12	1.12	1678	1600	154	89	154	89
473	1.29	3.36	0.69	2.27	0.81	1.15	1538	1547	122	67	127	64
474	1.39	5.43	1.02	2.63	1.11	1.13	1286	1242	120	69	119	67
475	1.3	4.81	1.32	3.04	1.05	1.06	1196	1193	104	62	99	55
476	0.74	5.2	1.16	3.36	1.09	1.09	1826	1763	135	78	137	77
477	2.09	3.67	0.77	2.01	1.13	1.1	1545	1535	118	70	119	71
478	1.5	3.45	1.28	1.61	1.01	1.09	1669	1887	130	65	118	56
479	3.91	3.78	0.58	1.47	1.05	1.04	1368	1400	128	79	132	83
480	1.37	3.2	0.82	1.77	1.27	1.18	1398	1471	137	98	124	97
481	1.27	4.87	0.87	3.11	1.05	1.11	1665	1643	128	65	140	76
482	1.81	5.39	0.92	3.35	1.22	1.2	1378	1448	120	80	115	79
483	3.61	1.12	0.58	2.31	1.07	1	1507	1554	119	68	117	68
484	0.69	2.54	0.93	1.31	1.01	0.91	1456	1488	111	63	92	64
485	1.05	2.86	1.29	1.3	1.09	1.17	1832	1792	133	72	128	69
486	0.61	2.73	0.86	1.53	1.18	1.17	1085	1034	107	68	107	69
487	0.97	4.89	1.23	2.93	1.12	1.14	1365	1400	112	58	111	54
488	1.29	3.03	1.54	1.14	1.27	1.12	1357	1390	116	71	113	70
489	2.14	4.15	0.72	2.8	1.05	1.01	1277	1264	126	75	128	85
490	3.07	4.81	0.88	2.62	1.16	1.19	1321	1301	118	65	116	63
491	0.66	5.82	1.71	3.56	1.19	1.33	1420	1455	124	74	126	74
492	1.28	4.61	0.92	3.31	1.06	1.17	1077	1092	110	70	116	73
493	3.55	4	1.13	1.66	1.16	1.14	1682	1731	130	78	134	82

494	1.85	5.18	1.13	3.14	1.2	1.2	1814	1904	132	72	130	68
495	1.12	4.32	1.59	2.27	1.18	1.29	1536	1503	121	68	114	69
496	2.17	4.33	0.83	2.79	1	1.07	1213	1217	110	68	113	65
497	0.95	4.97	1.24	3.21	1.03	1.04	1470	1442	140	69	144	70
498	2.34	4.12	1.06	2.34	1.17	1.2	1837	1750	138	72	138	74
499	0.96	4.16	1.18	2.5	1.04	1.09	1910	1998	169	100	162	93
500	1.46	5.19	1.19	3.47	1.2	1.29	1217	1174	118	89	120	77
501	3.29	4.63	0.85	2.46	1.15	1.15	1415	1455	136	78	130	78
502	1.67	4.09	0.73	2.72	1.21	1.23	1251	1262	112	67	110	68
503	1.3	5.75	1.29	3.75	1.17	1.16	1350	1297	103	61	101	60
504	1.11	3.89	1.36	1.94	1.11	1.23	1277	1260	107	70	104	68
505	1.24	3.72	0.79	2.51	1.15	1.23	1340	1319	100	66	102	64
506	1.42	5.61	1.17	3.73	1.17	1.21	1349	1394	113	66	117	69
507	1.16	4.86	1.34	2.94	1.11	1.15	1624	1592	114	60	111	61
508	1.62	4.39	1.2	2.73	1.1	1.1	1350	1321	124	81	121	77
509	2	4.25	0.9	2.46	1.25	1.24	1827	1900	137	80	144	86
510	2.03	2.55	0.68	1.06	1.06	1.07	1284	1288	124	78	121	78
511	1.45	2.49	1.09	0.95	1.16	1.2	1771	1791	133	79	134	77
512	1.6	7.7	1.01	3.25	1.05	1.07	1305	1297	122	80	120	77
513	1.81	4.9	1.16	3.27	1.06	1.27	1845	1918	140	87	139	86
514	1.81	5.52	0.95	3.5	0.82	0.82	1845	1797	164	79	157	72
515	4.24	5.3	0.89	2.39	1.12	1.06	1427	1465	118	67	120	67
516	0.74	3.31	0.87	1.91	1.18	1.17	1334	1330	125	82	123	82
517	3	3.8	0.65	2.34	1.17	1.46	1282	1361	125	72	126	75
518	0.68	3.37	0.83	3.44	1.19	1.15	1541	1538	127	71	129	70
519	1.77	4.1	0.92	2.41	1.14	1.09	1840	1945	134	67	134	71
520	1.97	3.89	0.97	2.2	0.94	1.09	1648	1637	139	82	136	86
521	1.29	4.7	0.99	3.09	1.12	1.06	1721	1737	146	93	146	98
522	0.9	2.72	0.84	1.52	1.22	1.23	1393	1419	134	74	131	76
523	5.05	3.89	0.52	1.93	1.06	1.1	1337	1346	110	77	111	76
524	2.47	4.08	0.74	2.31	1.08	1.19	1720	1793	145	80	148	83
525	1.3	3.5	0.99	1.98	1.1	1.11	1565	1562	124	77	122	78
526	1.2	3.82	0.89	2.32	1.33	1.21	1023	1011	98	52	98	55
527	1.83	6.58	1.24	3.56	1.08	1.05	1295	1361	123	67	130	70
528	1.07	4.12	1.08	2.43	1.27	1.23	1789	1775	144	72	138	81
529	1.88	3.76	0.84	2.06	1.24	1.24	1051	1055	110	56	106	57
530	1.09	3.69	1.03	2.07	1.25	1.19	1617	1590	116	64	124	67
531	19.22	9.95	0.61	1.52	1.21	1.08	1295	1322	131	79	123	79

532	0.5	3.1	1.27	1.5	1.23	1.2	1530	1496	122	76	122	76
533	1.34	3.31	1.01	1.83	1.15	1.11	1941	1979	125	69	131	73
534	0.75	2.06	0.55	1.18	0.97	1.05	1762	1717	107	62	102	62
535	1.51	4.11	1.2	2.13	1.31	1.19	1277	1255	107	62	107	60
536	0.89	2.83	0.76	1.61	1	1.05	2415	2335	157	92	149	101
537	1.14	3.21	1.06	1.6	1.12	1.11	1609	1602	134	75	131	76
538	0.66	3.73	1.5	1.87	1.26	1.2	1395	1486	114	61	120	58
539	2.06	3.72	1.03	1.95	1.07	1.17	1400	1436	125	79	123	71
540	0.65	5.71	0.86	3.4	1.12	1.15	1604	1701	137	82	133	81
541	2.11	5.56	1.1	3.71	1.15	1.13	1965	2194	139	68	132	67
542	2.35	4.76	0.86	3.01	0.96	0.94	1405	1425	106	73	99	62
543	0.94	2.86	1.04	1.35	0.59	0.64	1614	1420	120	55	116	55
544	1	3	0.97	1.64	1.09	1.18	1367	1353	143	86	149	88
545	2.29	3.79	0.86	1.32	1.12	1.13	1430	1533	148	92	143	90
546	1.63	2.75	0.84	1.3	1.08	1.05	2166	2309	123	78	128	79
547	2.24	4.76	0.7	3.1	1.19	1.15	1638	1628	101	65	132	76
548	0.45	2.87	0.94	1.64	1.09	1.18	1557	1602	141	70	139	70
549	1.53	4.81	0.96	2.83	1.12	1.12	1563	1613	128	67	129	70
550	2.08	4.68	0.82	2.89	0.96	1.01	2205	2009	164	75	144	78
551	0.89	3.44	1.34	1.83	1.02	1.06	1417	1420	118	67	119	51
552	0.78	3.03	1.11	1.58	1.12	1.1	1958	2032	151	83	153	88
553	1.65	4.25	0.84	2.75	1.08	1.11	1947	1854	127	78	131	80
554	1.68	4.52	1.21	2.47	1.1	1.04	1468	1436	130	89	136	95
555	1.79	4.52	0.75	2.91	1.12	1.16	1308	1312	136	57	136	55
556	1.62	3.32	0.7	1.8	1.13	1.11	1879	1748	134	73	132	70
557	0.65	2.79	1.34	1.06	1.22	1.26	1568	1634	115	70	116	67
558	1.64	5.95	1.38	3.69	1.11	1.05	1823	1828	135	82	141	87
559	7.48	7.47	0.92	2.87	1.08	1.03	1523	1639	149	87	151	85
560	1.8	4.64	0.79	3	1.15	1.13	1457	1510	140	94	138	93
561	6.96	4.54	0.76	1.83	1.27	1.24	1430	1442	132	85	141	89
562	1.21	4.66	0.31	3.46	0.98	1	1399	1448	128	77	117	77
563	4.07	5.03	2.8	2.8	0.97	1.05	1628	1620	147	79	146	79
564	1.08	3.37	1.07	2.16	0.91	1.01	949	968	70	51	82	44
565	1.81	3.07	0.63	1.71	0.66	0.56	2165	1242	119	56	98	49
566	0.82	3.48	1.13	1.87	1.05	1.03	1583	1585	121	71	117	67
567	1.18	2.48	0.91	0.89	1.28	1.21	1133	1164	121	64	120	65
568	2.9	4.62	0.89	2.81	1.15	1.15	1680	1621	135	83	138	84
569	0.84	1.11	1	1.98	1.03	0.97	1798	1532	149	82	141	80

570	1.06	2.87	1.01	1.5	1.25	1.17	1582	1588	131	74	130	72
571	1.16	4.66	1.01	0.92	1.12	1.14	1814	1783	122	65	144	81
572	1.41	4.11	1.06	2.6	1.15	1.12	1638	1652	129	78	136	85
573	0.99	2.68	0.69	1.5	1.32	1.02	2081	2037	124	76	127	81
574	2.46	4.67	1.3	3.15	1.24	1.22	1739	1779	133	69	136	74
575	2.55	3.61	1.11	1.63	1.13	1.13	1657	1636	114	60	116	63
576	1.69	3.45	1.04	1.76	1.09	1.14	1373	1427	123	66	132	74
577	1.11	3.4	1.27	1.68	1.07	1.13	2093	2487	138	69	131	64
578	1.57	3.87	0.86	2.24	1.16	1.28	1568	1591	124	70	125	71
579	1.94	5.64	0.95	3.69	0.97	0.95	1305	1267	118	69	119	72
580	2.33	3.74	0.8	1.98	1.17	1.21	1607	1594	122	67	125	70
581	1.13	3.69	1.49	1.46	1.17	1.2	1076	1100	87	56	89	60
582	3.6	3.53	0.91	1.25	1.01	1.04	1514	1501	141	73	138	69
583	2.6	4.87	0.72	2.59	1.21	1.26	1642	1626	121	73	120	74
584	3.09	3.09	0.57	1.47	1.15	1.3	1140	1251	101	65	104	68
585	1.14	6.59	1.19	4.89	1.02	1.11	1584	1461	168	96	172	96
586	1.65	3.44	3.97	1.92	1.03	1.03	1737	1634	149	86	147	87
587	1.1	5.58	1.33	3.65	1.17	1.21	1250	1245	107	62	105	64
588	2.2	3.53	0.99	2.16	1.14	1.22	1418	1348	97	59	98	59
589	1.05	3.36	0.94	1.93	1.12	1.15	1412	1490	128	86	124	86
590	0.78	2.41	0.85	1.24	1.17	1.14	1124	1092	104	66	102	66
591	1.55	5.09	0.85	3.79	1.28	1.2	1420	1522	108	60	108	61
592	0.89	3.12	1.53	1.05	1.13	1.1	1389	1395	144	81	143	79
593	2.4	3.27	0.85	1.66	1.2	1.17	1381	1365	126	67	127	70
594	1.41	2.83	0.83	1.52	1.15	1.17	1285	1343	108	63	108	69
595	2.31	2.47	0.62	0.87	1.11	1.14	1377	1495	110	65	102	62
596	1.39	5.57	0.83	3.93	1.26	1.17	1825	1756	135	88	138	86
597	0.94	2.65	0.96	1.19	1.09	1.06	1640	1727	126	69	127	69
598	1.79	4.51	1.4	2.53	1.18	1.16	1794	1840	131	68	131	62
599	2.33	4.07	0.76	2.45	1.13	1.11	1674	1829	142	77	147	77
600	0.86	4.23	1.42	2.4	1.19	1.23	1692	1770	114	58	113	55
601	1.8	3.01	1.13	1.16	1.29	1.36	1678	1669	132	84	137	87
602	1.12	3.96	0.79	2.54	1.11	1.13	1795	1846	141	74	138	70
603	5.09	5.09	1.39	2.99	1.14	1.16	1509	1500	111	62	112	60
604	0.16	4.03	1.7	1.95	1	1.1	1749	1765	123	63	138	71
605	1.19	3.97	1.9	2.57	1.22	1.19	1589	1582	120	66	120	66
606	2.21	4.67	0.88	2.99	1.19	1.22	1822	1852	155	93	156	89
607	1.05	2.56	0.87	1.39	0.93	1.07	1206	1164	119	68	124	70

608	0.82	4.19	1.5	2.17	1.24	1.24	1588	1646	121	66	125	62
609	1.32	5.12	1.06	3.49	1.14	1.19	1677	1683	120	72	118	72
610	0.62	2.84	0.97	1.6	1.07	1.11	1633	1661	121	68	120	71
611	0.84	6.1	1.25	4.24	1.24	1.22	1518	1535	129	78	127	70
612	1.26	3.66	0.87	2.33	1.16	1.19	1432	1430	121	75	123	74
613	1.46	5.29	1.16	3.6	1	0.9	1534	1481	118	69	120	66
614	2.04	2.93	0.75	1.41	1.19	1.18	1384	1436	124	78	132	83
615	0.71	4.24	1.68	2.04	1.13	1.2	1806	1875	123	72	123	67
616	0.99	4.15	1.08	2.83	1.21	1.25	1506	1391	95	51	94	56
617	1.82	3.42	0.95	1.87	0.78	0.78	1754	1743	144	82	141	77
618	0.74	4.63	1.08	3.11	1	1.11	1278	1363	121	64	120	65
619	1.45	3.96	1.07	2.26	0.61	0.86	1007	1448	138	76	140	66
620	1.59	0.76	1.91	1.91	0.98	0.98	1321	1337	93	60	94	62
621	1.13	5.66	1.22	4.21	1.1	1.13	1633	1597	134	81	133	82
622	2.12	4.51	0.98	2.65	1.05	1.07	1762	1710	140	68	139	69
623	4.21	5.08	0.74	2.58	1.21	1.18	1876	1992	163	94	165	94
624	2.38	4.83	0.89	2.73	1.31	1.16	189	1907	122	73	120	71
625	0.53	3.47	1.24	1.93	1.15	1.12	1608	1669	136	88	132	84
626	1.18	3.64	0.82	2.51	1.13	1.07	1817	1896	135	74	137	73
627	1.01	3.85	1.01	2.38	1.12	1.08	1697	1699	131	74	128	73
628	1.22	3.3	1.09	1.67	1.19	1.15	1804	1716	147	66	144	63
629	2.19	5.54	1.15	3.27	0.69	0.74	1734	1463	179	92	180	91
630	0.96	3.44	0.91	2.18	1.26	1.19	1446	1407	118	63	111	67
631	1.22	3.13	1.24	1.54	1.3	1.32	1228	1224	116	68	116	70
632	1.23	3.81	1.01	2.29	1.23	1.23	1545	1789	141	82	142	83
633	0.77	4.32	1.06	3.01	1.17	1.22	1371	1425	109	72	106	69
634	1.57	4.29	1	2.87	1.14	1.23	1813	1715	147	82	140	79
635	1.94	4.69	0.99	2.72	1.18	1.16	1607	1593	125	73	128	75
636	2.05	4	0.77	2.13	1.1	0.7	1346	993	125	69	119	71
637	2.16	5.15	1.1	3.3	0.96	1.02	1306	1418	105	60	103	65
638	1.36	3.69	0.77	2.44	1.26	1.17	1454	1423	117	80	116	77
639	0.85	5.52	1.69	3.35	1.07	1.01	145	1407	122	62	121	62
640	0.65	3.85	1.56	1.61	1.02	1.11	1229	1234	93	54	101	53
641	1.58	6.1	1.09	4.14	1.19	1.21	1654	1638	140	86	140	85
642	1.71	2.46	0.51	1.08	1.05	1.03	1819	1782	123	74	132	74
643	6.59	5.62	0.98	1.91	1.18	1.15	1423	1414	131	78	131	79
644	1.01	2.43	0.85	1.13	1.1	1.08	1225	1244	105	68	105	64
645	2.71	4.46	1.14	2.14	1.2	1.18	1324	1333	122	76	123	74

646	3.97	5.38	0.36	2.58	1.24	1.18	2072	2193	136	68	132	64
647	0.87	3.71	1.08	2.32	1	0.77	1331	1187	138	82	136	79
648	1.45	3.55	0.86	2.05	1.18	1.16	1518	1510	129	86	125	86
649	1.67	2.8	0.66	1.59	1.13	1.12	1286	1305	118	72	115	65
650	1.04	4.08	1.48	1.98	1.02	1.06	1291	1447	128	63	127	66
651	0.8	3.21	1.13	1.75	1.11	1.05	1653	1677	133	76	125	71
652	0.61	3.24	0.98	1.69	1.22	1.18	1524	1635	134	79	129	80
653	0.95	5.95	1.03	4.17	1.11	1.1	1154	1086	105	61	99	56
654	2.44	2.94	0.8	1.5	1.1	1.15	1544	1599	117	65	119	70
655	1.07	2.9	1.01	1.44	1.09	1.14	1612	1595	120	58	121	60
656	1.25	2.79	0.65	1.7	0.97	1.06	1391	1418	147	92	154	96
657	1.86	3.78	1.22	1.93	1.18	1.18	1805	1842	142	83	143	84
658	1.5	3.28	0.86	1.84	1.18	1.13	1431	1537	130	64	129	64
659	2.41	3.96	1.06	2.09	1.06	1.1	1409	1410	138	73	150	82
660	1.31	3.81	0.88	2.36	1.36	1.14	1633	1822	122	74	125	73
661	1.11	3.77	1.02	2.33	1.03	1.07	1776	1743	124	87	145	61
662	1.27	3.48	1.11	1.69	1.27	1.29	1588	1623	130	69	128	67
663	3.03	3.17	0.71	1.58	1.14	1.31	1468	1440	131	80	135	84
664	1.6	4.09	1.08	2.32	1.18	1.13	1586	1513	121	77	126	80
665	1.35	5.76	1.29	3.85	1.16	1.19	1807	1821	137	83	145	84
666	1.14	5.3	0.3	3.96	1.16	1.04	2092	2122	129	75	126	76
667	0.9	3.26	1.14	1.92	1.15	1.19	1990	1838	139	78	133	78
668	0.93	2.95	1.58	1.02	0.93	1.05	1770	1793	147	86	153	86
669	5.89	3.68	0.58	0.95	0.88	0.94	1656	1817	163	84	165	81
670	2.85	3.53	0.74	1.86	1.09	1.12	1361	1581	107	62	113	70
671	1.07	3.33	0.88	1.36	1.22	1.12	1687	1690	117	66	118	67
672	5.75	7.01	1.74	3.3	1.17	1.2	1432	1390	122	71	120	71
673	4.42	5.15	1.01	3.16	1.07	1.09	1842	1863	150	84	157	87
674	2.71	2.76	0.76	1.32	1.13	1.14	1525	1527	114	68	115	73
675	1.73	3.47	0.77	2.14	1.12	1.09	1308	1266	122	72	129	72
676	1.01	3.6	0.6	2.11	1.18	1.18	1170	1113	124	62	124	61
677	2.31	4.59	0.97	2.91	1.14	1.05	1097	1109	105	66	100	60
678	0.91	3.37	1.3	1.68	1.24	1.28	1515	1576	127	85	128	87
679	1.76	3.02	0.76	1.63	1.17	1.21	1827	1949	123	65	119	65
680	4.85	3.79	0.74	1.06	1.12	1.04	1058	1092	99	61	102	59
681	0.43	3.38	1.12	2.02	1.23	1.32	1414	1399	124	69	123	70
682	0.81	3.32	1.06	2.05	1.15	1.25	1987	1973	139	73	141	71
683	1.73	3.4	0.66	2.22	1.09	1.11	1501	1533	114	77	113	76

684	0.83	3.31	1.6	1.27	1.26	1.25	1524	1548	119	65	121	65
685	1.62	3.09	0.97	1.58	1.28	1.26	1213	1215	104	65	105	62
686	3.49	6.66	0.97	3.59	1.25	1.14	1346	1376	119	74	116	67
687	0.88	6.39	1.21	4.24	1.24	1.25	1656	1639	128	67	128	62
688	1.3	2.7	0.75	1.59	1.28	1.12	1808	1828	114	73	114	81
689	1.48	4.29	1.01	2.78	1.2	1.16	1188	1159	99	57	102	59
690	1.38	1.51	1.17	2.77	1.19	1.15	1794	1695	133	69	137	77
691	1.73	5.52	0.87	3.99	1.24	1.14	1452	1353	125	78	132	78
692	0.56	3.03	1.3	1.51	1.19	1.23	1365	1347	115	63	112	65
693	0.99	3.51	1.21	1.64	1.19	1.23	1751	1811	136	75	137	76
694	2.61	6.17	1.17	4.2	0.97	0.96	1276	1247	115	60	113	76
695	1.02	3.5	1.14	2.09	1.07	1.1	1011	1056	107	62	110	59
696	1.59	4.64	1.15	3.06	1.15	1.1	1567	1550	122	73	121	77
697	1.47	3.29	0.91	1.84	1.2	1.16	1597	1643	117	68	115	69
698	1.68	3.55	0.87	2.13	1.12	1.27	1392	1333	118	80	121	83
699	1.53	4.84	0.9	3.32	1.16	1.1	1411	1460	124	71	128	72
700	1.51	4.97	1.11	3.18	0.94	1.05	1087	1166	114	80	124	79
701	13.57	7.39	0.78	1.58	1.17	1.22	1791	1734	144	80	145	83
702	2.01	3	0.95	1.29	1.22	1.21	1550	1551	115	76	117	78
703	0.59	2.92	1.16	1.27	1.18	1.07	1885	2077	162	79	147	77
704	1.2	10.26	1.11	8.21	1.06	1.09	1475	1489	130	80	133	80
705	0.96	4.12	1.18	2.67	1.14	1.15	1240	1424	129	70	132	74
706	2.51	2.66	0.55	2.96	0.9	1.49	2345	2306	143	81	152	84
707	4.58	4.58	1	2.62	1.07	1.13	1060	1030	131	91	137	89
708	2.28	1.63	0.8	3.84	1.15	1.15	1497	1529	114	72	113	73
709	1.05	4.13	1.05	1.4	1.11	1.11	3051	2191	151	89	148	85
710	1.75	4.34	1.12	2.61	0.72	0.81	1762	1761	136	70	138	74
711	1.28	3.98	1.15	2.26	1.06	1.03	1488	1452	131	84	142	86
712	3.21	5.66	0.82	3.6	1.11	1.12	1459	1489	130	81	132	79
713	0.76	3.8	1.05	2.56	1.08	1.02	1662	1672	107	70	127	85
714	2.2	4.55	0.88	2.79	1.08	1.1	1122	1133	117	76	120	69
715	1.19	3.88	0.93	2.51	1.18	1.14	1546	1580	138	94	143	96
716	0.8	3.51	0.86	2.49	1.26	1.28	1855	1888	136	72	138	74
717	1.09	4.45	1.31	2.64	0.96	1.13	2224	2418	143	76	139	82
718	1.63	4.29	0.81	2.72	1.11	1.13	1586	1597	125	82	125	80
719	1.24	4.23	3.27	2.94	1.15	1.18	1644	1639	141	81	139	76
720	1.06	3.61	0.85	2.37	1.05	1.07	1181	1131	112	67	114	67
721	1.12	3.01	0.85	1.59	1.23	1.34	1541	1574	123	74	124	75

722	1	3.98	0.98	2.58	1.15	1.09	1199	1242	111	71	116	70
723	1.08	2.48	0.68	1.86	1	1.2	1729	1690	101	63	100	65
724	0.94	4.56	1.34	2.75	1.19	1.12	1567	1529	147	89	146	87
725	1.08	3.32	0.87	1.83	0.99	0.99	1365	1384	122	70	134	75
726	2.84	4.4	0.87	2.33	1.2	1.2	1387	1357	113	72	113	68
727	0.93	3.22	1.29	1.54	1.14	1.16	2024	2181	150	70	153	76
728	1.67	0.5	1.65	3.79	1.22	1.19	1964	1923	151	94	156	98
729	1.51	4.13	0.9	2.19	1.01	1.05	1516	1395	141	82	137	82
730	1.82	4.25	1	2.57	1.07	0.99	1768	1782	123	80	126	79
731	4.65	3.53	0.85	1.23	1.23	1.21	1722	1730	166	95	162	96
732	1.85	4.5	1.16	2.74	1.19	1.15	2073	2130	151	83	149	81
733	1.22	3.26	0.72	1.98	1.13	1.14	2178	2157	143	75	153	77
734	1.26	3.17	1.21	1.42	1.2	1.21	2144	2191	137	62	120	50
735	2.52	6.59	1.08	4.52	0.92	0.94	2326	2265	126	80	145	82
736	0.96	2.79	0.79	1.53	1.04	1.16	1842	1844	140	70	146	73
737	0.75	4.71	1.07	3.39	1.1	1.13	1677	1630	135	79	131	79
738	1.17	3.32	1.09	1.87	0.99	1.07	1992	1886	147	86	152	86
739	1.5	2.85	1.17	1.08	1.1	1.13	1342	1319	131	70	133	72
740	4.51	4.51	1.07	2.84	1.14	1.11	2410	2357	151	81	147	81
741	2.31	5.33	1.02	3.47	1.19	1.16	1494	1505	129	62	125	59
742	1.83	5.51	1.31	3.45	1.14	1.18	1829	1823	129	79	133	81
743	0.64	3.61	1.31	2.06	1.1	1.18	1609	1615	144	73	141	70
744	0.88	2.98	1.05	1.56	1.02	1.04	1222	1188	122	68	128	71
745	4.48	3.77	0.95	1.39	1.07	1.07	1519	1450	111	65	107	65
746	4.63	4.37	1.04	1.78	1.14	1.17	2041	2150	140	81	144	80
747	2.07	4.54	0.68	3.1	0.56	0.49	886	847	107	65	105	64
748	1.27	4.88	0.87	3.29	1.17	1.11	1599	1660	128	69	133	74
749	0.55	3.02	1.18	1.59	1.13	1.18	1158	1066	111	63	114	65
750	5.3	6.79	0.9	2.6	1.01	1.14	1689	1715	152	99	152	99
751	1.36	5.11	1.84	2.26	1.16	1.11	2071	1932	148	73	146	75
752	2.01	6.64	1.4	4.45	1.21	1.26	1247	1269	104	53	103	49
753	1.98	3.74	0.75	2.48	1.04	1.07	1312	1289	109	74	114	80
754	0.73	3.6	1.4	1.69	1.23	1.21	1597	1500	140	64	143	64
755	2.45	5.86	1.15	3.84	1.12	1	1416	1463	120	74	120	66
756	1.06	4.04	1.17	2.47	1.11	1.26	1904	1820	122	67	124	75
757	2.8	4.01	0.97	2.15	1.27	1.23	1519	1547	130	73	132	78
758	3.02	5.13	0.84	2.84	1.23	1.18	1130	1302	112	74	113	73
759	1.77	3.83	1.65	0.47	1.14	1.16	1257	1284	111	70	113	67

760	2.15	5.32	0.82	3.58	0.49	0.64	733	1608	114	71	125	71
761	2.01	4.04	0.87	2.36	1.12	1.15	1319	1302	123	75	118	71
762	4.18	3.65	0.9	1.62	1.13	1.19	1662	1606	136	82	132	81
763	0.85	4.07	1.11	2.15	1.08	1.08	1175	1264	108	70	108	69
764	1.08	3.1	0.81	1.98	1.03	1.09	1440	1404	126	73	119	70
765	1.58	4.57	1.16	1.43	1.23	1.16	1531	1540	127	74	116	70
766	1.45	6.57	1.39	4.04	1.05	1.06	1163	1162	107	51	107	51
767	1.3	4.61	1.11	2.9	1.13	1.2	1706	1693	128	71	127	67
768	0.8	3.43	1.58	1.45	1.2	1.2	2072	2099	138	79	142	75
769	1.26	5.81	1.34	3.79	1.04	1.1	1492	1594	141	75	134	72
770	1.1	4.67	1.36	1.67	1.29	1.22	1473	1517	147	79	147	80
771	1.02	3.8	1.08	2.32	1.21	1.31	1368	1333	114	68	116	67
772	1.42	3.28	1.93	1.93	0.68	0.85	2538	1915	168	89	159	82
773	1.25	4.56	0.93	3.03	1.25	1.19	1148	1168	95	58	94	55
774	1.97	2.92	0.59	1.86	0.99	0.79	1800	1426	142	81	145	81
775	1.07	3.72	0.94	2.44	1.14	1.18	1312	1359	114	67	118	67
776	1.12	4.9	1.42	2.91	1.06	0.88	1745	1744	115	52	123	56
777	1.37	2.86	0.83	1.47	1.27	1.26	1250	1304	118	71	120	76
778	2.54	3.75	0.69	1.93	0.94	1.1	1463	1331	131	73	136	73
779	1.29	4.58	0.91	3.08	1.12	1.21	1303	1367	110	64	109	58
780	0.74	3.02	0.77	1.93	1.16	1.14	2743	2279	154	96	160	94
781	6.41	6.9	1.1	2.28	1.22	1.16	1649	1556	130	69	130	69
782	1.33	3.78	0.73	2.56	1.13	1.13	1402	1473	118	76	120	95
783	0.72	2.5	0.87	2.31	0.93	1.18	1455	1537	118	69	125	70
784	0.73	6.83	1.86	4.24	1.12	1.11	1555	1530	110	65	105	61
785	1.6	6.08	1.48	3.93	1.07	1.13	1471	1510	106	61	104	58
786	2.7	5.56	1.26	3.14	1.11	1.13	2548	2397	132	66	136	70
787	2.19	4.81	1.2	2.74	1.06	1.12	1076	1079	109	65	113	64
788	0.58	4.41	1.39	2.52	1.26	1.23	1512	1491	110	57	112	55
789	1.2	5.06	1.39	3.14	1.33	1.21	1573	1647	146	72	150	76
790	0.47	2.52	1.29	1.29	0.66	0.45	2666	2145	106	49	103	47
791	0.85	2.48	0.9	1.19	1.18	1.13	1662	1500	126	78	126	75
792	0.84	2.58	0.95	1.12	1.05	1.03	1374	1367	115	76	113	74
793	1.68	4.3	1.02	2.7	1.2	1.22	1789	1746	165	81	164	77
794	3.38	4.13	1.09	1.98	1.05	1.11	1529	1476	127	49	132	76
795	1.66	3.99	1.03	2.25	1.07	1.11	1213	1142	119	72	122	74
796	0.82	5.91	1.57	3.86	1.09	1.03	1793	1879	125	75	128	73
797	3.83	5.15	0.73	3.19	1.12	1.16	1401	1356	119	70	124	69

798	1.76	5.43	0.79	3.89	1.33	1.24	1429	1445	128	70	144	79
799	1.68	5.38	0.98	3.6	1.16	1.19	1213	1305	124	68	119	67
800	1.73	3.48	0.87	1.89	1.17	1.02	1144	1068	102	66	101	66
801	1.08	3.09	0.93	1.9	1.16	1.18	1650	1573	127	81	127	84
802	2.13	7.62	1.73	4.95	1	0.97	1081	1095	99	63	94	61
803	1.15	4.53	1.12	2.64	1.05	1.07	1775	1959	155	72	142	78
804	1.48	7.65	1.71	5.27	1.01	1.01	1864	1732	141	82	137	79
805	1.17	3.49	0.97	2.27	0.77	0.79	1582	1458	135	63	151	61
806	2.72	3.78	0.95	2.02	1.13	1.15	1296	1294	122	74	118	73
807	3.32	3.35	1.38	1.38	0.98	0.87	1430	1109	99	63	104	62
808	3.93	4.11	0.78	2.15	1.12	1.13	1254	1279	116	74	116	75
809	2.98	4.45	0.67	2.81	0.94	0.93	1178	1146	118	64	125	64
810	1.01	3.24	1	1.8	1.27	1.29	1496	1485	114	57	112	57
811	3.95	5.16	0.93	3.24	1.23	1.22	1365	1371	113	81	116	80
812	3.78	3.85	0.76	0.76	1.09	1.1	2006	1892	161	91	165	96
813	2.33	5.23	0.87	3.36	1.15	1.14	1641	1606	146	84	142	86
814	1.17	3.5	1.15	1.88	0.87	1.11	1525	1450	125	64	127	68
815	0.74	2.93	0.96	1.58	1.22	1.27	1451	1426	121	72	120	73
816	1.03	5.14	1.06	3.4	1.12	1.1	1630	1653	131	83	136	83
817	2.36	3.65	0.67	1.87	0.67	0.73	3047	1411	126	62	121	59
818	1.75	3.3	1.2	1.44	1.1	1.17	1375	1436	118	70	120	73
819	2.29	3.63	0.83	2.14	1.08	1.09	1305	1325	120	83	116	80
820	1.67	3.36	1.02	1	1.16	1.03	1493	1676	158	100	153	94
821	3.18	4.15	0.86	2.52	1.18	1.22	1232	1352	129	81	130	83
822	7.42	6.88	1.13	3.23	0.99	1.01	1876	1894	158	86	152	87
823	2.09	3.46	1.07	1.63	1.21	1.34	1325	1314	104	65	100	65
824	0.33	2.82	1.45	1.07	1.11	1.07	1267	1165	107	64	107	69
825	1.19	2.99	0.77	1.68	0.83	1.08	1372	1363	139	83	152	89
826	0.96	3.85	1.4	1.93	1.15	1.16	1587	1457	113	57	111	63
827	1.82	2.68	0.88	1.22	1.18	1.18	1808	1789	126	82	126	83
828	0.74	4.59	1.42	2.78	1.01	1.07	1695	1872	142	80	138	76
829	2.54	5.27	1.08	3.59	0.76	0.98	1485	1728	127	70	126	71
830	2.21	3.62	0.71	2.08	1.13	1.03	1609	1386	115	63	113	57
831	0.93	3.18	0.96	1.76	1.09	1.07	2307	2262	140	77	138	73
832	2.01	5.11	1.27	2.52	1.14	1.18	2053	2249	161	96	160	100
833	1.41	4.49	0.81	3.16	1.32	1.36	1347	1322	124	71	123	70
834	1.28	4.34	0.71	3.18	1.13	1.15	1461	1408	113	77	117	79
835	2.41	4.46	0.78	2.35	1.15	0.86	1509	1454	133	73	132	69

836	1.8	1.8	0.82	1.9	1.11	1.11	2021	1921	146	94	150	94
837	1.77	4.6	1.07	2.85	1.04	0.93	1820	1896	134	76	134	74
838	2.11	4.21	0.96	2.69	1.03	1.1	1263	1234	117	70	124	77
839	0.61	3.26	1.03	1.89	1.15	1.11	1074	1147	96	56	95	55
840	1.52	3.56	1.05	2.02	0.99	1.09	1713	1738	138	74	140	73
841	2.25	4.89	0.78	3.25	1.21	1.17	2559	2733	137	88	135	84
842	0.73	2.96	0.85	2.18	1.16	1.13	1653	1548	143	79	141	81
843	0.95	3.63	0.88	2.18	1.1	0.99	1375	1454	107	72	104	69
844	1.67	5.17	1.2	3.2	0.99	0.94	1998	2037	124	67	123	66
845	1.24	3.53	1.31	1.74	1.11	1.21	1573	1601	139	98	140	95
846	1.55	4.17	0.91	2.58	1.14	1.11	1644	1686	149	90	144	81
847	0.95	0.95	1.03	0.78	1.17	1.16	1863	2064	138	74	137	73
848	1.1	4.23	1.14	2.61	1.15	1.18	1964	2329	132	70	141	78
849	0.85	3.67	1.1	2.12	1.14	0.81	1665	1747	142	79	147	82
850	1.75	1.75	4.04	4.04	1.19	1.11	1788	1617	98	55	97	56
851	1.98	4.16	1.4	2.23	1.02	1.11	1251	1280	127	79	129	75
852	1.11	5.11	1.15	3.74	1.14	1.05	1647	1637	121	62	123	64
853	1.76	4.16	0.92	2.61	1.22	1.09	1481	1445	119	71	126	73
854	1.92	6.57	1.25	4.22	1.12	1.13	1716	1729	136	88	136	88
855	1.32	3.68	1.08	2.08	1.17	1.09	1778	1832	120	65	121	62
856	1.76	4.76	1.19	2.97	1.23	1.23	2100	2156	143	74	146	76
857	1.99	3.81	0.98	2.14	1.15	1.17	1923	1896	124	65	119	63
858	3.79	4.41	0.83	1.84	1.11	1.11	1336	1316	117	68	114	70
859	1.24	3.95	0.98	2.64	0.88	1.08	1673	1698	130	75	130	81
860	1.98	4.78	0.91	3.3	1.23	1.2	1275	1268	136	86	137	79
861	0.48	2.95	1.02	1.47	1.31	1.3	1973	1939	136	72	137	69
862	0.84	4.6	1.6	2.43	1.07	1.12	1349	1440	130	76	128	68
863	2.08	5.65	1.23	3.46	1.03	1.02	1315	1292	111	76	108	75
864	0.96	2.36	0.88	1.08	1.2	1.2	1667	1480	120	70	123	68
865	0.96	4.48	1.01	3.09	1.25	1.17	1390	1437	138	80	139	84
866	1.56	3.54	1.18	1.54	1.15	1.14	1731	1680	124	71	117	67
867	1.26	3.96	1.62	1.62	1.12	1.09	1237	1173	117	67	114	69
868	0.87	3.67	1.09	2.37	1.19	1.25	1257	1330	109	56	103	51
869	0.89	3.03	1.18	1.47	1.14	1.17	1195	1163	102	64	102	61
870	1.4	4.76	1.01	3.18	1.16	1.18	1270	1326	121	72	113	70
871	1.38	4.72	1.25	2.89	1.19	1.31	1218	1247	99	57	107	62
872	0.97	4.97	1.69	2.66	1.17	1.16	1417	1437	126	70	124	68
873	1.91	4.92	1.09	3.04	1.25	1.25	1065	1006	109	56	114	60

874	1.73	7.15	1.55	4.11	1.17	1.17	1661	1700	126	67	122	67
875	0.96	2.43	0.86	1.2	1.17	1.19	1448	1437	110	63	105	79
876	1.6	3.3	1.14	1.75	1.1	1.08	1353	1434	119	69	128	73
877	1.41	4.7	1.23	3.02	1.11	1	1264	1288	106	60	104	61
878	1.25	4.33	0.99	2.86	1.19	1.14	1336	1399	107	74	108	74
879	2.56	6.08	1.51	3.31	1.11	1.13	1794	1802	136	76	136	79
880	4.88	5.25	0.82	2.15	1.12	1.14	1999	2081	146	78	146	75
881	1.07	4.21	1.11	2.69	1.07	1.1	1543	1540	153	75	144	68
882	1.29	4.09	2.48	2.48	1.07	1.12	1577	1602	122	78	120	76
883	0.81	3.44	1.21	1.7	1.11	1.1	1146	1174	104	55	105	53
884	0.59	3.29	1.26	1.76	1.15	1.17	1304	1277	126	77	122	75
885	1.03	5.04	1.04	5.04	0.52	0.58	814	555	127	69	127	66
886	0.78	2.85	1.05	1.43	1.14	1.16	1249	1282	128	86	128	86
887	1.08	5.02	1.68	2.53	1.32	1.22	1564	1500	129	70	130	71
888	5.64	5.84	0.97	3.57	1.26	1.26	1491	1492	140	88	139	84
889	2.45	5.36	0.82	3.26	1.1	1.1	1373	1346	125	83	123	80
890	3.4	3.4	0.88	1.92	1.11	1.12	2029	2107	134	71	127	65
891	2.36	4.24	0.72	2.76	1.13	1.17	1663	1658	142	90	143	92
892	2.44	4.22	0.89	2.38	1.23	1.17	1461	1552	130	68	132	72
893	0.71	2.03	0.52	1.2	1.19	1.14	1768	1874	103	55	112	57
894	2.2	4.07	0.66	2.56	1.13	1.08	1789	1834	156	88	160	89
895	1.36	4.86	0.91	3.02	1.13	1.03	2323	2321	149	83	147	84
896	1.16	3.33	1.01	1.49	1.07	1.1	1776	1777	136	83	139	81
897	2.4	3.57	1.03	1.74	1.16	1.15	1425	1373	112	71	117	87
898	1.41	3.58	0.92	2.08	1.1	1.08	1369	1337	143	80	149	85
899	1.3	3.57	0.89	2.38	1.12	1.09	1482	1397	117	78	119	79
900	1.73	5.06	0.92	5.06	1.15	1.21	1971	1970	136	63	139	66
901	1.77	3.51	1.29	1.51	1.02	1.14	2146	2165	130	76	136	79
902	1.34	3.27	1.19	1.52	1.22	1.28	1636	1592	142	80	148	80
903	0.71	4.11	1.45	2.15	1.12	1.16	1347	1371	108	66	109	61
904	0.89	3.4	1.04	1.86	0.99	1.02	1180	1340	114	67	112	64
905	1.22	6.17	1.07	0.94	1.16	1.14	1695	1782	154	81	152	76
906	1.83	2.86	0.58	1.6	1.09	1.09	1574	1431	112	58	114	58
907	1.74	4.31	1.27	2.49	1.24	1.25	1665	1584	165	90	167	92
908	0.82	3.38	1.25	1.69	1.16	1.15	1427	1344	110	58	102	58
909	2.4	3.58	0.81	1.86	0.99	0.83	1683	1568	149	66	144	66
910	2.1	2.59	0.94	1.21	1.13	1.11	1286	1269	117	76	119	71
911	1.11	3.96	0.86	3.96	1.32	1.18	1306	1306	113	70	114	71

912	0.93	3.87	1.08	2.49	1.19	1.17	1628	1622	143	89	140	88
913	1.19	4.18	1.41	2.22	1.27	1.25	1654	1656	120	71	123	71
914	1.99	5.13	1.19	3.11	1.15	1.17	1365	1483	113	76	114	78
915	0.93	2.93	0.95	1.55	1.25	1.1	1598	1696	134	82	140	88
916	1.08	3.97	0.92	2.79	1.12	1.14	1962	1871	147	75	132	70
917	1.54	5.5	1.4	3.39	1.19	1.21	2188	2257	150	78	154	81
918	2.03	3.54	0.85	2.01	1.02	1.05	1290	1392	117	72	120	75
919	1.21	4.75	0.97	3.29	1.03	0.94	1413	1518	130	75	128	77
920	0.99	4.43	1.27	2.76	1.08	1.14	1919	1855	115	77	112	77
921	1	2.52	1.03	2.52	1.13	1.09	1512	1539	122	68	126	71
922	1.01	2.31	0.92	0.91	1.13	1.11	1516	1497	109	58	110	59
923	0.73	3.82	1.01	2.55	1.11	1.11	1380	1467	148	86	146	83
924	0.52	3.7	1.32	2.09	1.08	1.04	1597	1412	131	82	128	61
925	0.78	2.32	0.78	1.25	1.19	1.13	1303	1330	125	74	122	72
926	1.27	2.7	0.8	1.42	1.08	1.1	1286	1310	118	79	121	75
927	2.17	2.94	0.88	1.15	1.17	1.19	1378	1464	129	74	125	72
928	3.86	4.68	0.86	2.73	1.11	1.14	1356	1411	134	92	138	91
929	1.37	4.22	1.07	2.68	1.12	1.13	1394	1334	126	72	122	75
930	3.63	3.93	0.89	1.47	1.09	1.14	1742	1798	159	75	160	75
931	1.62	5.06	0.97	3.1	0.98	0.71	1864	1261	181	85	180	82
932	3.46	2.8	0.83	1.06	1.12	1.17	1110	1044	120	70	121	68
933	0.75	4.24	1.65	2.14	0.51	0.95	1328	1790	112	54	108	54
934	1.32	3.75	0.94	2.3	1.05	1.06	1421	1504	122	69	120	68
935	2.82	4.54	0.91	2.44	1.12	1.08	1408	1376	123	77	132	83
936	1.78	3.95	0.91	2.33	1.1	1.12	1698	1884	124	79	125	77
937	1.1	4.39	1.34	2.4	1.03	0.95	1176	1077	120	72	120	64
938	0.75	3.05	1.19	1.54	1.21	1.21	1572	1548	126	71	124	66
939	7.3	2.21	0.63	0.91	1.25	1.21	1740	1626	150	89	159	94
940	2.65	3.13	0.93	1.38	1.08	1.13	1613	1541	131	74	125	74
941	2.07	3.07	0.68	1.81	1	1.1	1673	1712	142	78	137	82
942	0.93	4.36	1.11	2.71	1.22	1.26	1196	1224	141	91	140	91
943	2.5	4.77	1.2	2.67	1.2	1.17	1680	1849	136	82	138	83
944	0.81	4.18	1.64	2.14	1.18	1.16	1623	1687	128	72	133	75
945	1.97	4.14	0.96	2.49	1.22	1.28	1487	1491	129	78	131	79
946	0.56	4.51	1.37	2.71	1.28	1.2	1456	1418	116	59	116	61
947	0.82	3.44	1.48	1.49	1.13	1.19	1549	1318	126	78	123	78
948	0.59	3.25	1.16	1.78	1.18	1.22	1490	1497	123	67	119	63
949	1.19	4.69	1.25	2.8	1.17	1.15	1499	1546	102	62	100	59

950	0.69	3.75	1.14	2.32	1.09	1.16	1409	1405	127	72	126	70
951	0.66	2.61	1.15	1.16	1.17	1.15	1658	1468	109	55	108	53
952	2.91	4.03	1.01	1.96	1.1	1.15	1878	1882	160	98	162	99
953	1.44	5.05	1.5	2.53	1.01	1.12	1529	1663	146	82	143	82
954	0.87	3.59	1.11	2.21	1.15	1.15	2360	2351	118	67	123	72
955	0.58	2.78	1.19	1.31	1.07	1.1	1255	1163	104	55	98	48
956	2.38	4.1	0.94	2.23	1.11	1.02	1648	1612	165	86	171	89
957	1.39	5.05	1.14	3.25	1.23	1.23	1454	1410	136	81	133	77
958	1.55	3.59	0.76	2.42	1.24	1.21	1322	1335	109	67	111	67
959	2.05	4.56	1.26	2.77	1.12	1.15	1760	1832	123	68	129	83
960	1.05	3.66	1.39	1.88	1.08	1.14	1725	1678	144	82	129	80
961	1.73	2.63	1.05	1.95	1.12	1.12	2797	2803	146	74	147	79
962	1.86	5.3	0.97	3.64	1.12	1.17	1416	1529	126	80	133	84
963	1.13	4.29	1.88	1.9	1.23	1.18	1645	1610	142	77	141	79
964	1.33	4.25	1.52	2.03	1.16	1.14	1275	1185	133	83	136	81
965	1.49	3.99	0.8	2.84	1.13	1.18	1676	1659	142	85	137	83
966	0.76	5.98	1.14	4.77	1.16	1.18	1348	1355	111	72	111	66
967	1.88	4.67	1.02	3.05	1.08	1.11	1186	1173	124	78	140	81
968	1.22	4.54	0.9	3.16	0.89	0.87	1241	1144	131	76	159	84
969	1.69	3.59	1.02	2.06	1.21	1.2	1548	1595	141	78	110	67
970	1.4	4.92	1.45	2.89	1.17	1.15	1672	1681	131	66	118	69
971	2.04	4.12	0.93	2.2	1.08	1.1	1478	1481	151	78	126	67
972	1.75	3.13	0.63	1.52	1.14	1.1	1467	1396	115	70	131	75
973	1.55	3.81	0.99	2.16	0.99	0.99	1272	1245	105	59	115	66
974	0.69	3.91	1.39	2.06	1.16	1.11	1866	1948	155	81	139	79
975	2.12	3.99	0.89	2.11	1.11	1.12	2269	2392	137	86	127	82
976	2.66	6.1	1.05	3.96	0.94	0.94	1288	1293	132	82	122	79
977	0.73	3.35	0.95	1.95	1.16	1.23	1843	1824	122	70	122	70
978	1.85	3.41	0.78	2.13	1.1	1.08	1087	1115	101	62	106	65
979	1.87	3.79	0.97	2.19	1.27	1.27	1519	1545	128	71	125	71
980	1.13	3.79	0.89	2.28	1.19	1.19	1293	1318	117	62	118	64
981	0.61	3.09	1	1.85	1.23	1.26	1734	1784	131	81	132	78
982	1.23	4.53	1.16	2.87	1.23	1.23	1443	1438	124	66	124	72
983	5.3	5.08	1.02	2.67	1.28	1.34	1655	1669	117	69	123	71
984	1.36	4.18	1.22	2.31	1.19	1.15	2322	2353	169	80	165	73
985	1.18	2.58	1.21	0.94	1.1	1.06	1798	1804	140	83	133	80
986	0.72	3.01	1.05	1.63	0.99	1.06	1220	1180	117	67	108	67
987	1.24	3.59	1.18	1.78	1.22	1.23	1465	1533	109	74	109	73

988	0.7	4.89	1.26	2.88	1.13	1.18	2479	2443	129	68	125	63
989	1.16	4.39	1.05	2.55	1.25	1.23	1751	1772	147	82	149	84
990	2.2	4.71	1.08	2.52	1.09	0.98	1645	1859	125	59	115	54
991	1.65	2.5	0.72	1.52	1.33	1.44	1911	1889	120	68	132	70
992	1.75	2.99	0.72	1.54	1.17	1.19	1622	1631	124	75	126	76
993	0.84	3.79	0.82	2.45	1.2	1.27	1470	1576	131	78	131	75
994	1.05	4.18	1.06	2.61	1.13	1.21	1691	1649	130	77	130	74
995	1.17	4.89	1.1	3.26	0.96	0.94	1808	1770	173	99	147	98
996	7.35	6.52	1.26	2.89	1.1	1.13	1352	1292	115	79	115	80
997	2.19	4.56	0.99	2.58	1.09	1.14	2152	2057	139	87	133	84
998	1.75	3.84	1.17	2.06	1.22	1.28	1385	1355	120	64	116	70
999	1.55	3.15	1.02	1.41	1.07	1.12	1313	1278	116	72	121	76
1000	1.69	4.58	1.06	2.68	1.17	1.12	1895	1991	132	72	130	72
1001	2.05	4.53	0.97	2.68	1.14	1.16	2198	2166	170	94	170	92
1002	1.21	3.62	1.24	2.17	1.06	1.15	1337	1346	126	83	125	83
1003	1.23	2.99	0.93	1.59	1.14	1.11	1393	1408	113	76	114	78
1004	1.59	5.62	1.33	3.21	1.31	1.28	1851	1710	166	97	163	91
1005	1.34	4.04	0.93	2.65	1.13	1.14	1834	1938	160	79	158	86
1006	0.69	3.85	1.01	2.53	1.16	1.15	1200	1214	119	68	117	68
1007	1.28	2.81	0.98	1.32	1.23	1.29	1758	1707	133	70	126	69
1008	2.16	4.07	0.83	2.55	1.13	1.23	1343	1297	114	63	122	66
1009	1.68	4.41	0.79	2.66	1.32	1.66	2161	2118	108	63	144	74
1010	1.29	2.49	0.92	1.23	1.14	1.27	1406	1416	132	80	123	81
1011	0.64	3.47	1.47	1.62	0.98	1.02	1406	1399	122	67	119	63
1012	3.32	8.07	1.25	4.7	1.12	1.17	1407	1548	121	73	117	65
1013	0.65	6.53	3.26	2.57	1.13	1.2	2481	2491	140	79	142	76
1014	1.08	2.58	0.69	1.5	1.13	1.07	1680	1680	117	66	121	64
1015	1.84	3.62	0.76	2.04	1.16	1.19	1477	1458	116	75	118	73
1016	1.22	5.12	1.39	2.72	1.09	1.22	1758	1814	129	78	106	59
1017	2.16	4.05	1.23	2.09	1.39	1.28	1482	1415	126	77	127	73
1018	0.91	3.77	1.15	2.07	1.17	1.22	1682	1716	130	82	133	80
1019	1.49	3.93	0.94	2.4	1.11	1.05	1824	1778	172	95	163	93
1020	1.62	2.74	0.7	1.62	1.04	1.11	1408	1414	139	93	126	92
1021	12.96	6.8	0.99	1.93	1.2	1.13	1461	1448	124	70	113	60
1022	4.7	3.97	0.75	2.07	0.53	0.59	1468	746	133	68	132	65

Number	CHD(1=Y	severity （(	IASBP	IASBP≥5	IASBP≥1	IASBP≥15mmHg（1=Yes，2=No）
1	0	1	1	2	2	2
2	0	1	5	1	2	2
3	1	3	1	2	2	2
4	1	2	-1	2	2	2
5	1	4	0	2	2	2
6	1	4	-6	1	2	2
7	0	1	-8	1	2	2
8	1	4	-4	2	2	2
9	1	4	-12	1	1	2
10	1	4	4	2	2	2
11	1	3	-6	1	2	2
12	1	3	8	1	2	2
13	1	3	0	2	2	2
14	1	3	0	2	2	2
15	1	4	-3	2	2	2
16	1	2	2	2	2	2
17	0	1	0	2	2	2
18	1	4	4	2	2	2
19	0	1	-3	2	2	2
20	1	4	0	2	2	2
21	1	4	-9	1	2	2
22	0	1	1	2	2	2
23	0	1	-4	2	2	2
24	1	3	6	1	2	2
25	1	3	-1	2	2	2
26	0	1	10	1	1	2
27	1	3	-2	2	2	2
28	0	0	0	2	2	2
29	1	3	3	2	2	2
30	1	3	3	2	2	2
31	1	4	8	1	2	2
32	1	4	5	1	2	2
33	1	4	7	1	2	2
34	1	3	8	1	2	2
35	0	1	6	1	2	2
36	0	1	1	2	2	2
37	1	4	-6	1	2	2

38	1	3	-6	1	2	2
39	1	4	4	2	2	2
40	1	2	4	2	2	2
41	1	4	-2	2	2	2
42	1	3	-8	1	2	2
43	1	1	2	2	2	2
44	0	1	-4	2	2	2
45	0	1	6	1	2	2
46	1	4	0	2	2	2
47	1	4	0	2	2	2
48	1	3	5	1	2	2
49	1	4	-4	2	2	2
50	1	4	5	1	2	2
51	1	4	1	2	2	2
52	1	4	-2	2	2	2
53	1	4	0	2	2	2
54	0	1	2	2	2	2
55	0	1	7	1	2	2
56	0	0	-4	2	2	2
57	1	2	3	2	2	2
58	1	2	3	2	2	2
59	1	3	-2	2	2	2
60	1	3	-5	1	2	2
61	1	4	0	2	2	2
62	0	1	-2	2	2	2
63	1	4	-3	2	2	2
64	1	3	-13	1	1	2
65	1	4	-7	1	2	2
66	1	4	-1	2	2	2
67	0	1	10	1	1	2
68	1	4	-3	2	2	2
69	1	4	3	2	2	2
70	1	3	8	1	2	2
71	0	1	0	2	2	2
72	1	3	0	2	2	2
73	1	4	-6	1	2	2
74	1	2	-2	2	2	2
75	1	3	-4	2	2	2

76	1	4	8	1	2	2
77	0	1	4	2	2	2
78	1	2	1	2	2	2
79	1	4	-8	1	2	2
80	0	1	0	2	2	2
81	1	3	5	1	2	2
82	1	4	-1	2	2	2
83	0	1	-7	1	2	2
84	1	3	0	2	2	2
85	1	2	-4	2	2	2
86	1	3	5	1	2	2
87	0	1	-18	1	1	1
88	1	3	-4	2	2	2
89	0	1	-8	1	2	2
90	1	4	-8	1	2	2
91	1	4	-2	2	2	2
92	1	3	-4	2	2	2
93	1	4	-5	1	2	2
94	1	3	7	1	2	2
95	1	4	3	2	2	2
96	1	4	4	2	2	2
97	1	4	-4	2	2	2
98	1	3	6	1	2	2
99	1	4	5	1	2	2
100	1	4	-5	1	2	2
101	1	3	-6	1	2	2
102	1	3	0	2	2	2
103	1	3	-1	2	2	2
104	1	3	-1	2	2	2
105	1	4	-13	1	1	2
106	0	1	-3	2	2	2
107	1	3	2	2	2	2
108	1	4	-6	1	2	2
109	1	4	2	2	2	2
110	1	4	-9	1	2	2
111	1	3	2	2	2	2
112	1	4	-2	2	2	2
113	1	4	3	2	2	2

114	0	1	-2	2	2	2
115	1	3	1	2	2	2
116	1	2	-1	2	2	2
117	0	1	2	2	2	2
118	0	1	0	2	2	2
119	1	4	2	2	2	2
120	1	4	-3	2	2	2
121	1	4	-15	1	1	1
122	1	4	4	2	2	2
123	1	4	-1	2	2	2
124	1	4	-3	2	2	2
125	1	4	-3	2	2	2
126	1	3	-1	2	2	2
127	1	4	2	2	2	2
128	1	4	5	1	2	2
129	1	4	0	2	2	2
130	0	1	-3	2	2	2
131	1	3	2	2	2	2
132	1	3	-3	2	2	2
133	0	1	-3	2	2	2
134	0	1	7	1	2	2
135	1	2	-1	2	2	2
136	1	4	3	2	2	2
137	0	1	-4	2	2	2
138	1	4	-6	1	2	2
139	0	1	1	2	2	2
140	1	3	5	1	2	2
141	0	1	-3	2	2	2
142	1	4	1	2	2	2
143	1	4	4	2	2	2
144	0	1	-7	1	2	2
145	1	4	-6	1	2	2
146	1	3	-5	1	2	2
147	1	2	-2	2	2	2
148	1	3	-1	2	2	2
149	0	1	1	2	2	2
150	0	1	7	1	2	2
151	0	1	-1	2	2	2

152	1	4	-1	2	2	2
153	1	4	5	1	2	2
154	0	1	3	2	2	2
155	1	3	-7	1	2	2
156	1	4	0	2	2	2
157	1	4	-4	2	2	2
158	1	3	1	2	2	2
159	1	3	-6	1	2	2
160	1	4	-4	2	2	2
161	1	3	0	2	2	2
162	1	3	-11	1	1	2
163	0	1	1	2	2	2
164	1	4	0	2	2	2
165	1	4	3	2	2	2
166	1	4	-5	1	2	2
167	0	1	3	2	2	2
168	0	1	1	2	2	2
169	1	4	4	2	2	2
170	1	3	6	1	2	2
171	1	3	-2	2	2	2
172	0	1	-5	1	2	2
173	1	3	11	1	1	2
174	1	3	-11	1	1	2
175	1	4	-7	1	2	2
176	1	4	-21	1	1	1
177	1	4	2	2	2	2
178	0	1	-9	1	2	2
179	1	3	4	2	2	2
180	1	4	-2	2	2	2
181	1	4	0	2	2	2
182	1	3	-1	2	2	2
183	0	1	6	1	2	2
184	1	4	11	1	1	2
185	0	1	0	2	2	2
186	1	3	3	2	2	2
187	1	4	-3	2	2	2
188	1	3	1	2	2	2
189	0	1	-2	2	2	2

190	1	4	4	2	2	2
191	1	4	-6	1	2	2
192	1	4	3	2	2	2
193	1	3	-3	2	2	2
194	1	4	1	2	2	2
195	0	1	0	2	2	2
196	1	4	-4	2	2	2
197	1	3	3	2	2	2
198	1	4	0	2	2	2
199	0	1	1	2	2	2
200	1	4	4	2	2	2
201	0	1	1	2	2	2
202	1	4	9	1	2	2
203	0	1	2	2	2	2
204	1	4	0	2	2	2
205	1	3	-2	2	2	2
206	1	2	-1	2	2	2
207	1	4	1	2	2	2
208	1	3	-7	1	2	2
209	1	4	2	2	2	2
210	1	3	-2	2	2	2
211	1	4	0	2	2	2
212	0	0	-1	2	2	2
213	1	4	2	2	2	2
214	1	4	-1	2	2	2
215	1	4	2	2	2	2
216	0	1	0	2	2	2
217	1	4	6	1	2	2
218	0	0	3	2	2	2
219	1	3	16	1	1	1
220	1	4	8	1	2	2
221	1	4	-2	2	2	2
222	1	3	-5	1	2	2
223	1	4	1	2	2	2
224	1	2	4	2	2	2
225	1	3	-7	1	2	2
226	1	2	1	2	2	2
227	1	3	35	1	1	1

228	1	2	1	2	2	2
229	1	3	7	1	2	2
230	1	3	1	2	2	2
231	1	4	2	2	2	2
232	1	3	2	2	2	2
233	0	1	4	2	2	2
234	0	0	-2	2	2	2
235	0	0	2	2	2	2
236	0	0	5	1	2	2
237	0	1	-2	2	2	2
238	0	1	1	2	2	2
239	0	1	2	2	2	2
240	0	0	-1	2	2	2
241	0	1	-8	1	2	2
242	1	3	-3	2	2	2
243	1	4	6	1	2	2
244	1	4	-4	2	2	2
245	1	4	-9	1	2	2
246	1	3	3	2	2	2
247	1	2	-6	1	2	2
248	1	4	2	2	2	2
249	1	3	-1	2	2	2
250	1	3	0	2	2	2
251	1	4	-9	1	2	2
252	1	4	-2	2	2	2
253	1	3	1	2	2	2
254	1	4	-8	1	2	2
255	1	4	-1	2	2	2
256	1	4	-2	2	2	2
257	0	1	5	1	2	2
258	1	4	-2	2	2	2
259	1	4	0	2	2	2
260	1	3	3	2	2	2
261	1	4	5	1	2	2
262	1	4	-6	1	2	2
263	1	4	2	2	2	2
264	1	3	-1	2	2	2
265	1	4	-2	2	2	2

266	1	4	0	2	2	2
267	1	4	2	2	2	2
268	1	4	-1	2	2	2
269	1	4	1	2	2	2
270	1	4	-11	1	1	2
271	1	4	1	2	2	2
272	1	4	2	2	2	2
273	1	3	3	2	2	2
274	1	3	-10	1	1	2
275	1	2	-2	2	2	2
276	1	2	1	2	2	2
277	0	1	4	2	2	2
278	1	2	0	2	2	2
279	0	1	1	2	2	2
280	1	4	1	2	2	2
281	1	4	0	2	2	2
282	1	4	-25	1	1	1
283	1	3	1	2	2	2
284	1	3	-5	1	2	2
285	1	2	4	2	2	2
286	1	4	8	1	2	2
287	1	4	3	2	2	2
288	0	1	-8	1	2	2
289	1	4	-5	1	2	2
290	1	4	1	2	2	2
291	1	3	0	2	2	2
292	1	3	2	2	2	2
293	1	3	-5	1	2	2
294	1	3	-8	1	2	2
295	1	4	2	2	2	2
296	1	2	1	2	2	2
297	1	4	24	1	1	1
298	1	4	2	2	2	2
299	1	2	-2	2	2	2
300	1	4	15	1	1	1
301	0	1	-5	1	2	2
302	1	3	-5	1	2	2
303	0	1	2	2	2	2

304	1	2	6	1	2	2
305	0	1	-2	2	2	2
306	1	2	-2	2	2	2
307	0	1	1	2	2	2
308	0	1	3	2	2	2
309	0	1	3	2	2	2
310	0	1	1	2	2	2
311	0	1	0	2	2	2
312	1	2	-19	1	1	1
313	0	1	-6	1	2	2
314	0	1	0	2	2	2
315	0	1	-5	1	2	2
316	0	1	1	2	2	2
317	0	1	2	2	2	2
318	0	1	-2	2	2	2
319	0	1	5	1	2	2
320	0	1	-1	2	2	2
321	0	1	1	2	2	2
322	0	1	2	2	2	2
323	0	1	-1	2	2	2
324	0	1	1	2	2	2
325	0	1	3	2	2	2
326	0	1	1	2	2	2
327	0	1	8	1	2	2
328	0	1	-7	1	2	2
329	0	1	-4	2	2	2
330	0	1	1	2	2	2
331	0	1	21	1	1	1
332	0	1	-2	2	2	2
333	0	1	2	2	2	2
334	0	1	5	1	2	2
335	0	1	0	2	2	2
336	0	1	-2	2	2	2
337	0	1	0	2	2	2
338	0	1	-1	2	2	2
339	0	1	-3	2	2	2
340	0	1	-1	2	2	2
341	0	0	-6	1	2	2

342	1	4	-3	2	2	2
343	1	4	-8	1	2	2
344	1	4	2	2	2	2
345	0	1	-2	2	2	2
346	1	3	2	2	2	2
347	1	4	5	1	2	2
348	1	4	6	1	2	2
349	1	3	2	2	2	2
350	1	2	2	2	2	2
351	1	4	-4	2	2	2
352	1	4	-1	2	2	2
353	1	4	5	1	2	2
354	1	4	-6	1	2	2
355	1	4	-1	2	2	2
356	0	1	-10	1	1	2
357	0	1	1	2	2	2
358	1	4	-3	2	2	2
359	1	4	-5	1	2	2
360	1	3	-5	1	2	2
361	1	4	8	1	2	2
362	1	2	-1	2	2	2
363	1	3	-4	2	2	2
364	1	4	-5	1	2	2
365	1	2	-1	2	2	2
366	1	4	5	1	2	2
367	1	3	-1	2	2	2
368	1	4	-7	1	2	2
369	1	2	-1	2	2	2
370	1	3	-1	2	2	2
371	1	3	-3	2	2	2
372	1	4	-4	2	2	2
373	1	4	-20	1	1	1
374	1	4	11	1	1	2
375	1	4	-1	2	2	2
376	1	4	-8	1	2	2
377	1	4	0	2	2	2
378	1	4	2	2	2	2
379	1	4	-2	2	2	2

380	1	3	-4	2	2	2
381	1	4	1	2	2	2
382	1	2	-6	1	2	2
383	1	4	3	2	2	2
384	1	3	0	2	2	2
385	1	1	-2	2	2	2
386	1	4	6	1	2	2
387	1	4	0	2	2	2
388	1	4	-3	2	2	2
389	1	3	-3	2	2	2
390	1	2	0	2	2	2
391	1	4	4	2	2	2
392	1	3	22	1	1	1
393	1	3	-6	1	2	2
394	1	3	-8	1	2	2
395	1	2	9	1	2	2
396	1	3	-1	2	2	2
397	1	3	-2	2	2	2
398	1	3	-3	2	2	2
399	1	4	3	2	2	2
400	1	4	1	2	2	2
401	1	4	5	1	2	2
402	1	4	6	1	2	2
403	1	2	2	2	2	2
404	0	1	5	1	2	2
405	1	4	-1	2	2	2
406	1	4	-3	2	2	2
407	1	4	2	2	2	2
408	1	4	0	2	2	2
409	1	4	-2	2	2	2
410	1	4	2	2	2	2
411	1	2	-1	2	2	2
412	1	4	4	2	2	2
413	1	3	2	2	2	2
414	1	4	2	2	2	2
415	0	1	1	2	2	2
416	1	3	-1	2	2	2
417	1	2	4	2	2	2

418	1	4	-1	2	2	2
419	1	3	0	2	2	2
420	1	4	8	1	2	2
421	1	3	5	1	2	2
422	1	4	7	1	2	2
423	1	3	1	2	2	2
424	1	3	-9	1	2	2
425	1	3	4	2	2	2
426	1	4	4	2	2	2
427	1	4	-2	2	2	2
428	1	4	-3	2	2	2
429	1	4	2	2	2	2
430	1	3	9	1	2	2
431	1	3	-1	2	2	2
432	1	3	5	1	2	2
433	1	3	2	2	2	2
434	1	3	-1	2	2	2
435	1	4	0	2	2	2
436	1	3	-4	2	2	2
437	1	3	4	2	2	2
438	1	3	1	2	2	2
439	1	4	0	2	2	2
440	1	4	10	1	1	2
441	1	4	-2	2	2	2
442	1	3	-2	2	2	2
443	1	4	2	2	2	2
444	1	3	-5	1	2	2
445	1	4	0	2	2	2
446	1	4	1	2	2	2
447	1	4	-6	1	2	2
448	1	4	-1	2	2	2
449	1	3	-4	2	2	2
450	1	3	-1	2	2	2
451	1	3	-7	1	2	2
452	1	4	-6	1	2	2
453	0	1	-1	2	2	2
454	1	4	-4	2	2	2
455	1	3	-2	2	2	2

456	1	4	-4	2	2	2
457	1	2	1	2	2	2
458	1	3	4	2	2	2
459	1	3	2	2	2	2
460	1	4	2	2	2	2
461	1	4	-2	2	2	2
462	1	2	3	2	2	2
463	1	3	-4	2	2	2
464	1	3	2	2	2	2
465	1	3	-1	2	2	2
466	1	4	0	2	2	2
467	1	3	2	2	2	2
468	1	4	-4	2	2	2
469	1	3	0	2	2	2
470	1	3	7	1	2	2
471	1	3	7	1	2	2
472	1	3	0	2	2	2
473	1	4	-5	1	2	2
474	1	3	1	2	2	2
475	1	3	5	1	2	2
476	1	3	-2	2	2	2
477	1	3	-1	2	2	2
478	1	3	12	1	1	2
479	1	4	-4	2	2	2
480	1	4	13	1	1	2
481	1	4	-12	1	1	2
482	1	3	5	1	2	2
483	1	4	2	2	2	2
484	1	4	19	1	1	1
485	1	3	5	1	2	2
486	0	1	0	2	2	2
487	0	1	1	2	2	2
488	0	1	3	2	2	2
489	0	1	-2	2	2	2
490	0	1	2	2	2	2
491	0	1	-2	2	2	2
492	0	1	-6	1	2	2
493	0	0	-4	2	2	2

494	0	1	2	2	2	2
495	0	1	7	1	2	2
496	0	1	-3	2	2	2
497	0	1	-4	2	2	2
498	0	1	0	2	2	2
499	0	1	7	1	2	2
500	0	1	-2	2	2	2
501	0	1	6	1	2	2
502	0	1	2	2	2	2
503	0	1	2	2	2	2
504	0	1	3	2	2	2
505	0	1	-2	2	2	2
506	0	1	-4	2	2	2
507	0	1	3	2	2	2
508	0	1	3	2	2	2
509	0	1	-7	1	2	2
510	0	0	3	2	2	2
511	0	1	-1	2	2	2
512	0	1	2	2	2	2
513	0	1	1	2	2	2
514	0	1	7	1	2	2
515	0	1	-2	2	2	2
516	0	1	2	2	2	2
517	0	1	-1	2	2	2
518	0	1	-2	2	2	2
519	0	1	0	2	2	2
520	0	1	3	2	2	2
521	1	3	0	2	2	2
522	0	1	3	2	2	2
523	0	1	-1	2	2	2
524	0	1	-3	2	2	2
525	0	1	2	2	2	2
526	0	1	0	2	2	2
527	1	2	-7	1	2	2
528	0	1	6	1	2	2
529	1	2	4	2	2	2
530	1	4	-8	1	2	2
531	0	1	8	1	2	2

532	1	3	0	2	2	2
533	1	3	-6	1	2	2
534	1	3	5	1	2	2
535	1	2	0	2	2	2
536	1	2	8	1	2	2
537	0	1	3	2	2	2
538	1	3	-6	1	2	2
539	1	3	2	2	2	2
540	1	4	4	2	2	2
541	1	2	7	1	2	2
542	1	2	7	1	2	2
543	1	2	4	2	2	2
544	1	2	-6	1	2	2
545	1	4	5	1	2	2
546	1	3	-5	1	2	2
547	1	3	-31	1	1	1
548	1	3	2	2	2	2
549	1	4	-1	2	2	2
550	1	3	20	1	1	1
551	1	4	-1	2	2	2
552	1	3	-2	2	2	2
553	1	2	-4	2	2	2
554	1	4	-6	1	2	2
555	1	4	0	2	2	2
556	1	2	2	2	2	2
557	1	3	-1	2	2	2
558	1	2	-6	1	2	2
559	1	2	-2	2	2	2
560	1	4	2	2	2	2
561	1	3	-9	1	2	2
562	1	4	11	1	1	2
563	1	4	1	2	2	2
564	0	1	-12	1	1	2
565	1	4	21	1	1	1
566	1	4	4	2	2	2
567	1	4	1	2	2	2
568	1	4	-3	2	2	2
569	1	4	8	1	2	2

570	1	4	1	2	2	2
571	1	4	-22	1	1	1
572	1	3	-7	1	2	2
573	1	3	-3	2	2	2
574	1	2	-3	2	2	2
575	1	4	-2	2	2	2
576	0	1	-9	1	2	2
577	1	3	7	1	2	2
578	1	3	-1	2	2	2
579	1	2	-1	2	2	2
580	1	4	-3	2	2	2
581	1	3	-2	2	2	2
582	0	1	3	2	2	2
583	1	2	1	2	2	2
584	1	3	-3	2	2	2
585	1	4	-4	2	2	2
586	1	4	2	2	2	2
587	0	1	2	2	2	2
588	0	1	-1	2	2	2
589	0	0	4	2	2	2
590	0	0	2	2	2	2
591	0	0	0	2	2	2
592	0	0	1	2	2	2
593	0	0	-1	2	2	2
594	1	3	0	2	2	2
595	1	4	8	1	2	2
596	1	3	-3	2	2	2
597	1	4	-1	2	2	2
598	1	4	0	2	2	2
599	1	3	-5	1	2	2
600	1	2	1	2	2	2
601	1	3	-5	1	2	2
602	1	3	3	2	2	2
603	1	3	-1	2	2	2
604	1	4	-15	1	1	1
605	1	3	0	2	2	2
606	1	3	-1	2	2	2
607	1	4	-5	1	2	2

608	1	3	-4	2	2	2
609	1	3	2	2	2	2
610	1	4	1	2	2	2
611	1	3	2	2	2	2
612	1	4	-2	2	2	2
613	1	4	-2	2	2	2
614	1	4	-8	1	2	2
615	1	3	0	2	2	2
616	1	3	1	2	2	2
617	1	4	3	2	2	2
618	1	4	1	2	2	2
619	1	4	-2	2	2	2
620	1	4	-1	2	2	2
621	1	4	1	2	2	2
622	1	4	1	2	2	2
623	1	4	-2	2	2	2
624	1	4	2	2	2	2
625	1	4	4	2	2	2
626	1	3	-2	2	2	2
627	1	4	3	2	2	2
628	1	3	3	2	2	2
629	1	4	-1	2	2	2
630	1	4	7	1	2	2
631	1	4	0	2	2	2
632	1	3	-1	2	2	2
633	1	4	3	2	2	2
634	1	3	7	1	2	2
635	1	4	-3	2	2	2
636	1	4	6	1	2	2
637	1	4	2	2	2	2
638	1	3	1	2	2	2
639	1	4	1	2	2	2
640	1	3	-8	1	2	2
641	1	3	0	2	2	2
642	1	4	-9	1	2	2
643	1	4	0	2	2	2
644	1	3	0	2	2	2
645	1	4	-1	2	2	2

646	1	2	4	2	2	2
647	1	3	2	2	2	2
648	1	4	4	2	2	2
649	1	4	3	2	2	2
650	1	3	1	2	2	2
651	1	4	8	1	2	2
652	1	4	5	1	2	2
653	1	4	6	1	2	2
654	1	4	-2	2	2	2
655	1	2	-1	2	2	2
656	1	3	-7	1	2	2
657	1	4	-1	2	2	2
658	1	3	1	2	2	2
659	1	4	-12	1	1	2
660	1	4	-3	2	2	2
661	1	4	-21	1	1	1
662	1	3	2	2	2	2
663	1	4	-4	2	2	2
664	1	3	-5	1	2	2
665	1	4	-8	1	2	2
666	1	4	3	2	2	2
667	1	3	6	1	2	2
668	1	2	-6	1	2	2
669	1	4	-2	2	2	2
670	1	4	-6	1	2	2
671	1	4	-1	2	2	2
672	1	4	2	2	2	2
673	1	3	-7	1	2	2
674	1	4	-1	2	2	2
675	1	2	-7	1	2	2
676	1	4	0	2	2	2
677	0	0	5	1	2	2
678	0	1	-1	2	2	2
679	0	1	4	2	2	2
680	0	1	-3	2	2	2
681	0	1	1	2	2	2
682	0	1	-2	2	2	2
683	0	1	1	2	2	2

684	0	1	-2	2	2	2
685	0	1	-1	2	2	2
686	0	1	3	2	2	2
687	0	0	0	2	2	2
688	0	1	0	2	2	2
689	0	0	-3	2	2	2
690	0	1	-4	2	2	2
691	1	2	-7	1	2	2
692	0	1	3	2	2	2
693	0	1	-1	2	2	2
694	0	1	2	2	2	2
695	0	1	-3	2	2	2
696	0	1	1	2	2	2
697	0	1	2	2	2	2
698	0	1	-3	2	2	2
699	0	1	-4	2	2	2
700	0	1	-10	1	1	2
701	0	1	-1	2	2	2
702	0	1	-2	2	2	2
703	1	4	15	1	1	1
704	1	4	-3	2	2	2
705	1	4	-3	2	2	2
706	1	4	-9	1	2	2
707	1	4	-6	1	2	2
708	1	3	1	2	2	2
709	1	3	3	2	2	2
710	1	4	-2	2	2	2
711	0	1	-11	1	1	2
712	1	3	-2	2	2	2
713	1	4	-20	1	1	1
714	1	2	-3	2	2	2
715	1	3	-5	1	2	2
716	1	3	-2	2	2	2
717	1	3	4	2	2	2
718	1	3	0	2	2	2
719	0	0	2	2	2	2
720	1	4	-2	2	2	2
721	1	4	-1	2	2	2

722	1	4	-5	1	2	2
723	1	4	1	2	2	2
724	1	3	1	2	2	2
725	1	4	-12	1	1	2
726	1	4	0	2	2	2
727	1	2	-3	2	2	2
728	1	2	-5	1	2	2
729	1	3	4	2	2	2
730	1	3	-3	2	2	2
731	1	3	4	2	2	2
732	1	4	2	2	2	2
733	1	4	-10	1	1	2
734	1	3	17	1	1	1
735	1	4	-19	1	1	1
736	1	4	-6	1	2	2
737	1	4	4	2	2	2
738	1	4	-5	1	2	2
739	1	3	-2	2	2	2
740	1	4	4	2	2	2
741	1	3	4	2	2	2
742	1	2	-4	2	2	2
743	1	3	3	2	2	2
744	1	3	-6	1	2	2
745	1	3	4	2	2	2
746	1	3	-4	2	2	2
747	1	4	2	2	2	2
748	1	2	-5	1	2	2
749	1	4	-3	2	2	2
750	1	3	0	2	2	2
751	0	1	2	2	2	2
752	0	0	1	2	2	2
753	0	0	-5	1	2	2
754	0	1	-3	2	2	2
755	0	0	0	2	2	2
756	0	1	-2	2	2	2
757	0	0	-2	2	2	2
758	1	4	-1	2	2	2
759	1	4	-2	2	2	2

760	1	4	-11	1	1	2
761	1	3	5	1	2	2
762	1	4	4	2	2	2
763	1	3	0	2	2	2
764	1	4	7	1	2	2
765	1	4	11	1	1	2
766	1	4	0	2	2	2
767	1	4	1	2	2	2
768	1	2	-4	2	2	2
769	1	4	7	1	2	2
770	1	3	0	2	2	2
771	1	3	-2	2	2	2
772	1	4	9	1	2	2
773	1	4	1	2	2	2
774	1	4	-3	2	2	2
775	1	4	-4	2	2	2
776	1	3	-8	1	2	2
777	1	4	-2	2	2	2
778	1	4	-5	1	2	2
779	1	3	1	2	2	2
780	1	4	-6	1	2	2
781	1	4	0	2	2	2
782	1	4	-2	2	2	2
783	1	4	-7	1	2	2
784	1	2	5	1	2	2
785	1	4	2	2	2	2
786	1	4	-4	2	2	2
787	1	3	-4	2	2	2
788	1	4	-2	2	2	2
789	1	3	-4	2	2	2
790	1	4	3	2	2	2
791	1	3	0	2	2	2
792	1	4	2	2	2	2
793	1	3	1	2	2	2
794	1	3	-5	1	2	2
795	1	4	-3	2	2	2
796	1	4	-3	2	2	2
797	1	4	-5	1	2	2

798	0	1	-16	1	1	1
799	1	4	5	1	2	2
800	1	4	1	2	2	2
801	1	4	0	2	2	2
802	0	1	5	1	2	2
803	1	3	13	1	1	2
804	1	4	4	2	2	2
805	1	3	-16	1	1	1
806	0	1	4	2	2	2
807	1	4	-5	1	2	2
808	1	2	0	2	2	2
809	1	4	-7	1	2	2
810	0	1	2	2	2	2
811	1	4	-3	2	2	2
812	1	4	-4	2	2	2
813	1	4	4	2	2	2
814	1	4	-2	2	2	2
815	1	3	1	2	2	2
816	1	2	-5	1	2	2
817	1	4	5	1	2	2
818	1	3	-2	2	2	2
819	1	4	4	2	2	2
820	1	4	5	1	2	2
821	1	4	-1	2	2	2
822	1	3	6	1	2	2
823	1	3	4	2	2	2
824	1	3	0	2	2	2
825	1	4	-13	1	1	2
826	0	1	2	2	2	2
827	1	4	0	2	2	2
828	1	4	4	2	2	2
829	1	4	1	2	2	2
830	1	4	2	2	2	2
831	1	4	2	2	2	2
832	1	3	1	2	2	2
833	1	3	1	2	2	2
834	1	3	-4	2	2	2
835	1	3	1	2	2	2

836	1	4	-4	2	2	2
837	1	4	0	2	2	2
838	1	4	-7	1	2	2
839	1	4	1	2	2	2
840	1	3	-2	2	2	2
841	1	2	2	2	2	2
842	1	4	2	2	2	2
843	1	3	3	2	2	2
844	1	4	1	2	2	2
845	1	4	-1	2	2	2
846	1	2	5	1	2	2
847	1	3	1	2	2	2
848	1	2	-9	1	2	2
849	1	4	-5	1	2	2
850	1	4	1	2	2	2
851	1	4	-2	2	2	2
852	1	3	-2	2	2	2
853	1	3	-7	1	2	2
854	1	4	0	2	2	2
855	1	4	-1	2	2	2
856	0	1	-3	2	2	2
857	0	1	5	1	2	2
858	0	1	3	2	2	2
859	0	1	0	2	2	2
860	0	1	-1	2	2	2
861	0	1	-1	2	2	2
862	0	0	2	2	2	2
863	0	1	3	2	2	2
864	0	1	-3	2	2	2
865	0	1	-1	2	2	2
866	0	1	7	1	2	2
867	0	1	3	2	2	2
868	0	1	6	1	2	2
869	0	1	0	2	2	2
870	0	1	8	1	2	2
871	0	1	-8	1	2	2
872	0	1	2	2	2	2
873	0	1	-5	1	2	2

874	0	1	4	2	2	2
875	0	1	5	1	2	2
876	0	1	-9	1	2	2
877	0	1	2	2	2	2
878	0	1	-1	2	2	2
879	0	1	0	2	2	2
880	0	1	0	2	2	2
881	0	1	9	1	2	2
882	0	1	2	2	2	2
883	0	1	-1	2	2	2
884	0	0	4	2	2	2
885	1	4	0	2	2	2
886	1	4	0	2	2	2
887	1	4	-1	2	2	2
888	1	3	1	2	2	2
889	1	2	2	2	2	2
890	1	3	7	1	2	2
891	1	2	-1	2	2	2
892	1	3	-2	2	2	2
893	1	4	-9	1	2	2
894	1	2	-4	2	2	2
895	1	2	2	2	2	2
896	1	4	-3	2	2	2
897	1	4	-5	1	2	2
898	1	3	-6	1	2	2
899	1	3	-2	2	2	2
900	1	4	-3	2	2	2
901	1	3	-6	1	2	2
902	1	4	-6	1	2	2
903	1	3	-1	2	2	2
904	0	1	2	2	2	2
905	1	4	2	2	2	2
906	1	4	-2	2	2	2
907	1	3	-2	2	2	2
908	1	3	8	1	2	2
909	1	4	5	1	2	2
910	1	2	-2	2	2	2
911	1	2	-1	2	2	2

912	1	4	3	2	2	2
913	0	0	-3	2	2	2
914	1	3	-1	2	2	2
915	1	2	-6	1	2	2
916	1	4	15	1	1	1
917	1	4	-4	2	2	2
918	1	4	-3	2	2	2
919	1	4	2	2	2	2
920	0	1	3	2	2	2
921	1	4	-4	2	2	2
922	1	3	-1	2	2	2
923	1	2	2	2	2	2
924	1	2	3	2	2	2
925	1	4	3	2	2	2
926	1	4	-3	2	2	2
927	1	4	4	2	2	2
928	1	2	-4	2	2	2
929	1	3	4	2	2	2
930	0	1	-1	2	2	2
931	1	4	1	2	2	2
932	1	4	-1	2	2	2
933	1	4	4	2	2	2
934	1	4	2	2	2	2
935	1	4	-9	1	2	2
936	1	3	-1	2	2	2
937	1	4	0	2	2	2
938	1	2	2	2	2	2
939	1	4	-9	1	2	2
940	1	4	6	1	2	2
941	1	4	5	1	2	2
942	0	1	1	2	2	2
943	0	0	-2	2	2	2
944	0	0	-5	1	2	2
945	0	0	-2	2	2	2
946	0	0	0	2	2	2
947	0	0	3	2	2	2
948	0	0	4	2	2	2
949	0	0	2	2	2	2

950	0	0	1	2	2	2
951	0	0	1	2	2	2
952	1	3	-2	2	2	2
953	1	4	3	2	2	2
954	1	3	-5	1	2	2
955	1	3	6	1	2	2
956	1	4	-6	1	2	2
957	1	3	3	2	2	2
958	1	4	-2	2	2	2
959	1	2	-6	1	2	2
960	1	2	15	1	1	1
961	1	4	-1	2	2	2
962	1	4	-7	1	2	2
963	1	4	1	2	2	2
964	1	2	-3	2	2	2
965	1	4	5	1	2	2
966	1	4	0	2	2	2
967	1	4	-16	1	1	1
968	1	2	-28	1	1	1
969	1	4	31	1	1	1
970	1	4	13	1	1	2
971	1	3	25	1	1	1
972	1	4	-16	1	1	1
973	0	0	-10	1	1	2
974	1	3	16	1	1	1
975	1	3	10	1	1	2
976	1	4	10	1	1	2
977	1	2	0	2	2	2
978	1	4	-5	1	2	2
979	1	4	3	2	2	2
980	1	2	-1	2	2	2
981	1	2	-1	2	2	2
982	1	3	0	2	2	2
983	1	4	-6	1	2	2
984	1	3	4	2	2	2
985	1	3	7	1	2	2
986	1	2	9	1	2	2
987	1	4	0	2	2	2

988	1	4	4	2	2	2
989	1	3	-2	2	2	2
990	1	3	10	1	1	2
991	1	4	-12	1	1	2
992	1	3	-2	2	2	2
993	1	2	0	2	2	2
994	1	2	0	2	2	2
995	1	2	26	1	1	1
996	0	1	0	2	2	2
997	0	0	6	1	2	2
998	1	2	4	2	2	2
999	0	1	-5	1	2	2
1000	1	2	2	2	2	2
1001	1	4	0	2	2	2
1002	1	3	1	2	2	2
1003	1	2	-1	2	2	2
1004	1	4	3	2	2	2
1005	1	4	2	2	2	2
1006	1	2	2	2	2	2
1007	0	0	7	1	2	2
1008	1	4	-8	1	2	2
1009	1	4	-36	1	1	1
1010	1	4	9	1	2	2
1011	1	2	3	2	2	2
1012	1	3	4	2	2	2
1013	1	2	-2	2	2	2
1014	1	2	-4	2	2	2
1015	1	3	-2	2	2	2
1016	1	4	23	1	1	1
1017	1	2	-1	2	2	2
1018	1	4	-3	2	2	2
1019	0	0	9	1	2	2
1020	1	2	13	1	1	2
1021	1	4	11	1	1	2
1022	1	4	1	2	2	2
