# Supplementary material for: Elevated Interarm Systolic Blood Pressure Difference Is Positively Associated with Increased Likelihood of Coronary Artery Disease
Source: Int J Hypertens. 2021 Jul 21;2021:5577957. doi: 10.1155/2021/5577957 (PMC8321749; doi:10.1155/2021/5577957)
Supplement: Supplementary Materials — Supplementary Table 1: relationship between IASBPD and coronary artery disease using a univariate logistic regression model. Supplementary Table 2: relationship between IASBPD and coronary artery disease using a multiple logistic regression model. Original data: mainly recorded the original data related to this article. [file 5577957.f1.zip › Supplementary Tables.docx]

**Supplementary Table 1. Relationship between IASBPD and coronary artery disease using a univariate logistic regression model**

|  | OR (95%CI) | P Value |
| --- | --- | --- |
| Sex (Female as reference) | 2.184 (1.461-3.265) | 0.001 |
| Age | 1.020 (1.004-1.036) | 0.015 |
| Overweight | 1.529 (1.018-2.298) | 0.041 |
| Obesity | 1.632 (1.144-2.328) | 0.007 |
| Hypertension | 1.873 (1.347-2.604) | 0.001 |
| Diabetes | 1.601 (1.154-2.222) | 0.005 |
| Smoking | 1.102 (0.736-1.652) | 0.637 |
| HDL-C﹤1.0mmol/L | 1.347 (0.982-1.849) | 0.065 |
| LDL-C ≥ 4.1 mmol/l | 1.577 (0.649-3.830) | 0.315 |
| IASBPD ≥ 10mmHg | 2.136 (1.052-4.338) | 0.036 |

Abbreviations: CI, confidence interval; HDL-C, high-density lipoprotein cholesterol; IASBPD, interarm systolic blood pressure difference; LDL-C, low-density lipoprotein cholesterol; OR, odds ratio.

**Supplementary Table 2. Relationship between IASBPD and coronary artery disease using a multiple logistic regression model**

|  | OR (95%CI) | P Value |
| --- | --- | --- |
| Age | 1.018 (1.002-1.034) | 0.027 |
| Antihypertension drugs | 1.402 (0.859-2.287) | 0.176 |
| Lipid-lowering drugs | 1.724 (1.271-2.338) | 0.001 |
| Male | 2.241 (1.493-3.366) | 0.001 |
| Overweight | 1.061 (0.727-1.548) | 0.761 |
| Obesity | 0.627 (0.416-0.945) | 0.026 |
| Hypertension | 1.358 (0.808-2.281) | 0.248 |
| Diabetes | 1.520 (1.092-2.115) | 0.013 |
| Smoking | 1.059 (0.704-1.594) | 0.782 |
| TG﹥2.3mmol/L | 0.870 (0.596-1.271) | 0.471 |
| HDL-C﹤1.0mmol/L | 1.366 (0.993-1.878) | 0.055 |
| LDL-C ≥ 4.1 mmol/l | 1.743 (0.715-4.252) | 0.222 |
| IASBPD ≥ 10mmHg] | 2.213 (1.086-4.509) | 0.029 |

Abbreviations: CI, confidence interval; HDL-C, high-density lipoprotein cholesterol; IASBPD, interarm systolic blood pressure difference; LDL-C, low-density lipoprotein cholesterol; OR, odds ratio; TG, triglycerides.
